# A Cyclometalated NHC Iridium Complex Bearing a Cationic (η^5^‐Cyclopentadienyl)(η^6^‐phenyl)iron Backbone**

**DOI:** 10.1002/chem.202102520

**Published:** 2021-10-05

**Authors:** Christian Malchau, Tom Milbert, Tobias R. Eger, Daniela V. Fries, Pascal J. Pape, Benjamin Oelkers, Yu Sun, Sabine Becker, Marc H. Prosenc, Gereon Niedner‐Schatteburg, Werner R. Thiel

**Affiliations:** ^1^ Fachbereich Chemie Technische Universität Kaiserslautern Erwin-Schrödinger-Straße 54 67663 Kaiserslautern Germany; ^2^ Fachbereich Chemie, Research Center OPTIMAS Technische Universität Kaiserslautern Erwin-Schrödinger-Straße 52 67663 Kaiserslautern Germany

**Keywords:** cyclometallation, iridium, iron, N-heterocyclic carbenes, transfer hydrogenation

## Abstract

Nucleophilic substitution of [(η^5^‐cyclopentadienyl)(η^6^‐chlorobenzene)iron(II)] hexafluorophosphate with sodium imidazolate resulted in the formation of [(η^5^‐cyclopentadienyl)(η^6^‐phenyl)iron(II)]imidazole hexafluorophosphate. The corresponding dicationic imidazolium salt, which was obtained by treating this imidazole precursor with methyl iodide, underwent cyclometallation with bis[dichlorido(η^5^‐1,2,3,4,5‐pentamethylcyclopentadienyl]iridium(III) in the presence of triethyl amine. The resulting bimetallic iridium(III) complex is the first example of an NHC complex bearing a cationic and cyclometallated [(η^5^‐cyclopentadienyl)(η^6^‐phenyl)iron(II)]^+^ substituent. As its iron(II) precursors, the bimetallic iridium(III) complex was fully characterized by means of spectroscopy, elemental analysis and single crystal X‐ray diffraction. In addition, it was investigated in a catalytic study, wherein it showed high activity in transfer hydrogenation compared to its neutral analogue having a simple phenyl instead of a cationic [(η^5^‐cyclopentadienyl)(η^6^‐phenyl)iron(II)]^+^ unit at the NHC ligand.

## Graphical abstract

**Iron power**: A cyclometalated Fe(II) and Ir(III) complex based on the cationic (η^5^‐cyclopentadienyl)(η^6^‐phenyl)iron(II)]imidazole skeleton is reported. Compared to its neutral analogue without the [CpFe]^+^ fragment, it is surprisingly active in the transfer hydrogenation of ketones. Mechanistic investigation on the reaction paths performed by DFT calculations provided a better understanding of the reaction mechanism, possible intermediates and the active species.

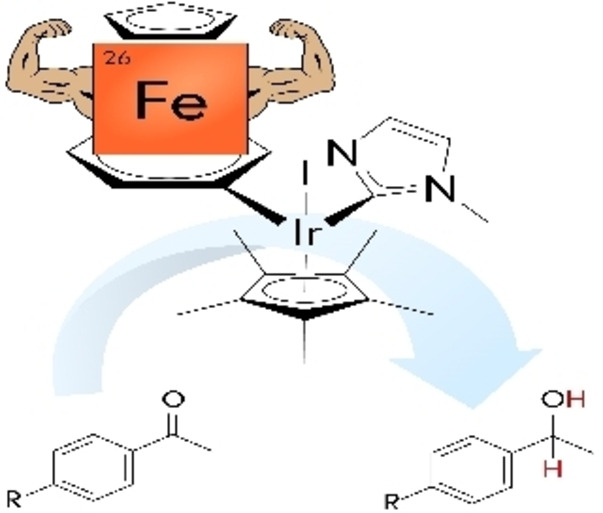

## Introduction

N‐Heterocyclic carbenes, commonly abbreviated as NHCs, are an important class of ligands for organometallic complexes, which became established in the 1960s until late 1970s.^[1]^ They are characterized as strong σ‐donors and are therefore resembling phosphines in terms of their ligand properties.^[2]^ Depending on their structure they are often more resistant to air, moisture and heat.^[3]^

The development of new NHC‐based catalysts accelerated in particular in the late 1990s.^[4]^ The certainly most prominent examples are the second‐generation Grubbs catalysts for olefin metathesis, which were obtained from the phosphine‐substituted species of the first generation.^[5]^ However, the structural motif of an NHC can also be employed to generate cyclometalated transition metal complexes.^[6]^ The resulting compounds are defined by the presence of at least one carbon‐metal bond in addition to the NHC‐metal bond.^[7]^ Cyclometalated iridium(III) compounds in particular are well known for specific applications in photochemistry and as antibacterial and anticancer agents in biology.^[8]^ In general, they are easily accessible via a base‐assisted C−H activation step.^[9]^ Numerous examples for the use of cyclometalated iridium(III) complexes in homogenous catalysis have been reported in the past: hydrogenation,^[10]^ transfer hydrogenation,^[11]^ dehydrogenation,[^11c^, ^12^] reductive amination,^[13]^ hydroamination,^[14]^ hydrosilylation[^11c^, ^15^] and racemization reactions^[16]^ are catalyzed by iridium compounds.

The application of transition metal complexes as catalysts for the transfer hydrogenation is known since 1967.^[17]^ At least since the trailblazing work of Noyori et al. from 1995 and the following years, catalytic transfer hydrogenation has developed into a highly regarded field of chemical research.^[18]^ In comparison to conventional hydrogenation, hazardous hydrogen and expensive pressure vessels can be avoided.^[11a]^ The challenge is then to design suitable catalysts bringing the reaction to equilibrium as fast as possible.^[19]^ Ruthenium is by far the most commonly used transition metal to mediate hydrogen transfer, though iridium catalysts are in general the most active ones.^[19c]^ Pioneering work in the field of iridium catalyzed transfer hydrogenation was reported by Mestroni et al. in the late 1970s.^[20]^ Since then, numerous examples have been reported. While most transfer hydrogenation catalysts are electronically neutral, there are also some cationic representatives.^[21]^ One of the most famous ones might be Crabtree's catalyst invented in the 1970s.^[22]^ The usage of iridium‐NHC complexes in transfer hydrogenation in general is even more widespread.^[23]^

In 2017, Choudhury et al. prepared a broad variety of five‐ and six‐membered cyclometalated iridium imidazolylidene complexes and have introduced them as catalysts for transfer hydrogenation.^[24]^ Their study was focused in particular on the stereoelectronic properties of such catalysts by comparison of their activities in a model transfer hydrogenation reaction. The cyclometalated complex derived from 3‐methyl‐1‐phenyl‐1*H*‐imidazol‐3‐ium iodide showed moderate activity yielding less than 40 % of racemic 1‐phenylethanol after 3 h at a reaction temperature of 100 °C and with the use of 20 mol‐% KOH as the base and isopropanol as the hydrogen source. The complex is therefore considered as a catalytically less active structural motif compared to various six‐membered iridacycles providing more efficient hydrogen transfer. To the best of our knowledge, this is the only catalytic application that has been reported with this compound to date.

Our group recently investigated cationic [(η^6^‐arene)(η^5^‐cyclopentadienyl)iron(II)]^+^ complexes as part of a ligand's backbone. Such 18 VE compounds were first described in the late 1950s.^[25]^ In addition to some cationic phosphines that are structurally related to commonly used triphenylphosphine, our interest turned on the possibility of generating bimetallic N‐heterocyclic carbene complexes based on the cationic [(η^6^‐arene)(η^5^‐cyclopentadienyl)iron(II)]^+^ motif.^[26]^

## Results and Discussion

### Synthesis of the dinuclear iridium(III)/iron(II) complex

**Scheme 1:**
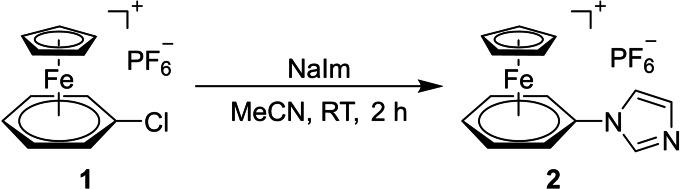
Synthesis of the imidazole complex **2**.

In 1992, Roberts had reported some imidazole and triazole derivatives with [(η^6^‐arene)(η^5^‐cyclopentadienyl)iron(II)]^+^ substituents. These compounds were considered to be suitable precursors for our structural aims and thus mark the starting point of our research efforts.^[27]^ In a first step, sodium imidazolide, prepared according to a procedure published by Collmann et al.,^[28]^ was reacted with (η^6^‐chlorobenzene)(η^5^‐cyclopentadienyl)iron(II) hexafluorophosphate (**1**) in a nucleophilic aromatic substitution reaction yielding 73 % of the cationic imidazole **2** (Scheme 1).

**Figure 1:**
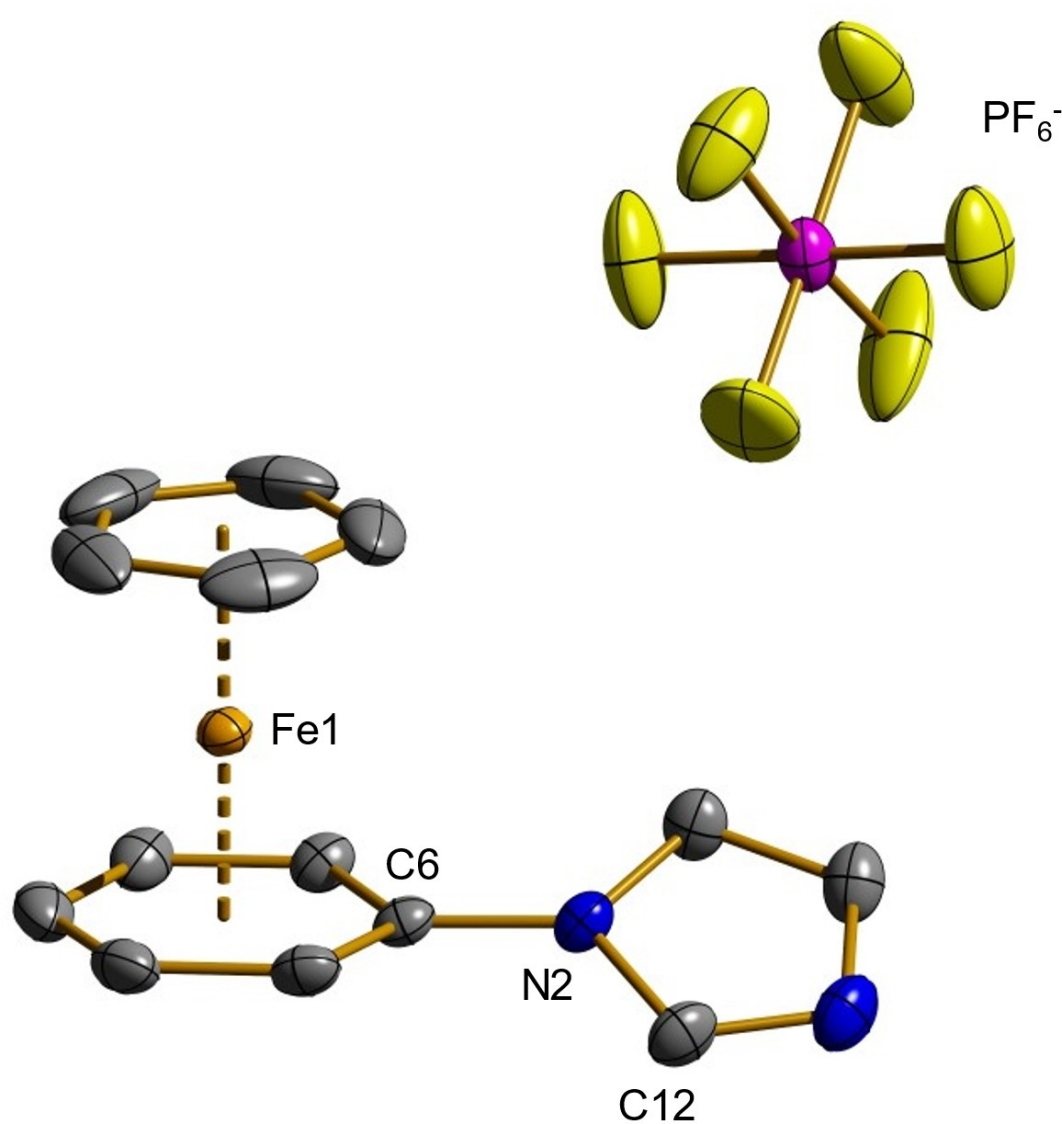
Molecular structure of complex **2** in the solid state. Characteristic bond lengths (Å) and angles (deg), hydrogen atoms are omitted for clarity: Fe1−Cp 1.6627(2), Fe1−Ar 1.5305(2), N2−C6 1.409(2), C6−N2−C12 126.25(16), Cp−Fe−Ar 178.81(2), wherein Cp denotes the center of the η^5^‐coordinated cyclopentadienyl ring. Ar denotes the center of the η^6^‐coordinated arene ring.

^1^H NMR spectroscopy unambiguously proves the successful synthesis of compound **2**. The resonances for the imidazole protons of **2** appear at 8.15, 7.67 and 7.22 ppm, while the signals of the η^6^‐coordinated phenyl ring are observed between 6.71 and 6.28 ppm, thus shifted to higher field compared to a free phenyl group. They exhibit the expected resonances with a doublet at 6.71 ppm for the hydrogen atoms in the *ortho*‐position and two triplets for the hydrogen atoms in the *meta*‐ and *para*‐position at 6.43 and 6.28 ppm, respectively. The protons of the cyclopentadienyl ring are assigned to a singlet at 5.04 ppm. The presence of the hexafluorophosphate anion is proven by ^31^P NMR spectroscopy, which reveals the typical septet for PF_6_^−^ at −144.3 ppm. In addition to the NMR spectra, elemental analysis and ESI mass spectrometry further support the structural identification of **2**. For the cation of complex **2** for example a peak at m/z=265.02 (calcd. 265.04) with a matching isotope pattern was observed. Single crystals of **2** that were suitable for an X‐ray structure analysis were obtained by storing a saturated solution of **2** in acetonitrile at −20 °C for several days. In Figure 1 the molecular structure is depicted and typical bond parameters are summarized.

Cyclopentadienyl and arene rings form a sandwich complex in combination with the iron center, in which the angle between these planes of the five and six‐membered rings was calculated as 1.6°. Thus, these two rings are almost parallel. The imidazole ring is inclined with respect to the phenyl ring by about 28°. Distances between the iron(II) site and the centroids of cyclopentadienyl and arene ring are 1.6627(2) and 1.5305(2) Å, respectively, which reflects the size of the two rings. The newly formed C6−N2 bond in compound **2** has a length of 1.409(2) Å which is slightly shorter than the typical value for a carbon nitrogen single bond.^[29]^ Furthermore, the solid state structure of the corresponding derivative carrying a 4‐methylimidazole substituent instead of an imidazole substituent could also be obtained. It is included in the Supporting Information (Figure S49).

**Scheme 2:**
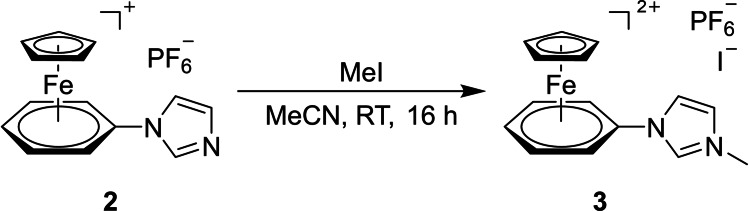
Generation of the dicationic NHC precursor **3**.

To generate a suitable NHC precursor, complex **2** was converted into a dicationic imidazolium salt. Iodomethane turned out to be a suitable alkylating reagent.^[30]^ The reaction of compound **2** with an excess of iodomethane in acetonitrile at room temperature for 16 h provided the 18 VE iron(II) complex **3** in good yields of 71 % (Scheme 2).

**Figure 2:**
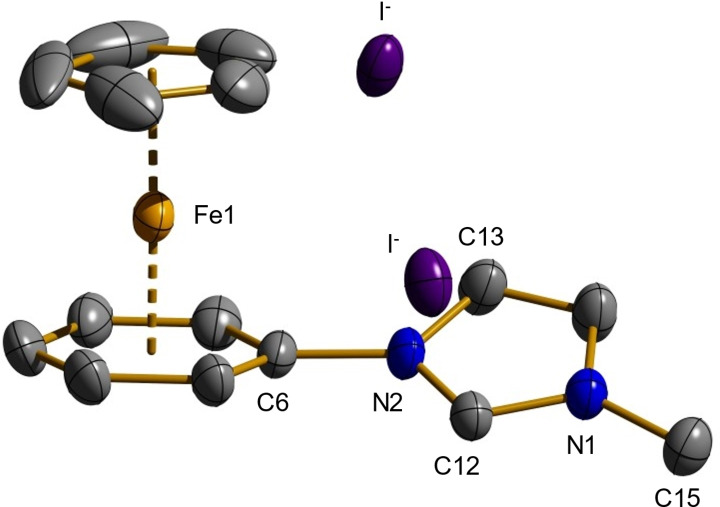
Molecular structure of 3* with two iodide anions in the solid state. Characteristic bond lengths (Å) and angles (deg), hydrogen atoms are omitted for clarity: Fe1−Cp 1.6652(9), Fe1−Ar 1.5359(9), C6−N2 1.432(8), C15−N1 1.473(9), C12−N2−C6 124.4(5), C13−N2−C6 126.8(6). Cp denotes the center of the η^5^‐coordinated cyclopentadienyl ring. Ar denotes the center of the η^6^‐coordinated arene ring.

In both, the ^1^H and the ^13^C NMR spectrum of **3** the additional signal of the methyl group at 4.17 respectively 35.0 ppm is noticeable at first sight. Furthermore, all proton resonances are shifted to lower field compared to compound **1** due to the additional positive charge, which is particularly distinct for the protons of the imidazolium moiety (10.12, 8.30 and 7.73 ppm). The signal exposed at 10.12 ppm can be assigned to the proton between the two nitrogen atoms of the imidazolium group. Its chemical shift indicates a rather high acidity implying a smooth generation of an N‐heterocyclic carbene site in the following step. By ESI‐MS measurements multiple cationic complexes can be identified. Molecular masses of m/z=406.90 and 424.95 correspond to the dicationic ligand scaffold in combination with either an iodide (calcd. 406.97) or a hexafluorophosphate (calcd. 425.03) anion resulting in monocationic species. Interestingly, the cleavage of hydrogen iodide is observed under ESI conditions too, leading to the cationic free carbene with m/z=279.00 (calcd. 279.06). The presumed structure of **3** was finally clarified by X‐ray structure analysis. Single crystals were obtained from a saturated solution of **3** in acetonitrile by slow vaporization of the solvent at room temperature. Figure 2 exhibits the molecular structure of 3* (the asterisk denotes the presence of two iodide anions in the crystal) and summarizes typical bond parameters of the dicationic species which is accompanied by two iodide counter anions.

The molecular structure of compound 3* closely resembles that of compound **2**. The distances between the iron center and the centroids of cyclopentadienyl ring and arene ring are 1.6652(9) and 1.5359(9) Å, respectively, which is within the expected range. In addition, the newly established nitrogen‐carbon bond between N1 and C15 is evident with 1.473(9) Å, which corresponds to a typical carbon‐nitrogen bond. A minimal elongation of the C6−N2 bond (1.432(8) Å) in comparison to the molecular structure of **2** with 1.409(2) Å can be noted. The reason might be the electron‐withdrawing effect of both the cyclopentadienyliron(II) and the imidazolium cations on each side of the bond. Considering the C12−N2‐−C6 angle, only a very small change from 126.25(16)° to 124.4(5)° can be observed.

Additional structural data for the dication of **3** in combination with two hexafluorophosphate counter anions (3**) can be found in the Supporting Information (Figure S50). Although it would have been consistent with the statistical distribution of counterions, the permutation of a single iodide and a hexafluorophosphate was not found up to date in single crystals, which might be explained by a poorer solubility of the homoanionic salts.

**Scheme 3:**
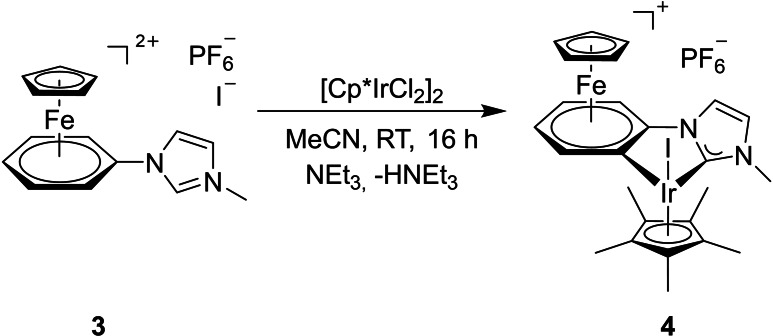
Synthesis of the cyclometalated iridium complex **4**.

With the imidazolium salt in hands, cyclometallation with half an equiv. of the iridium(III) precursor [(η^5^‐C_5_Me_5_)IrCl_2_]_2_ (η^5^‐C_5_Me_5_=1,2,4,5‐pentamethylcyclopentadienyl) was carried out (Scheme 3). According to the presumed high acidity of imidaziolium salt **3**, triethylamine was chosen as the base, which has proven to be well suitable for the synthesis of NHC complexes in combination with rhodium and iridium.^[31]^ The polar solvent acetonitrile ensured sufficient solubility of the dication.

**Figure 3:**
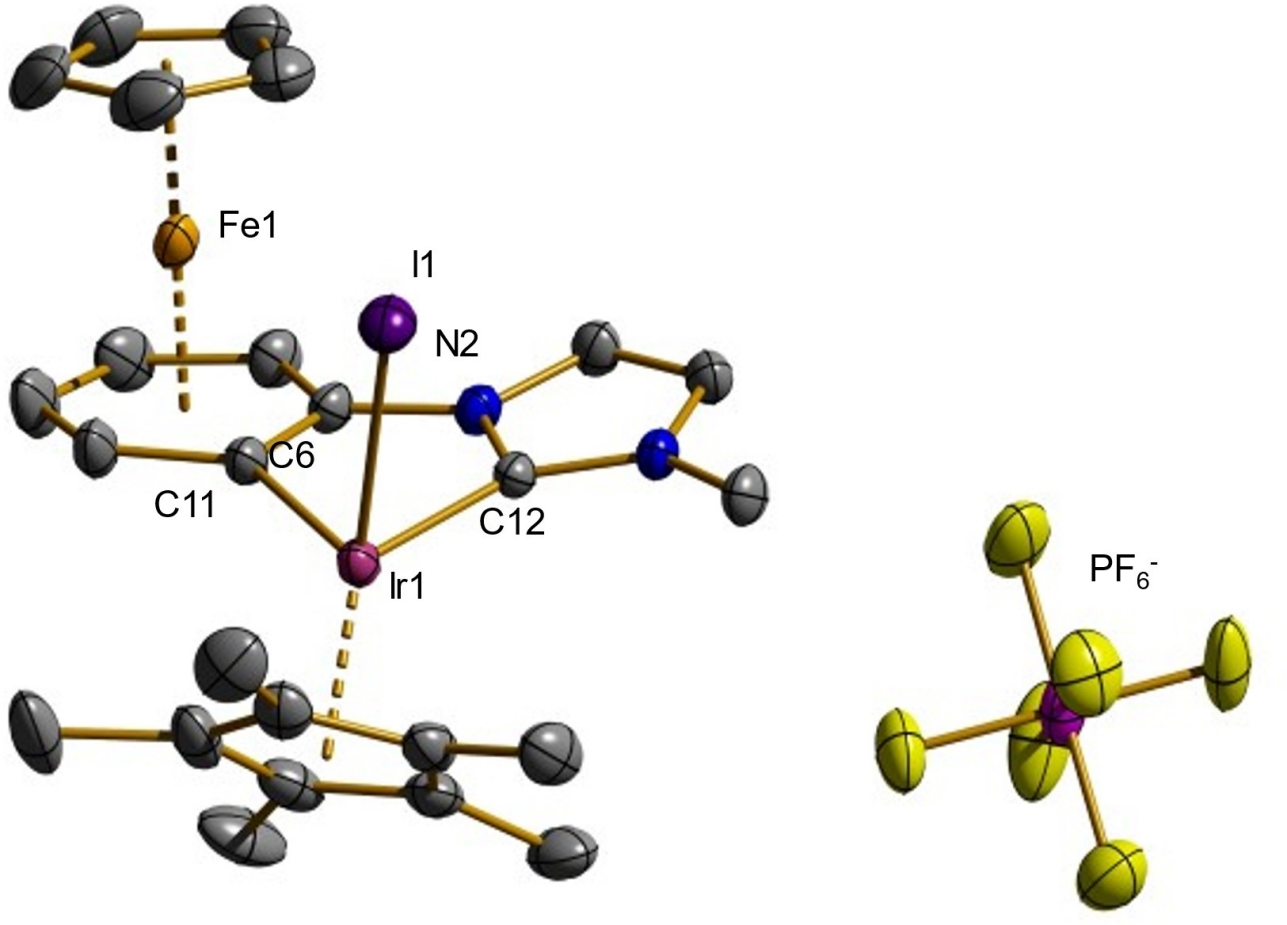
Molecular structure of **4** in the solid state. Characteristic bond lengths (Å) and angles (deg), hydrogen atoms are omitted for clarity: Fe1−Cp 1.6633(5), Fe1−Ar 1.5578(4), N2−C6 1.409(3), Ir1−C11 2.043(2), Ir1−C12 1.999(2), Ir1−I1 2.6882(5), Ir1−Cp* 1.8619(5), C12−Ir1−C11 77.79(8), C12−N2−C6 115.36(2), N2−C6−C11 112.14(2), C12−Ir1−I1 86.55(6), C11−Ir1−I1 97.19(6), C11−C6−N2−C12 6.40(5). Cp denotes the center of the η^5^‐coordinated cyclopentadienyl ring. Ar denotes the center of the η^6^‐coordinated arene ring. Cp* denotes the center of the η^5^‐coordinated 1,2,3,4,5‐pentamethylcyclopentadienyl ring.

The synthesis according to Scheme 3 provided the dinuclear iron‐iridium complex **4** in 85 % yield as an orange‐colored powder in analytically pure form. First evidence for a successful complexation of iridium(III) and the cyclometallation of the iron(II)‐coordinated phenyl ring can be derived from its ^1^H NMR spectrum. One indicator is the absence of the two‐fold nitrogen‐neighbored imidazolium proton. Both the imidazole protons as well as the phenyl protons show a significant shift to higher field compared to the precursor **3** due to the reduced positive charge of the product. The absence of one of the phenyl protons is also conspicuous as a result of the cyclometallation reaction. The signals of the four remaining protons occur as a combination of two doublets and two triplets (ABCD spin system) in the range from 6.55 to 5.86 ppm whereas the resonance of the cyclopentadienyl ring is shifted to 4.89 ppm. In addition to the singlet of the nitrogen‐bound methyl group at 3.88 ppm, an intense singlet at 1.77 ppm is assigned to the 1,2,3,4,5‐pentamethylcyclopentadienyl ligand. The ^13^C NMR spectrum exhibits the familiar signals with the resonance of the carbene carbon atom being located at 163.8 ppm. Additionally, ^31^P NMR data as well as elemental analysis confirm the presence of the hexafluorophosphate counter anion, obviously due to the poorer solubility of triethylammonium chloride compared to triethylammonium hexafluorophosphate. ESI‐MS measurements reveal a peak at m/z=732.93, which is in accordance with the simulation for **4** (733.03). Suitable single crystals for an X‐ray structure analysis were obtained by recrystallization of **4** by keeping a solution of the compound in 1,2‐difluorobenzene overlaid with toluene for several days at −30 °C. Figure 3 depicts the molecular structure of the bimetallic complex in the solid state and summarizes typical bond parameters.

The distances between both the cyclopentadienyl ring and the phenyl ring, and the iron(II) center resemble those of the previous structures, indicating only minor differences in this part of the molecule. However, the C12−N2−C6 angle is decreased from 124.4(5)° to 115.36(2)° due to the chelating coordination of the iridium(III) site. It is remarkable that the bond distance between Ir1 and the carbene‐type carbon atom C12 is significantly shorter (1.999(2) Å) than the bond distance between Ir1 and the carbanion‐type carbon atom C11 (2.043(2) Å). The angles between the arene and the imidazole ring distort slightly from planarity as the torsion angle C12−C6−N2−C11 is 6.4° and angles of 97.19° for C11−Ir1−I1 and 85.66° for C12−Ir1−I1 differ due to nonbonding repulsions in the ligand environment.

### Catalytic transfer hydrogenation

**Scheme 4:**
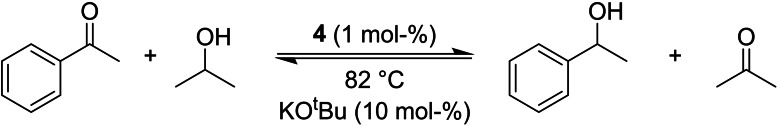
Optimized reaction conditions for the transfer hydrogenation with **4**.

To gain information on the cooperative influence of the [CpFe]^+^ moiety in **4** on the catalytic activity of the iridium(III) site, we examined its performance in the catalytic transfer hydrogenation of ketones and compared the results with those reported by Choudhury et al. for analogue iridium(III) complexes having no [CpFe]^+^ function.^[24]^ As a model reaction the transfer hydrogenation of acetophenone to phenyl ethanol was chosen (Scheme 4).

Since transfer hydrogenation includes the transfer of a metal‐bound hydrido ligand to the carbonyl carbon atom, it might be expected that the chelating and positively charged iridium‐bound NHC ligand of **4** could have a detrimental effect on the catalytic performance. Surprisingly, rather good conversions were observed with a catalyst loading of just 1 mol‐% (Table 1). Different bases were examined. Among them potassium *tert*‐butoxide was found to be the most active, followed by potassium hydroxide and potassium phosphate. 10 mol‐% of base in the reaction mixture gave the best conversions, lower ratios (5 and 7.5 mol‐%) led to an increase of reaction time. Based on our experience with transfer hydrogenation reactions, the temperature was set to 82 °C.^[32]^ The activity of the catalyst decreases rapidly with temperature: At 70 °C, only 4 % of conversion could be detected after 10 min. In the first five minutes the catalyst shows little activity, either due to a warm‐up phase of the reaction mixture or an activation period of the catalyst.

**Table 1:** Substrate scope of the iridium catalyzed transfer hydrogenation.^[a]^

Entry	Substrate	Yield [%]	Time [min]
1	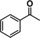	97^[b]^	10
2	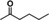	95^[b]^	15
3	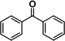	97^[c]^	15
4	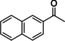	98^[c]^	30
5	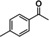	93^[b]^	30
6	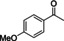	>69^[b]^	30
7	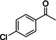	>99^[b]^	60
8	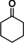	>99^[b]^	90

[a] Reaction conditions: 1 mmol of substrate, 5 mL of isopropanol, 1 mol‐% of catalyst **4**, 10 mol‐% of KO^t^Bu, 100 μL of tetradecane (internal standard), 80 °C, [b] yields determined by GC, [c] yields determined by NMR.

In comparison to the studies of Choudhury et al. employing a related neutral cyclometalated iridium NHC complex without the [CpFe]^+^ complex fragment,^[24]^ the transfer hydrogenation reaction of acetophenone induced by complex **4** is faster by at least two orders of magnitude. While Choudhury et al. observed less than 40 % of racemic 1‐phenylethanol after 3 h using 20 mol‐% of KOH and a reaction temperature of 100 °C, the reaction is completed within minutes in our case. This is even more remarkable since we were able to decrease the reaction temperature to 80 °C and the concentration of the comparable base potassium *tert*‐butoxide to 10 mol‐%. In this case, it should also be explicitly pointed out that we obtained similar results with the base KOH instead of potassium *tert*‐butoxide as well. Thus, the difference in activity is obviously not mainly due to the change of the base. In addition to the model substrate acetophenone, we were able to achieve excellent yields in short reaction times for a series of related substrates (Table 1).

Table 1 reveals that beside acetophenone, the sterically more demanding substrates 2‐acetonaphthone and benzophenone can be converted at similar rates. Various *para*‐substituted acetophenone derivatives (entries 5 to 7) also provide rapid conversions to the corresponding alcohol in equilibrium regardless of the nature of the substituent. Moreover, even electron‐rich aliphatic ketones such as 2‐hexanone and cyclohexanone can be hydrogenated efficiently. In contrast, the transfer hydrogenation of benzaldehyde required 24 h for a conversion of just 48 % and the reduction of nitrobenzene to aniline (or intermediates) was not observed within one day.

### Computational study

The generally high activity of the cationic catalyst **4** in transfer hydrogenation reactions raises the question for the mechanism.

**Scheme 5:**
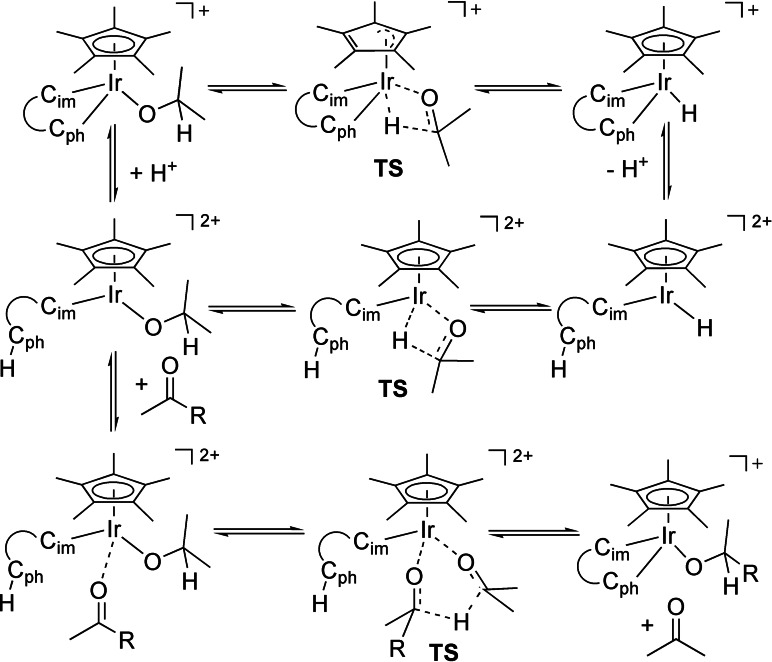
Possible mechanisms for the transfer hydrogenation with compound **4** (the C,C chelating ligand is abbreviated by its both carbon donor sites): top, β‐hydrogen transfer reaction with preservation of the C,C coordination; middle, β‐hydrogen transfer reaction after protolytic cleavage of the Ir‐phenyl bond; bottom: Meerwein‐Pondorf‐Verley‐type mechanism.

According to the transfer hydrogenation process depicted in Scheme 4, dihydrogen is transferred from the hydrogen donor isopropanol to the aromatic ketone. While the oxygen‐bound hydrogen atom can be transferred via a simple proton exchange between an acid and a base, there are at least three possible mechanisms for the carbon‐bound hydrogen atom to be transferred, which are summarized in Scheme 5.

On one side, a β‐hydrogen transfer from an iridium‐coordinated isopropanolato ligand can take place. The ensuing acetone ligand is then substituted in equilibrium by the aromatic ketone, to which the hydrido ligand is transferred. Alternatively, the β‐hydrogen atom is directly transferred from the iridium‐coordinated isopropanolate to the carbonyl carbon atom of an iridium‐coordinated ketone (inner or outer sphere).^[33]^

**Figure 4:**
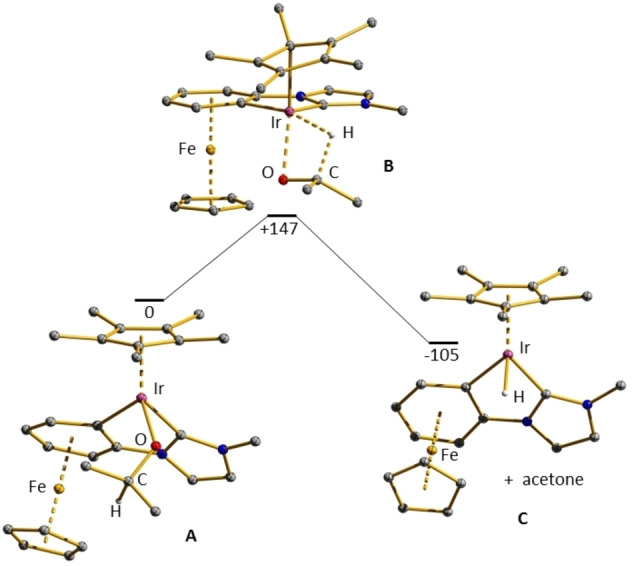
Sequence A‐B‐C.

Therefore, in the DFT‐calculated sequences, the isopropanolato complex **A** has to be generated for both cases in the first step, starting from the monocationic 18 VE complex **4** by substitution of the iodido ligand by isopropanolate.^[34]^ The structure of complex **A** is depicted in Figure 4.

Geometry optimization of the isopropanolato complex **A** revealed the geometric parameters listed in Table S2. An Ir−H distance of 331 pm and an Ir−O−C angle of 126° indicate that the β‐hydrogen atom of the isopropanolate is not bonded to the iridium(III) center. The saturated 18 VE situation of the iridium(III) center and the nonbonding repulsion of the isopropyl CH_3_ groups with the other ligands around appear to be the cause. To calculate the reaction pathway of a β‐hydrogen transfer to the metal center we elongated the C−H_β_ bond and optimized the geometries along the path. The transition state **B** was located ΔE^#^=+147 kJ/mol above complex **A**. In transition state **B**, a short distance Ir−C of 2.17 Å and an angle of Ir−C−C of 119° are indicative of a η^1^‐bonded pentamethylcyclopentadienyl ligand. By the shift in bonding from η^5^ to η^1^, the formally 18 valence electron iridium complex generates a vacant coordination site at the metal center in the transition state **B**. IRC calculations revealed the reaction path depicted in Figure 4 including a ring slippage from η^5^ to η^1^ of the cyclopentadienyl ligand. Geometry optimization of the intermediate **C** revealed a short Ir−H distance of 1.60 Å and Ir−C11 and Ir−C12 distances of 2.04 respectively 2.01 Å as well as similar Ir−C distances for the pentamethylcyclopentadienyl ring. These parameters are indicative of a nearly tetrahedral 18 VE iridium complex in the final state. In addition, the resulting ketone is not bound to the iridium center. We found that the ketone is bound via a weak O−H hydrogen bond to the methylimidazolylidene ring. The energy of complex **C** was calculated to be 105 kJ/mol below complex **A**.

The catalytic transfer hydrogenation (Table 1, Entry 1) was performed at 80 °C and is completed after 10 minutes employing acetophenone, indicating a much lower activation barrier. Thus, the β‐hydrogen transfer to the saturated iridium center is not very likely. In addition, the strong stabilization of intermediate **C** will result in a very high activation barrier for the reverse reaction, a migratory insertion of a ketone into the Ir−H bond, here ca. 260 kJ/mol.

**Figure 5:**
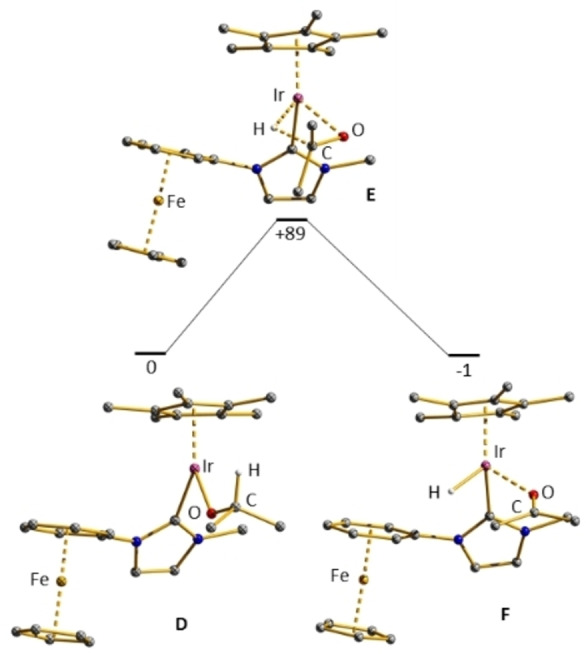
Optimized structures along the beta‐hydrogen transfer paths **D‐E‐F**. For this path, a proton has been transferred to the phenyl ring.

From our calculations we conclude that an additional coordination site at an unsaturated iridium center is deemed necessary. Under the reaction conditions with an excess of isopropanol, a proton transfer might occur to the CpFe(phenylimidazolylidene) ligand generating a free coordination site accompanied with a protonated ligand as depicted in Figure 5 (structure **D**). To decide, whether such a reversible protic ligand cleavage is possible in isopropanol, compound **4** was stirred with 10 mol‐% of KO^t^Bu in isopropanol‐d^8^ for 1 h at 80 °C. The reversibility of the cyclometallation reaction was detected by means of ^2^D NMR spectroscopy, which provided one resonance at 5.69 ppm, that is assigned to the deuteration of the *ortho*‐position of the iron(II) coordinated phenyl ring. From this observation it not only can be concluded, that the cyclometallation is reversible in isopropanol (in the presence of a base). It can also be concluded that there must be a second, minor isomer of compound **4**, wherein the (CpFe) and the (Cp*Ir) units are not oriented in anti‐ but in syn‐position to each other. Calculation of complex **A‐syn** revealed nearly the same energy than for complex **A‐anti** which is in accord with the experimental observation.

To reveal the activation barriers for the β‐hydrogen transfer from the isopropanolato ligand to the iridium center, we calculated the reaction path, which is presented in Figure 5. A transition state **E** was located 89 kJ/mol above **D**, which is about 54 kJ/mol lower in relative energy than transition state **B** for the saturated 18 VE complex (Figure 4). Thus, the β‐H‐transfer to the iridium center is orders of magnitude slower in the 18 VE system. To complete the catalytic cycle, the acetone in complex **F** will be replaced by the substrate ketone following back the reaction path from **F** to **D**. The alcoholato ligand is then replaced by isopropanol revealing an isopropanolato ligand and the finally hydrogenated alcohol.

For a related path starting from a 16 VE iridium complex with a protonated imidazolidine ligand we calculated an activation barrier of 109 kJ/mol (transition state E′, see the Supporting Information Figure S51), which is about 20 kJ/mol higher than the barrier shown in Figure 4 (protonated phenyl ring). Thus, this path was excluded.

An alternative path includes the direct outer‐sphere hydride transfer to a ketone as suggested in a Meerwein‐Ponndorf‐Verley (MPV) type of reaction or to an outer‐sphere assembled ketone in the reaction mixture. Due to our experimental results that the hydrogenation of substrate **7** is slower by a factor of about six, an outer‐sphere hydride transfer seems unlikely.^[24]^

**Figure 6:**
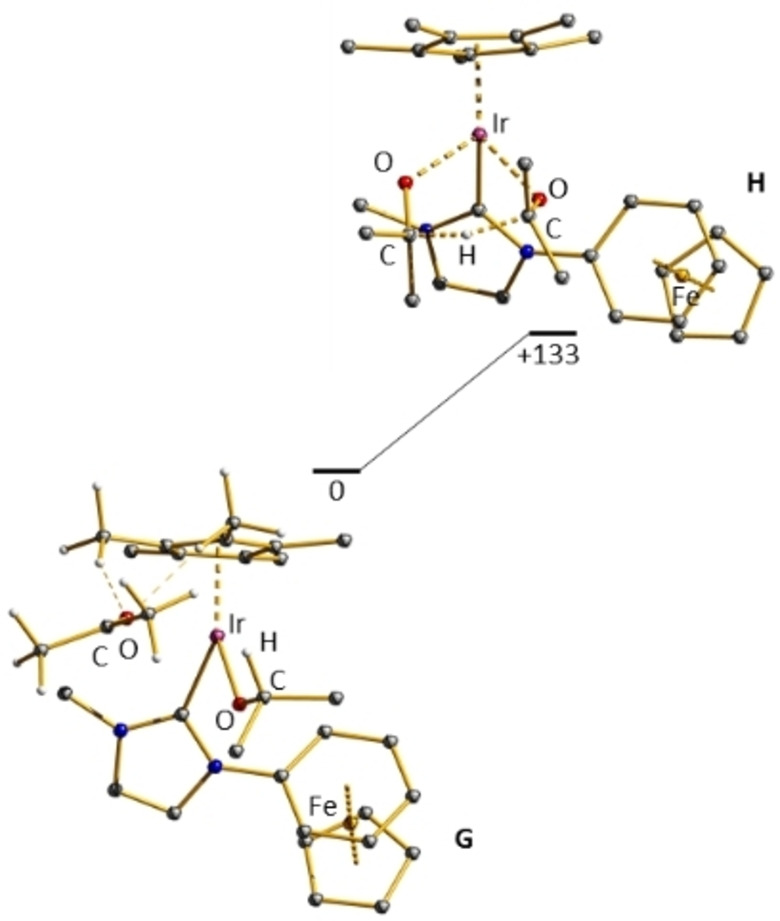
Optimized structures along the reaction path **G‐H** of the MPV‐type reaction sequence.

For six, the calculation of a MPV reaction path of the isenthalpic hydrogen transfer from isopropanol to acetone we started from the dicationic complex **G**, which was generated by addition of an acetone ligand to complex **D** depicted in Figure 6. A short bond Ir−O1 of 1.94 Å and a very long Ir−O2 distance of 4.41 Å indicate, that in the initial complex **G** the ketone is only weakly hydrogen bonded to the complex as depicted in Figure 6. The energy for complex **G** was calculated 31 kJ/mol below complex **D**. Following the hydrogen transfer path from the alcoholato to the ketone ligand, a further a transition state **H** was reached at β‐C−H distances ligand, of 1.31 Å and 1.34 Å respectively as depicted in Figure 6. Distances Ir−O1 of 2.16 Å and Ir−O2 of 2.15 Å indicate a six membered ring consisting of two carbonyl groups one hydrogen and one iridium center in a twist‐like conformation (cf. Figure 6). The energy for transition state **H** is calculated 133 kJ/mol above complex **G**. Transition state **H** is calculated to be 13 kJ/mol less favored than transition state **E** starting from complex **G** upon dissociation of acetone. Thus, an outer‐sphere hydride transfer from the alcoholate to an acetone molecule is calculated to be much slower than by a sequence of β‐hydride transfer to the iridium center, exchange of the ketone and reinsertion of the ketone into the Ir−H bond.

We conclude from our DFT‐calculations, that a transfer hydrogenation proceeding via a 18 VE species with a carbanion and NHC coordinating (chelating) ligand is much slower than the same reaction occurring with a complex, where the Ir−C(phenyl) bond is initially cleaved by proton transfer. The presence of the [CpFe]^+^ fragment resulting in a weakened Ir−C(phenyl) bond appears to be the cause for this reaction path. We also observe a decrease in the reactivity of catalyst **4** by switching from acetophenone to 4‐Cl‐acetophenone as the substrate in contrast to the observations of Choudhury et al.

## Conclusion

A suitable route for the preparation of a dicationic imidazolium compound carrying a (η^5^‐cyclopentadienyl)(η^6^‐phenyl)iron(II) substituent was established. Both, the mono cationic imidazole precursor and the dicationic imidazolium salt were fully characterized and crystal structures could be obtained. Furthermore, a monocationic, cyclometalated iridium(III)/iron(II) complex was prepared from the imidazolium salt. This bimetallic iridium(III)/iron(II) compound provides unexpectedly high activity in transfer hydrogenation reactions. Especially in comparison with the neutral cyclometalated iridium(III) complex derived from 3‐methyl‐1‐phenyl‐1*H*‐imidazol‐3‐iumiodide the increase in catalytic activity is substantial. The cationic cyclopentadienyl iron complexation appears to be the cause. The new bimetallic catalyst allows a reduced amount of base as well as moderate temperatures. DFT‐calculations on the reaction path revealed, that a reaction sequence starting from a β‐H‐transfer to the iridium center, replacement of the resulting ketone and reinsertion into the Ir−H bond is energetically favored over an outer‐sphere hydride transfer along a Meerwein‐Pondorf‐Verley type of path starting from a 16 valence electron complex. However, further investigations of the reaction mechanism are mandatory. In particular, we are interested in whether the increased catalytic activity is due to the positive charge of the molecule or due to a direct influence of the iron center. Moreover, the application of the catalyst in tandem reactions seems feasible.

## Experimental Section

**(η^6^‐Chlorobenzene)(η^5^‐cyclopentadienyl)iron(II) hexafluorophosphate (1)**: Compound **1** was prepared following a procedure published in the literature.^[27b]^ All reactions were carried out under an argon atmosphere using standard Schlenk techniques. The solvents were either freshly distilled or dried and degassed before use according to standard techniques. Commercially available chemicals were purchased from ABCR, Alfa Aesar, Sigma Aldrich, Strem or TCI. ^1^H, ^13^C and ^31^P NMR spectra were recorded on BRUKER Spectrospin Avance 400 and 600 spectrometers. The chemical shifts are referenced to internal solvent resonances. The multiplicities are reported as s=singlet, d=doublet, t=triplet, q=quartet, sept=septet and m=multiplet. ESI‐mass spectrometric measurements were performed on an AmaZon ETD by introducing solutions of the compound in acetonitrile. Elemental analyses were carried out with a Vario MICRO Cube elemental analyzer at the Analytical Laboratory of the Technische Universität Kaiserslautern. For GC analyses, a Clarus 580 GC equipped with an FID‐detector was used.

**(η^5^‐Cyclopentadienyl)(η^6^‐1‐phenyl‐1*H*‐imidazol)iron(II) hexafluorophosphate (2)**: 4.96 g (13.1 mmol) of (η^6^‐chlorobenzene)(η^5^‐cyclopentadienyl)iron(II) hexafluorophosphate (**1**) and 2.03 g (22.5 mmol) of freshly prepared sodium imidazolide were stirred in 50 mL of acetonitrile at room temperature for 2 h. The reaction mixture was filtered over neutral alumina and the solvent was removed under vacuum. The product precipitated as a yellow powder after the addition of 25 mL of diethyl ether. Yield: 3.92 g (73 %). Anal. calcd. for C_14_H_13_F_6_FeN_2_P: C 41.01, H 3.20, N 6.83; found: C 40.50, H 2.91, N 6.82 %. ^1^H NMR (400 MHz, CD_3_CN): δ 8.15 (s, 1H), 7.67 (s, 1H), 7.22 (s, 1H), 6.71 (d, *J*=6.7 Hz, 2H), 6.43 (t, *J*=6.5 Hz, 2H), 6.28 (t, *J*=6.2 Hz, 1H), 5.04 (s, 5H). ^13^C NMR (101 MHz, (CD_3_)_2_CO): δ 137.4, 132.6, 119.0, 108.0, 88.6, 88.2, 81.1, 79.2. ^31^P NMR (162 MHz, CD_3_CN): δ −144.3 (sept, *J*=711.3 Hz). ESI‐MS (CH_3_CN): m/z 265.02 [C_14_H_13_FeN_2_]^+^.

**(η^5^‐Cyclopentadienyl)(η^6^‐1‐phenyl‐4‐methyl‐1*H*‐imidazol)iron(II) hexafluorophosphate (2 a)**: This complex was synthesized as described above for compound **2** using 0.14 g (1.32 mmol) of freshly prepared sodium 4‐methylimidazolide and 0.50 g (1.32 mmol) of **1**. Yield: 390 mg (70 %) of a yellow solid. Anal. calcd. for C_15_H_15_F_6_FeN_2_P C 42.48, H, 3.56 N, 6.61; found: C 42.11, H 3.45, N 6.49 %. ^1^H NMR (600 MHz, CD_3_CN): δ 8.01 (s, 1H), 7.38 (s, 1H), 6.65 (d, J=6.4 Hz, 2H), 6.40 (t, J=6.4 Hz, 2H), 6.25 (t, J=6.1 Hz, 1H), 5.01 (s, 5H), 2.23 (s, 3H). ^13^C NMR (151 MHz, CD_3_CN): δ 142.1, 136.8, 118.4, 115.3, 108.0, 88.3, 87.9, 80.5, 79.0, 13.9. ^31^P NMR (162 MHz, CD_3_CN): δ −144.6 (sept, *J*=706.2 Hz). ESI‐MS (CH_3_CN): m/z 279.00 [C_15_H_15_FeN_2_]^+^.

**(η^5^‐Cyclopentadienyl)(η^6^‐1‐phenyl‐3‐methylimidazolium)iron(II) hexafluorophosphate iodide (3)**: 6.00 g (14.6 mmol) of compound **2** were dissolved in 25 mL of acetonitrile. 22.8 g (159 mmol, 10 mL) of iodomethane were added. After stirring overnight at room temperature, a saffron‐yellow solid had precipitated. It was washed with diethyl ether (3x10 mL) and isolated with a yield of 71 % (5.70 g, 10.3 mmol). Anal. calcd. for C_15_H_16_F_6_FeIN_2_P: C 32.64, H 2.92, N 5.07; found: C 32.91, H 2.97, N 5.16 %. ^1^H NMR (600 MHz, CD_3_NO_2_): δ 10.12 (s, 1H), 8.30 (s, 1H), 7.73 (s, 1H), 7.29 (d, *J*=6.5 Hz, 2H), 6.75 (t, *J*=6.4 Hz, 2H), 6.62 (d, *J*=6.2 Hz, 1H), 5.41 (s, 5H), 4.17 (s, 3H). ^13^C NMR (151 MHz, CD_3_NO_2_): δ 138.5, 127.0, 123.0, 105.7, 90.2, 89.2, 83.8, 80.9, 38.2. ^31^P NMR (162 MHz, CD_3_NO_2_): δ −142.4 (sept, *J*=711.3 Hz). ESI‐MS (CH_3_CN): m/z 424.95 [C_15_H_16_FeN_2_PF_6_]^+^, 406.90 [C_15_H_16_FeN_2_I]^+^, 279.00 [C_15_H_15_FeN_2_]^+^.

**[(η^5^‐Cyclopentadienyl)κ^2^C^2,7^‐(η^6^‐1‐phenyl‐3‐methylimidazol‐2‐yliden)iron(II)](iodido(η^5^‐1,2,3,4,5‐pentamethylcyclopentadienyl)iridium(III)) hexafluorophosphate (4)**: 410 mg (0.52 mmol) of bis(dichlorido(η^5^‐1,2,3,4,5‐pentamethylcyclopentadienyl)iridium(III)) and 569 mg (1.03 mmol) of compound **3** were stirred at room temperature for 16 h in 25 mL of acetonitrile in the presence of 1.13 g (11.2 mmol, 1.55 mL) of triethylamine. The solvent was removed under vacuum and the crude product was resolved in 20 mL of dichloromethane. After extraction, drying over magnesium sulfate and removing of the solvent, **3** was obtained with a yield of 85 % (764 mg, 0,87 mmol) as an orange‐colored powder. Anal. calcd. for C_25_H_29_F_6_FeIIrN_2_P: C 34.22, H 3.33, N 3.19; found: C 34.11, H 3.47, N 3.14 %. ^1^H NMR (400 MHz, CD_3_CN): δ 7.75 (d, *J*=2.2 Hz, 1H), 7.40 (d, *J*=2.2 Hz, 1H), 6.55 (d, *J*=6.1 Hz, 1H), 6.51 (d, *J*=6.0 Hz, 1H), 5.92 (t, *J*=5.9 Hz, 1H), 5.86 (t, *J*=6.0 Hz, 1H), 4.89 (s, 5H), 3.88 (s, 3H), 1.77 (s, 15H). ^13^C NMR (151 MHz, CD_3_CN) δ 163.7, 125.9, 116.9, 115.4, 113.1, 94.6, 93.8, 84.4, 81.2, 75.9, 71.4, 38.5, 10.0. ^31^P NMR (162 MHz, CD_3_CN) δ −144.62 (sept, *J*=706.6 Hz). ESI‐MS (CH_3_CN): m/z 732.93 [C_25_H_29_FeIIrN_2_]^+^.

**General procedure for the catalytic transfer hydrogenation**: 8.8 mg (0.01 mmol) of catalyst **4**, 11.8 mg (0.1 mmol) of potassium *tert*‐butoxide and 76.7 mg (0.38 mmol, 100 μL) of the internal standard tetradecane were dissolved in 5 mL of isopropyl alcohol in a crimp‐cap vial. Then the ketone was added, the vial was closed and the mixture was heated to 80 °C. Samples were taken at defined times with single‐use syringes, filtered through a short column filled with a small amount of neutral alumina and MgSO_4_, eluted with ethyl acetate, and analyzed by gas chromatography.

**Computational details**: All calculations were performed using the Gaussian16 suit of programs [ref gaussian16].^[35]^ For all atoms the DEF2‐TZVP basis set was employed.^[36]^ For corrections of exchange and correlation we employed the B3LYP three parameter functional of Becke.^[37]^ All stationary points were checked by frequency calculations revealing no imaginary frequency for initial and final state and only one for the transition states. Reaction paths were followed by IRC calculations connecting the minima with the transition state.^[38]^

**X‐ray structure analyses**: Crystal data and refinement parameters are collected in the Supporting Information (Table S1). All structures were solved using direct method, SIR2011 for **2**,^[39]^ SIR92 for **2 a** and **3 a**,^[40]^ SHELXS for **3**,^[41]^ and SHELXT for **4**,^[42]^ completed by subsequent difference Fourier syntheses, and refined by full‐matrix least‐squares procedures.^[43]^ Analytical numeric absorption correction was carried out to complexes **2** and **3 a**,^[43]^ numerical absorption correction based on gaussian integration was applied to complex **3**,^[43]^ and for complex **2 a** ^[43]^ and **4** ^[44]^ semi‐empirical absorption correction from equivalents were used. All non‐hydrogen atoms were refined with anisotropic displacement parameters. All hydrogen atoms were placed in calculated positions and refined by using a riding model. Deposition Numbers 2092872 (for **2**), 2092873 (for **2a**), 2092874 (for 3*), 2092875 (for 3**) and 2092876 (for **4**) contain the supplementary crystallographic data for this paper. These data are provided free of charge by the joint Cambridge Crystallographic Data Centre and Fachinformationszentrum Karlsruhe Access Structures service.

## Conflict of interest

The authors declare no conflict of interest.

## Supporting information

As a service to our authors and readers, this journal provides supporting information supplied by the authors. Such materials are peer reviewed and may be re‐organized for online delivery, but are not copy‐edited or typeset. Technical support issues arising from supporting information (other than missing files) should be addressed to the authors.

Supporting Information
