# Supplementary material for: A Cyclometalated NHC Iridium Complex Bearing a Cationic (η5‐Cyclopentadienyl)(η6‐phenyl)iron Backbone
Source: Chemistry. 2021 Oct 5;27(61):15209–17. doi: 10.1002/chem.202102520 (PMC8596692; doi:10.1002/chem.202102520)
Supplement: Supplementary file 1 — Supporting Information [file CHEM-27-15209-s001.pdf]

Chemistry–A European Journal

Supporting Information

A Cyclometalated NHC Iridium Complex Bearing a Cationic (η^5-Cyclopentadienyl)(η^6-phenyl)iron Backbone

Chemistry–A European Journal

Supporting Information

A Cyclometalated NHC Iridium Complex Bearing a Cationic (η^5-Cyclopentadienyl)(η^6-phenyl)iron Backbone

Content

1. Additional Spectra	1 - 10
2. Catalytic Studies	11 - 54
3. Crystallographic Data	55 - 57
4. Computational Study	58
5. Deuteration experiment	59

1. Additional Spectra

(η^5-Cyclopentadienyl)(η^6-1-phenyl-1*H*-imidazol)iron(II) Hexafluorophosphate (2)

Figure S1: ^{1}H NMR spectrum of **2.**

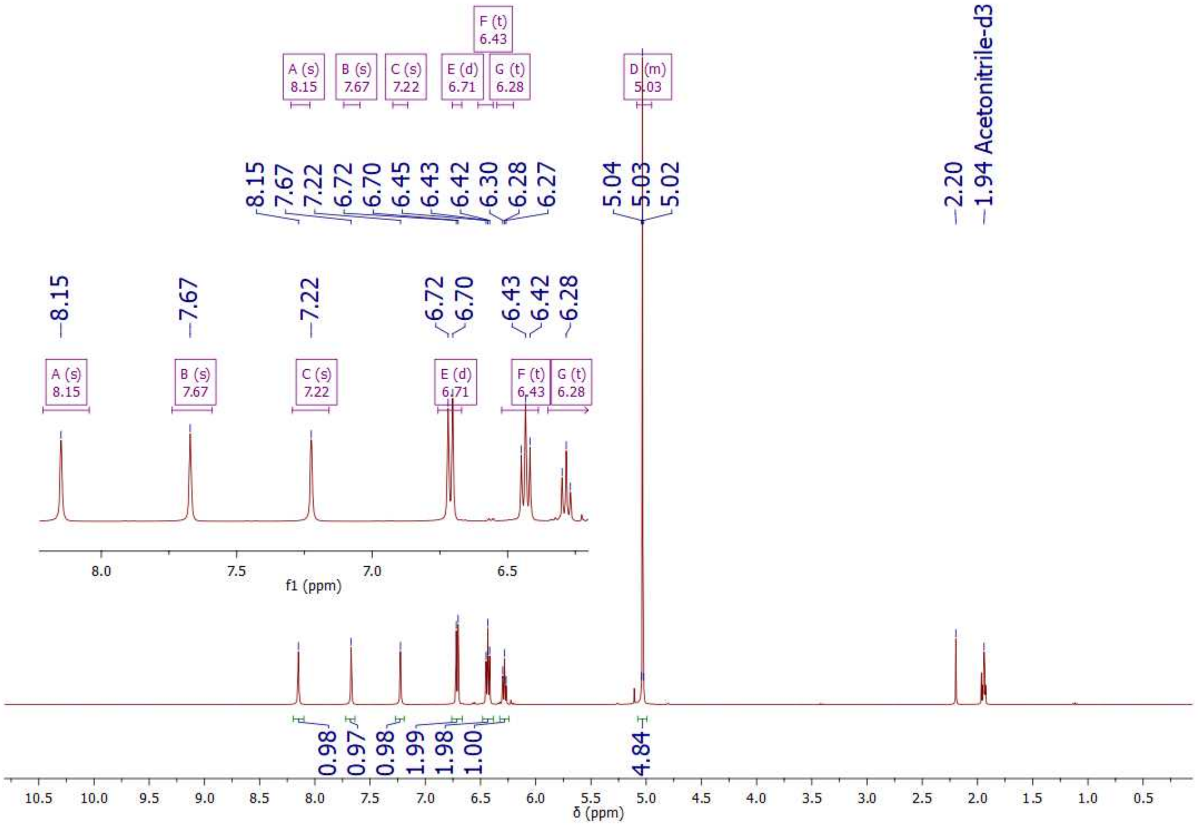

Figure S2: ^{13}C NMR spectrum of **2**.

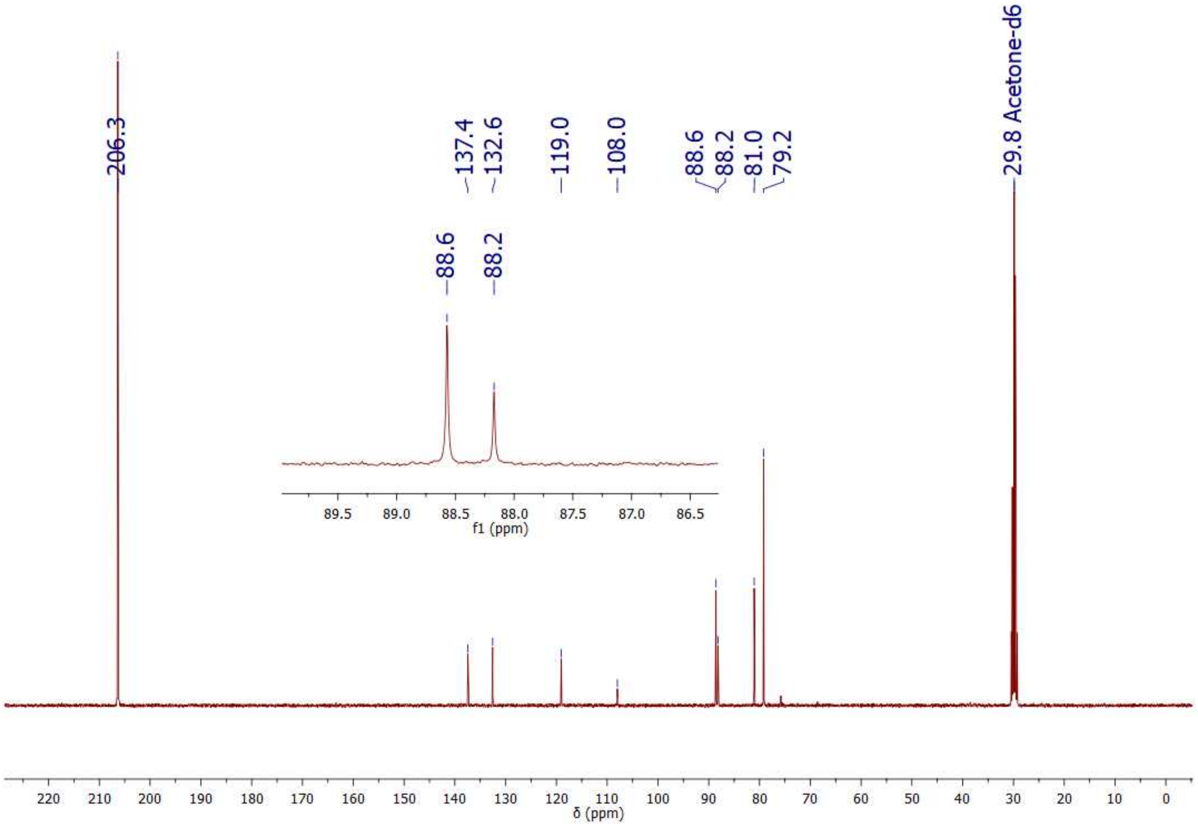

Figure S3: ^{31}P NMR spectrum of **2**.

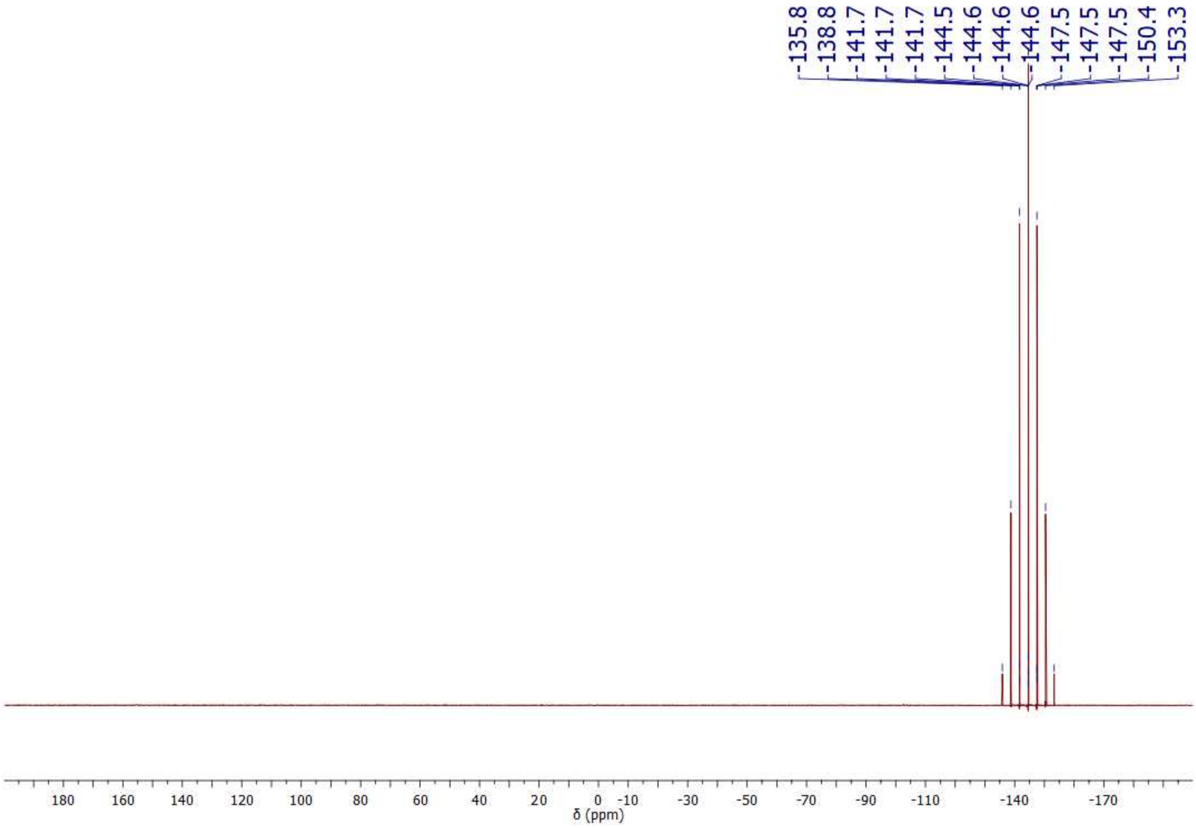

Figure S4: ESI-MS overview spectrum of **2**.

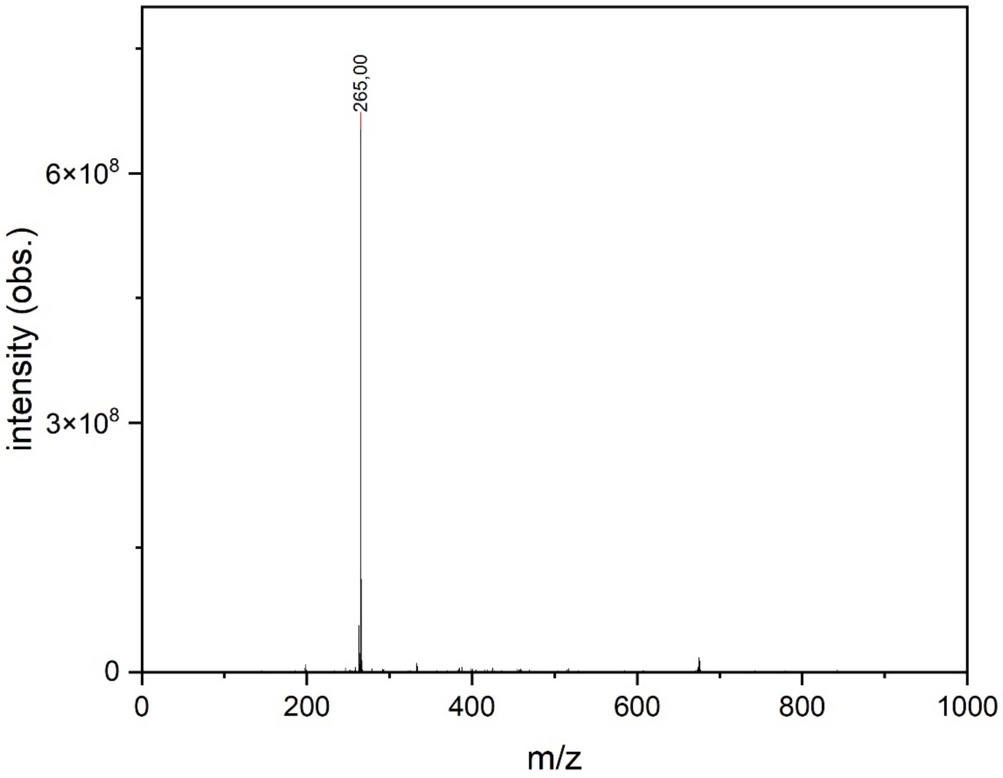

(η5-Cyclopentadienyl)(η6-1-phenyl-4-methyl-1H-imidazol)iron(II) Hexafluorophosphate (2a)

Figure S5: ^{1}H NMR spectrum of **2a**.

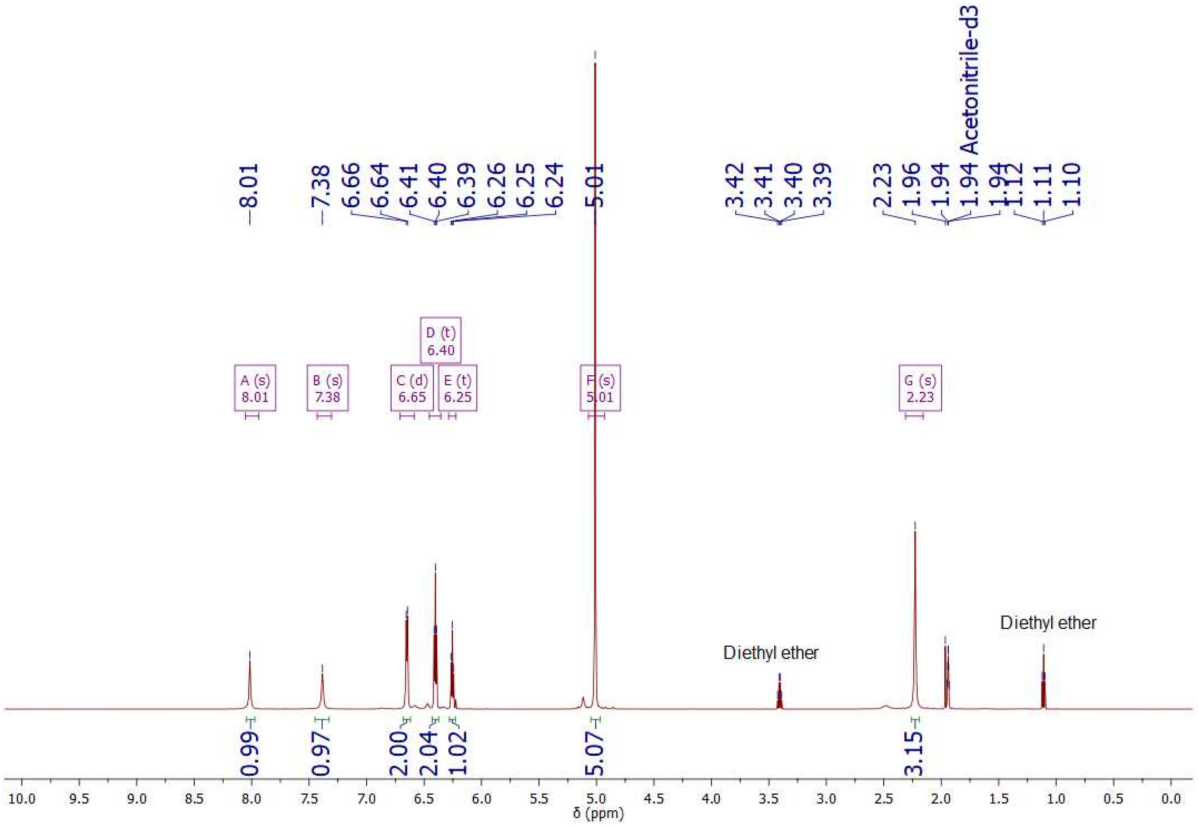

Figure S6: ^{13}C NMR spectrum of **2a**.

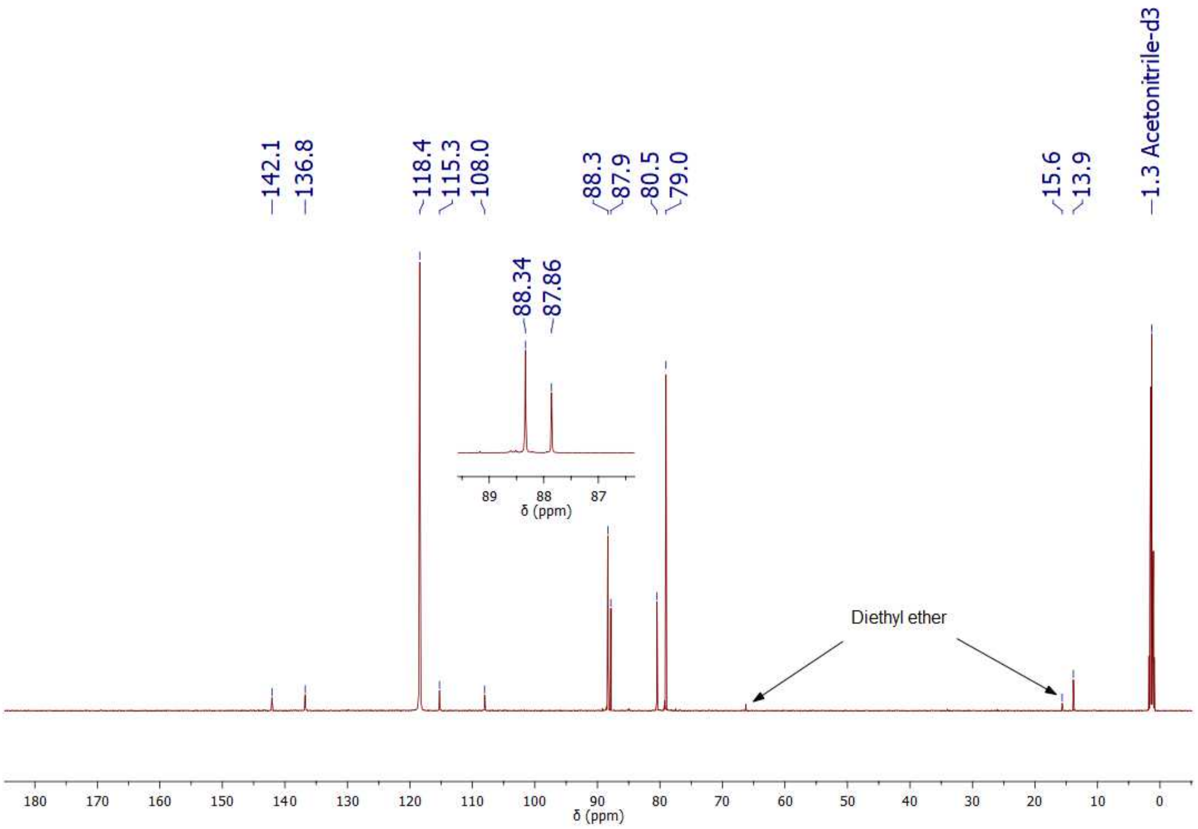

Figure S7: ^{31}P NMR spectrum of **2a**.

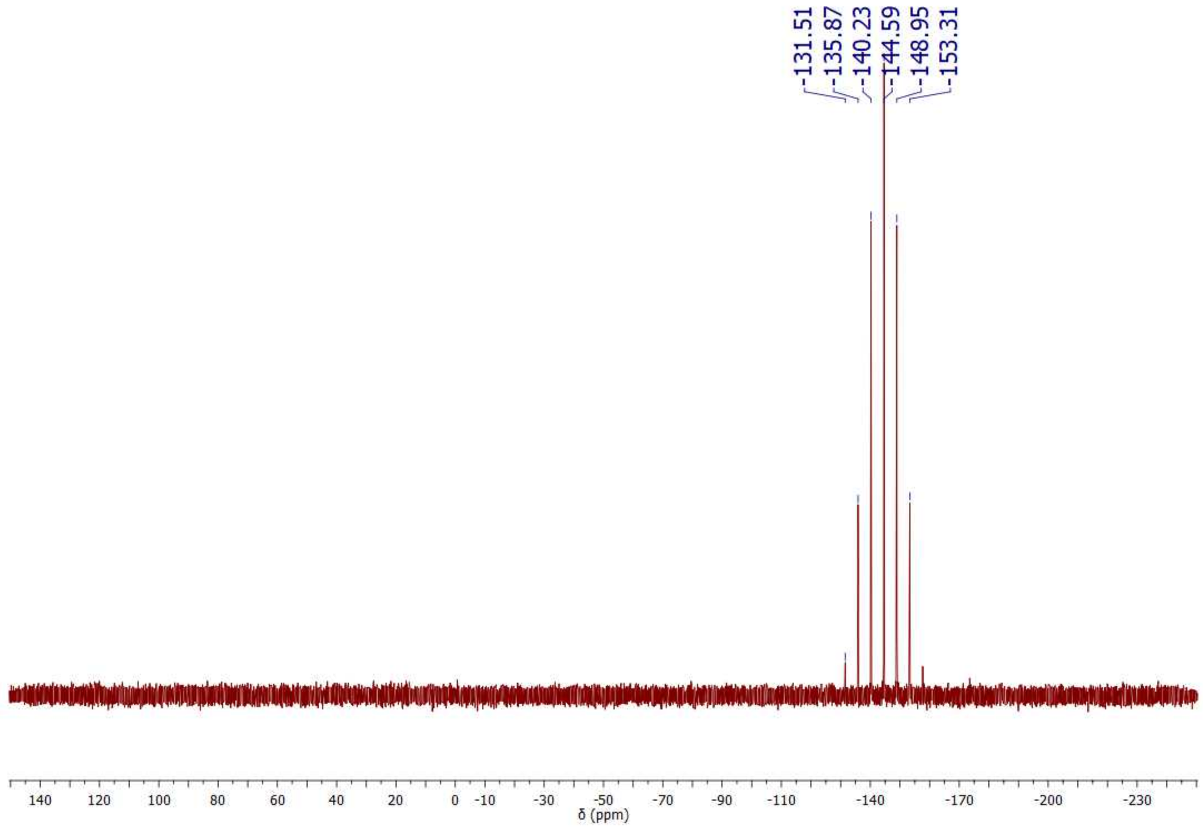

Figure S8: ESI-MS overview spectrum of **2a**.

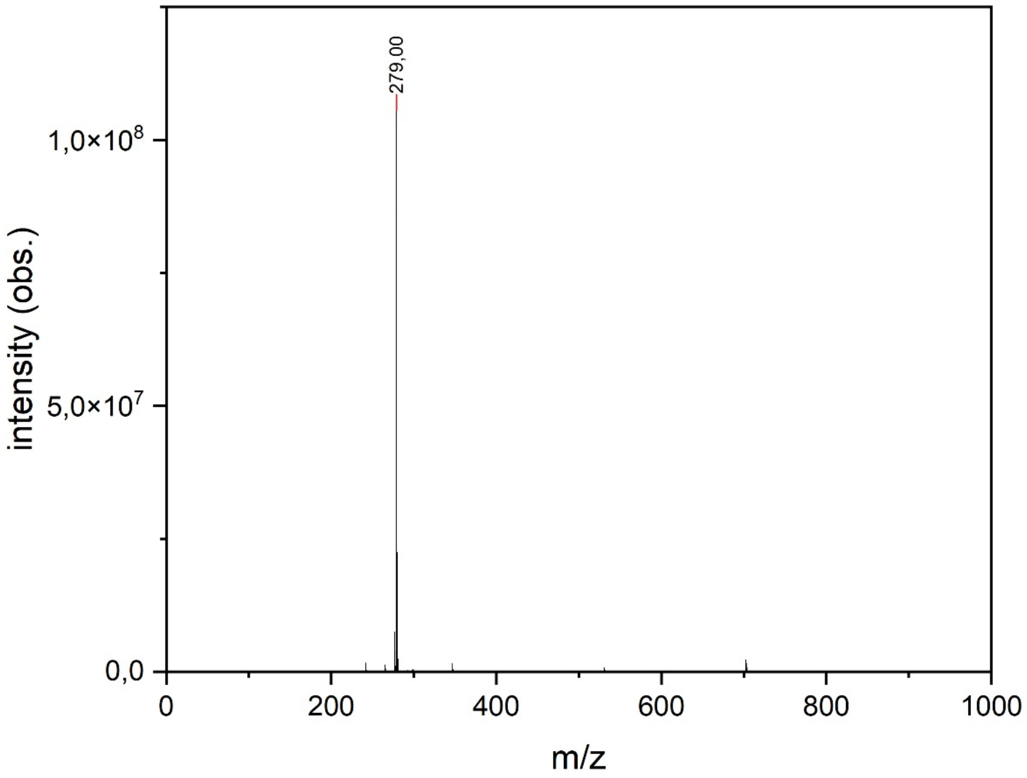

(η[5]-Cyclopentadienyl)(η[6]-1-phenyl-3-methylimidazolium)iron(II) Hexafluorophosphate Iodide (3)

Figure S9: ^{1}H NMR spectrum of **3**.

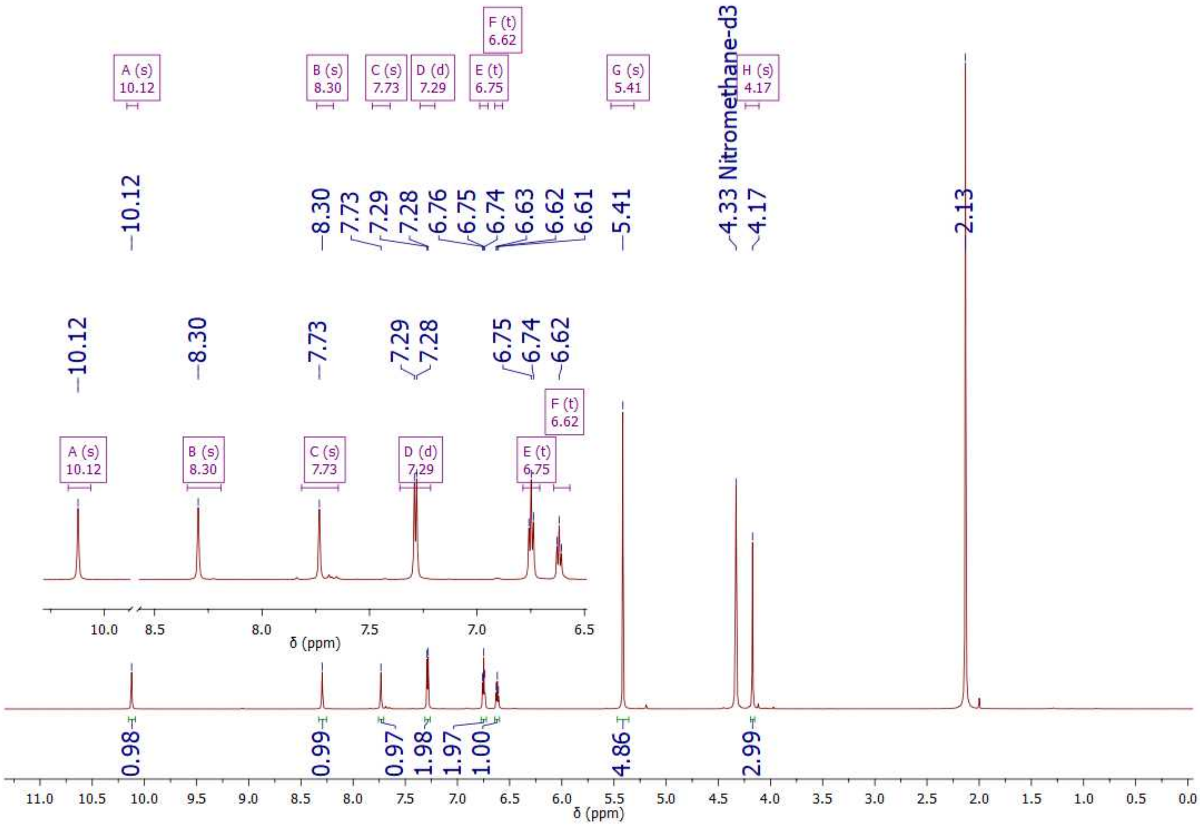

Figure S10: ^{13}C NMR spectrum of **3**.

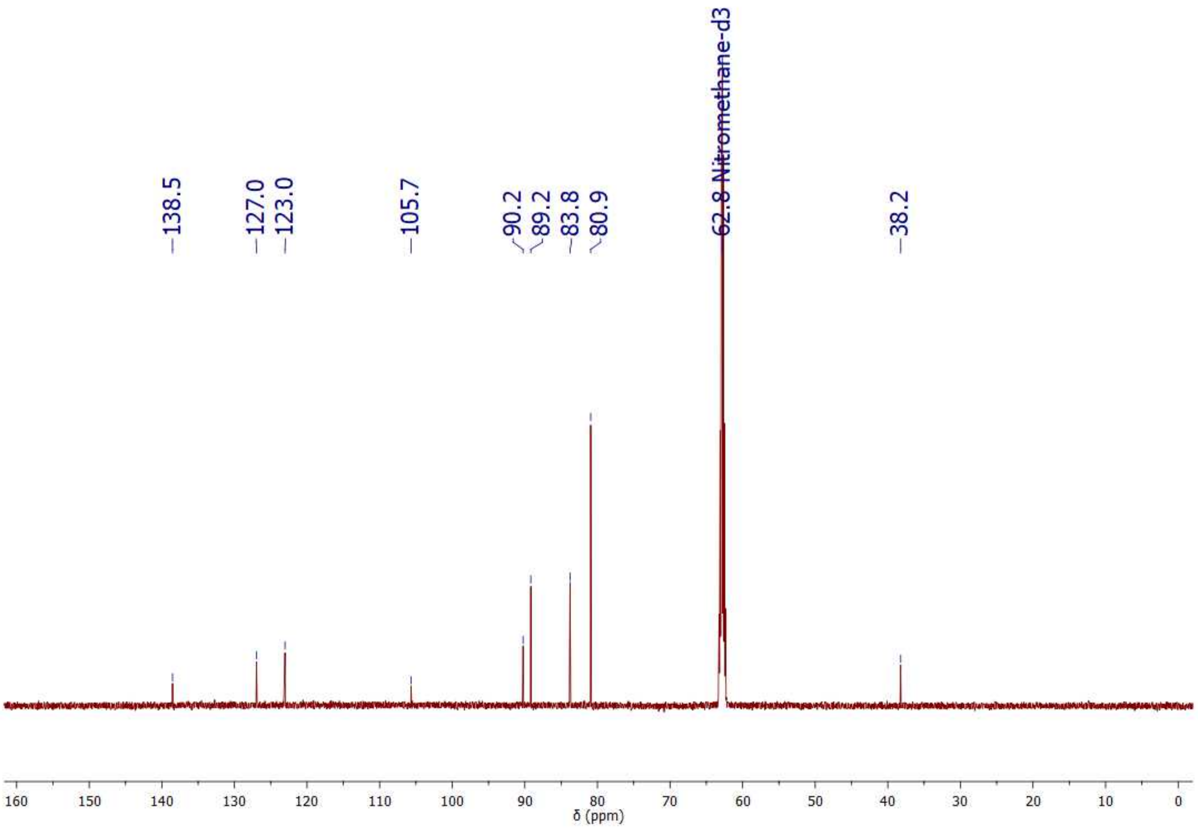

Figure S11: ^{31}P NMR spectrum of **3**.

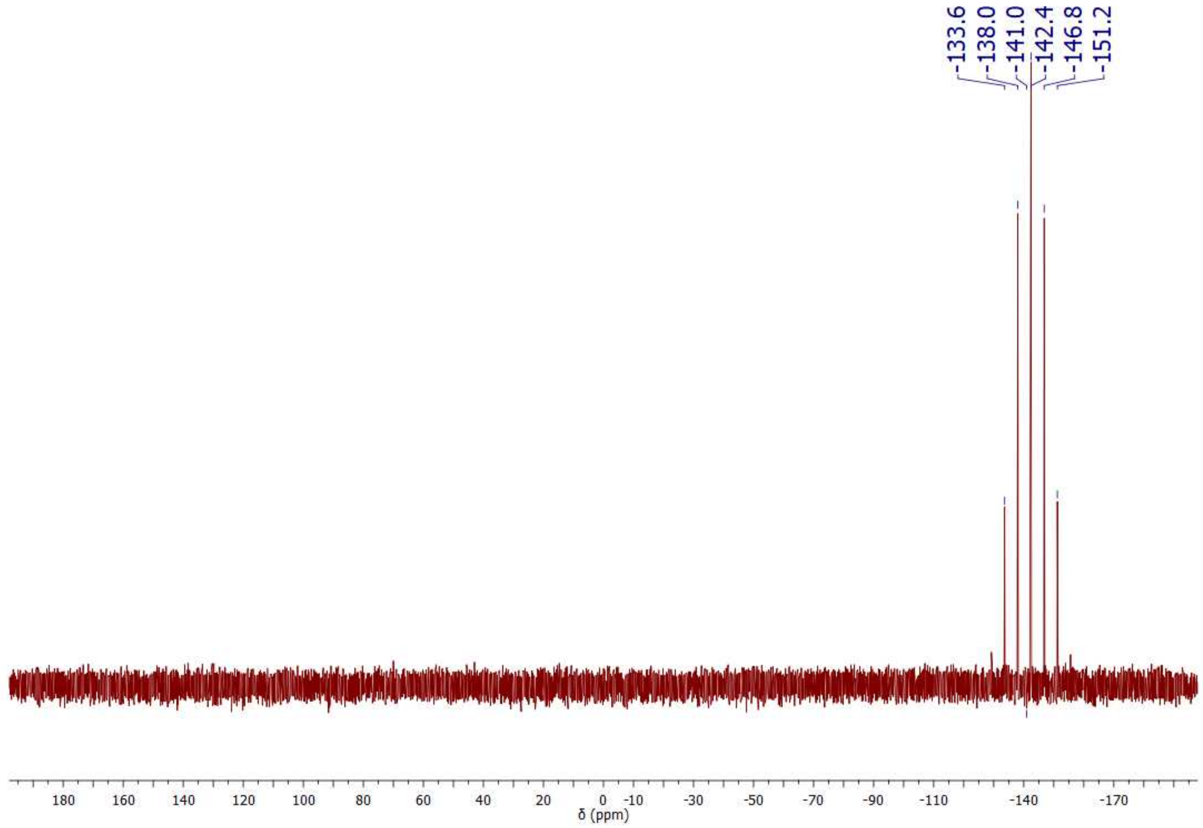

Figure S12: ESI-MS overview spectrum of **3**.

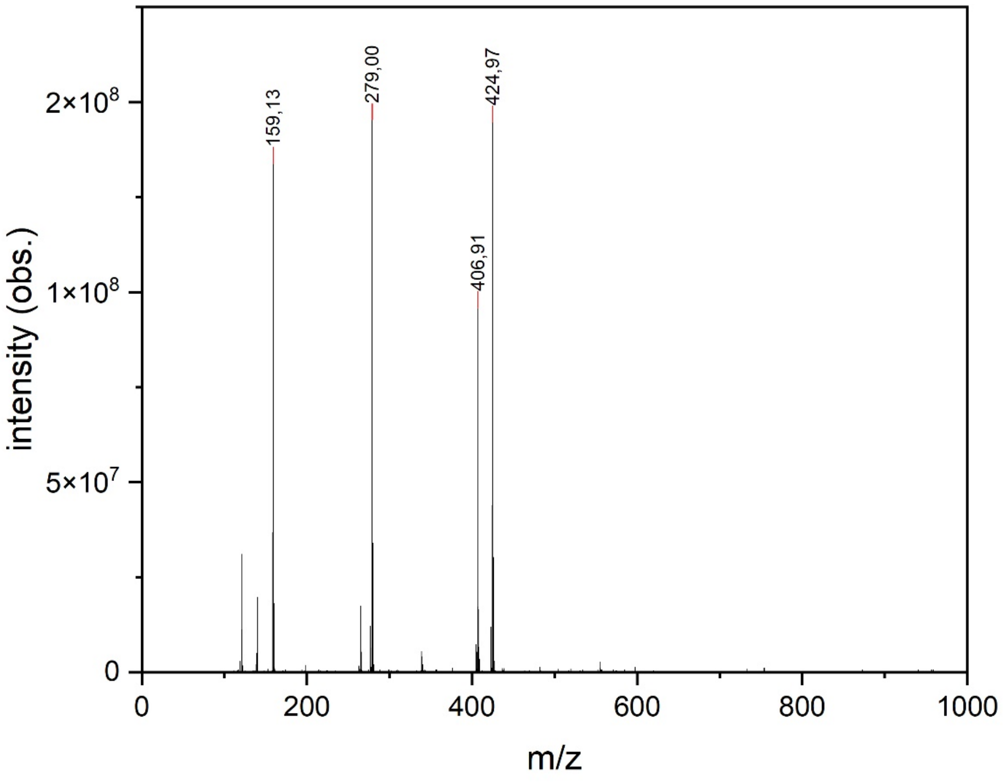

Figure S13: ESI-MS isolation of m/z = 425 in comparison with simulation of $[C_{15}H_{16}FeN_2PF_6]^+$.

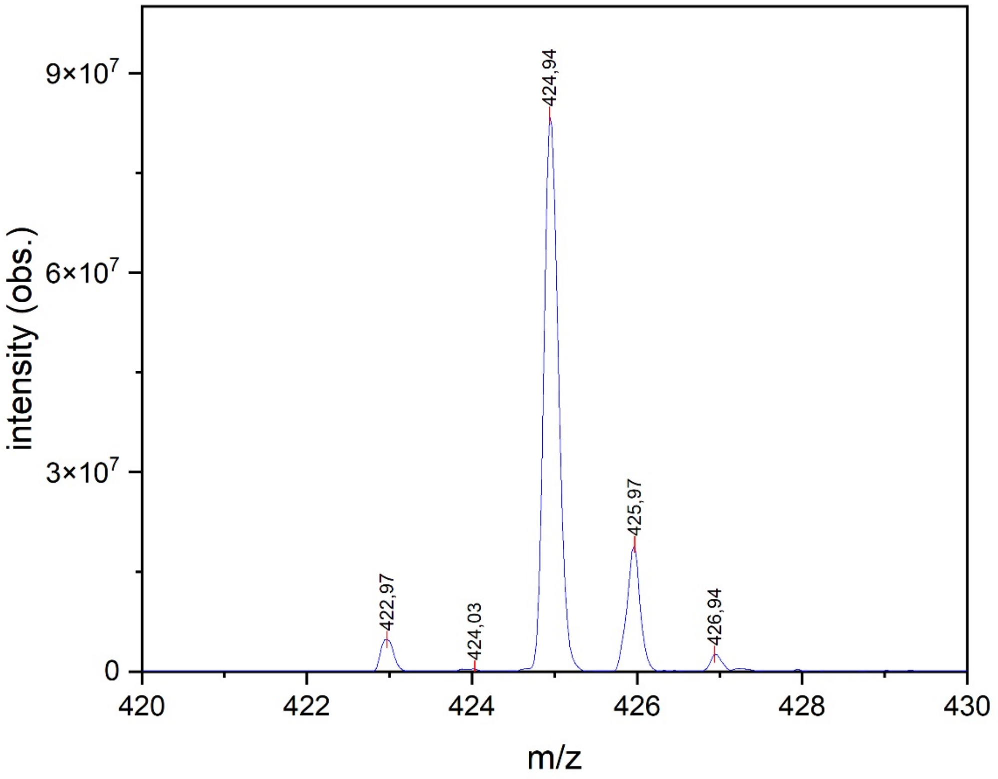

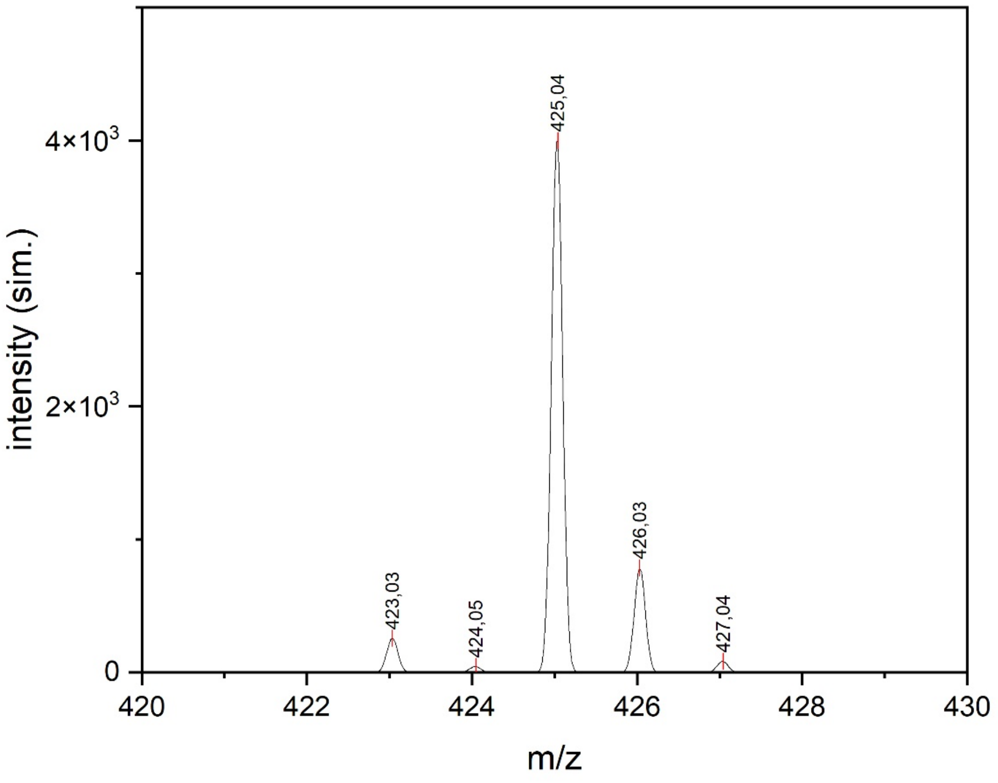

Figure S14: ESI-MS isolation of m/z = 407 in comparison with a simulation of $[C_{15}H_{16}FeN_2I]^+$.

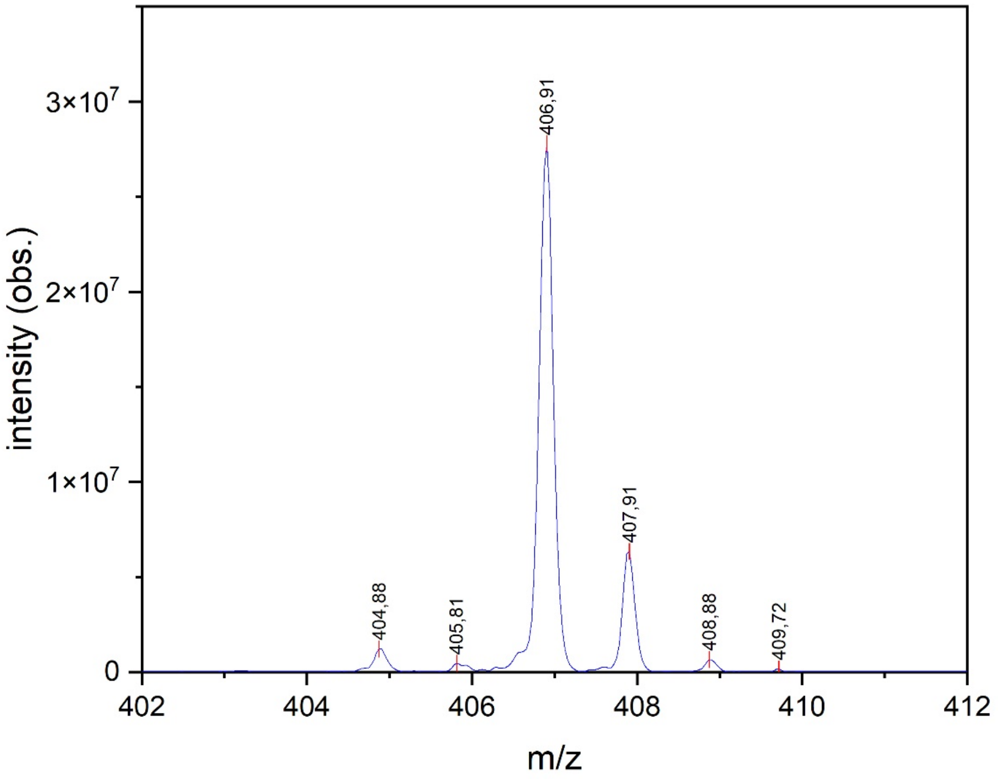

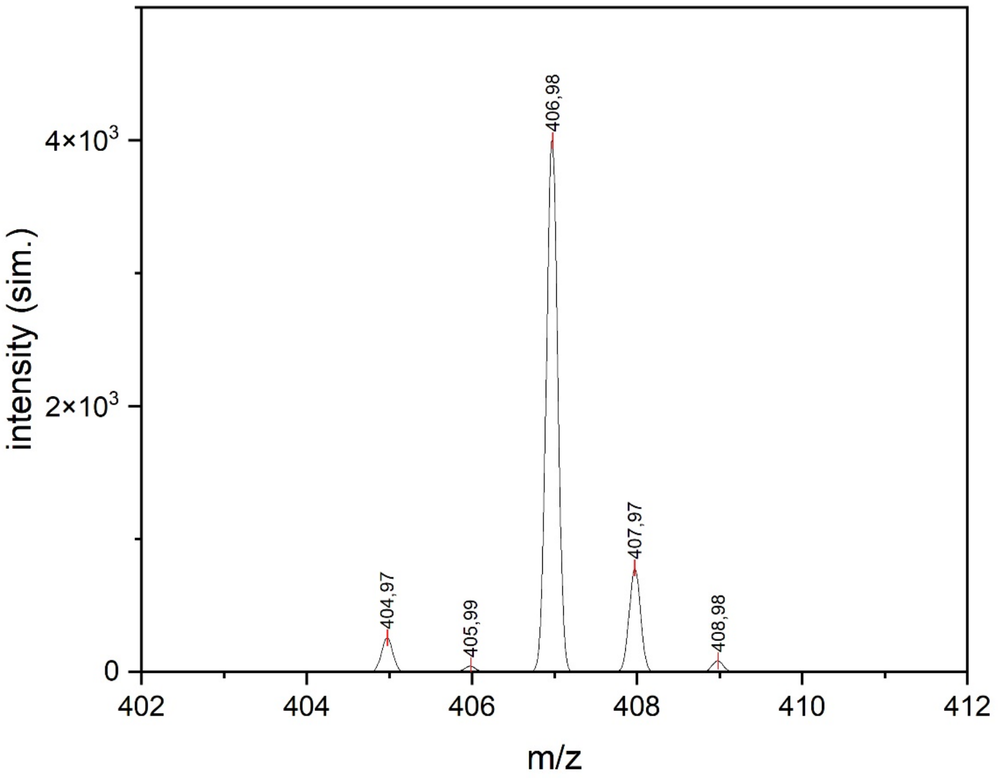

Figure S15: ESI-MS isolation of m/z = 279 in comparison with a simulation of $[C_{15}H_{15}FeN_2]^+$.

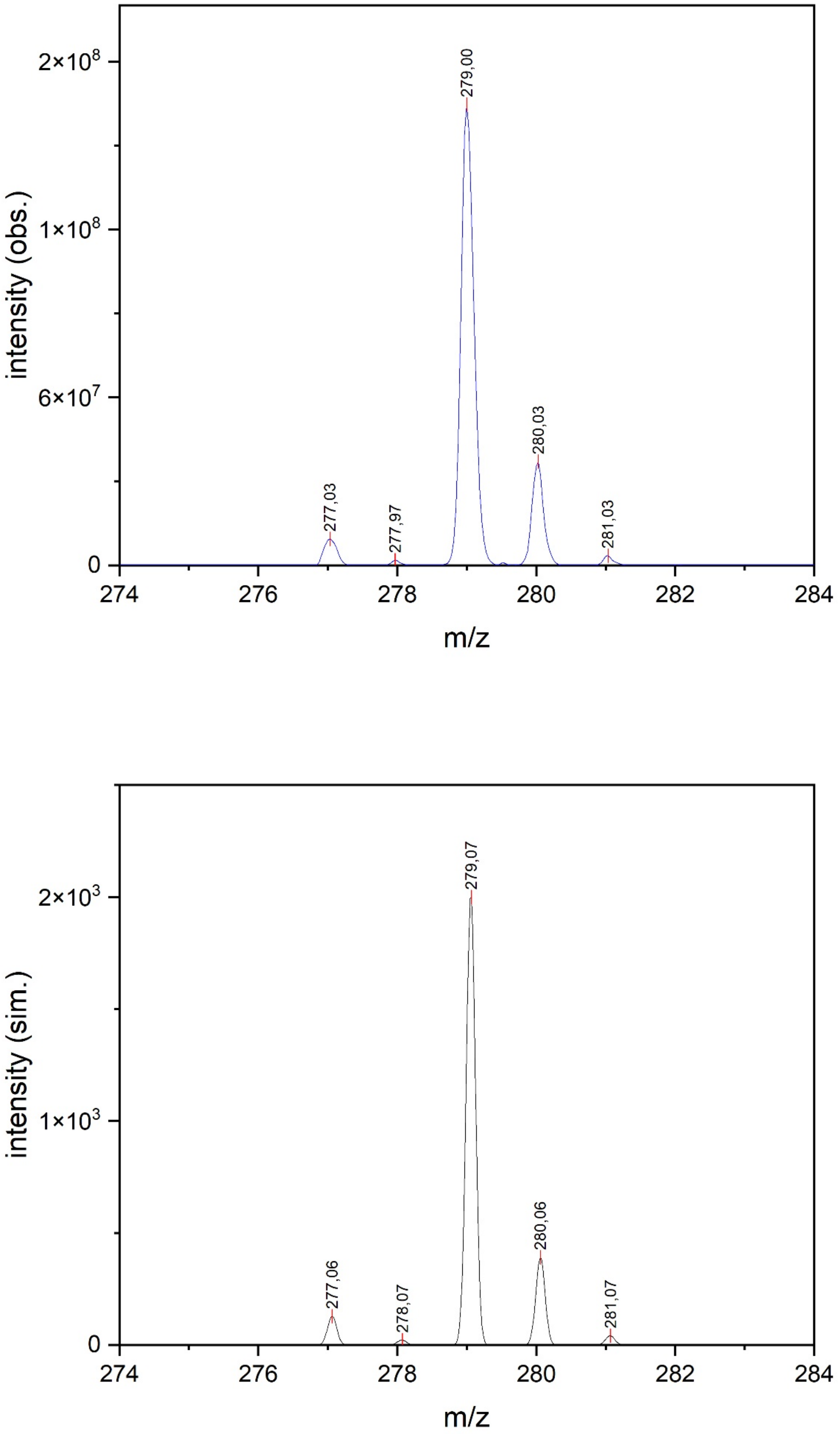

[Iodo((η^5-cyclopentadienyl)κ^{2}C^{2,7}(η^6-1-phenyl-3-methylimidazol-2-yliden)iron(II))-(η^5-1,2,3,4,5-pentamethylcyclopentadienyl)iridium(III) Hexafluorophosphate (4)

Figure S16: ^{1}H NMR spectrum of **4**.

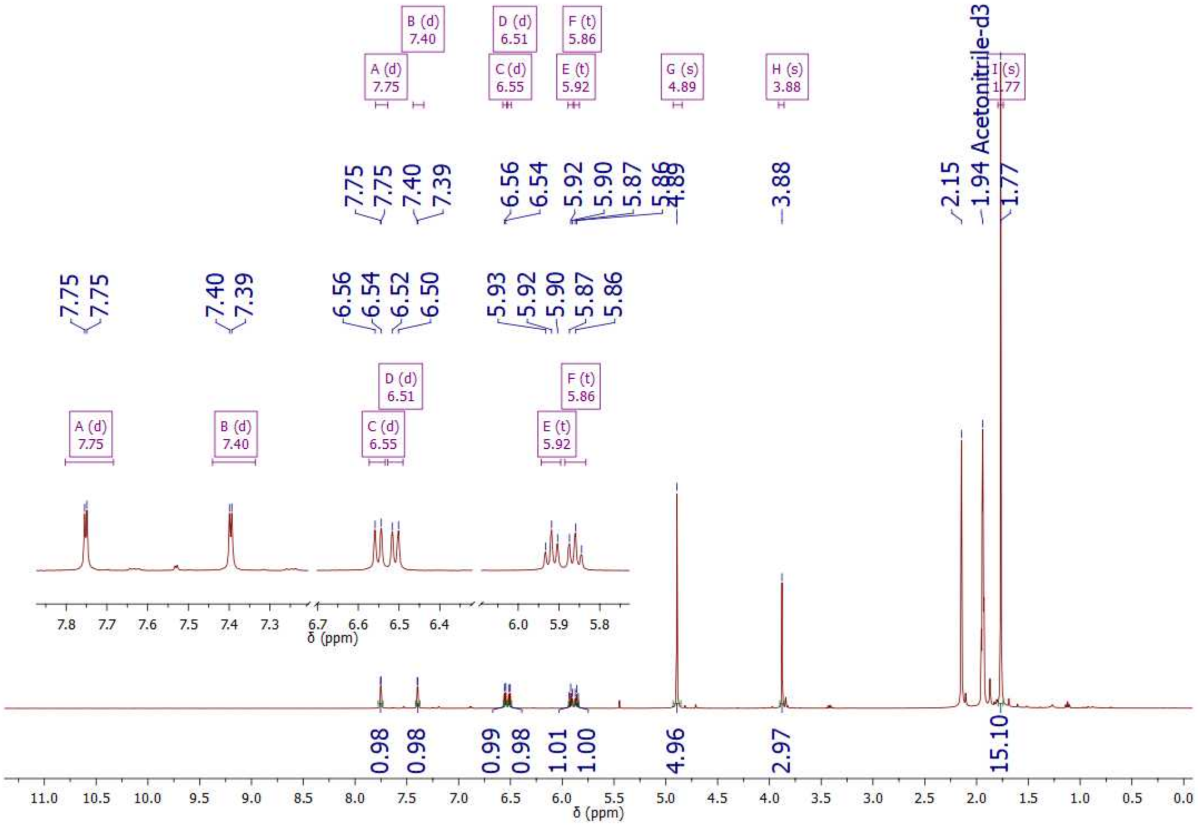

Figure S17: ^{13}C NMR spectrum of **4**.

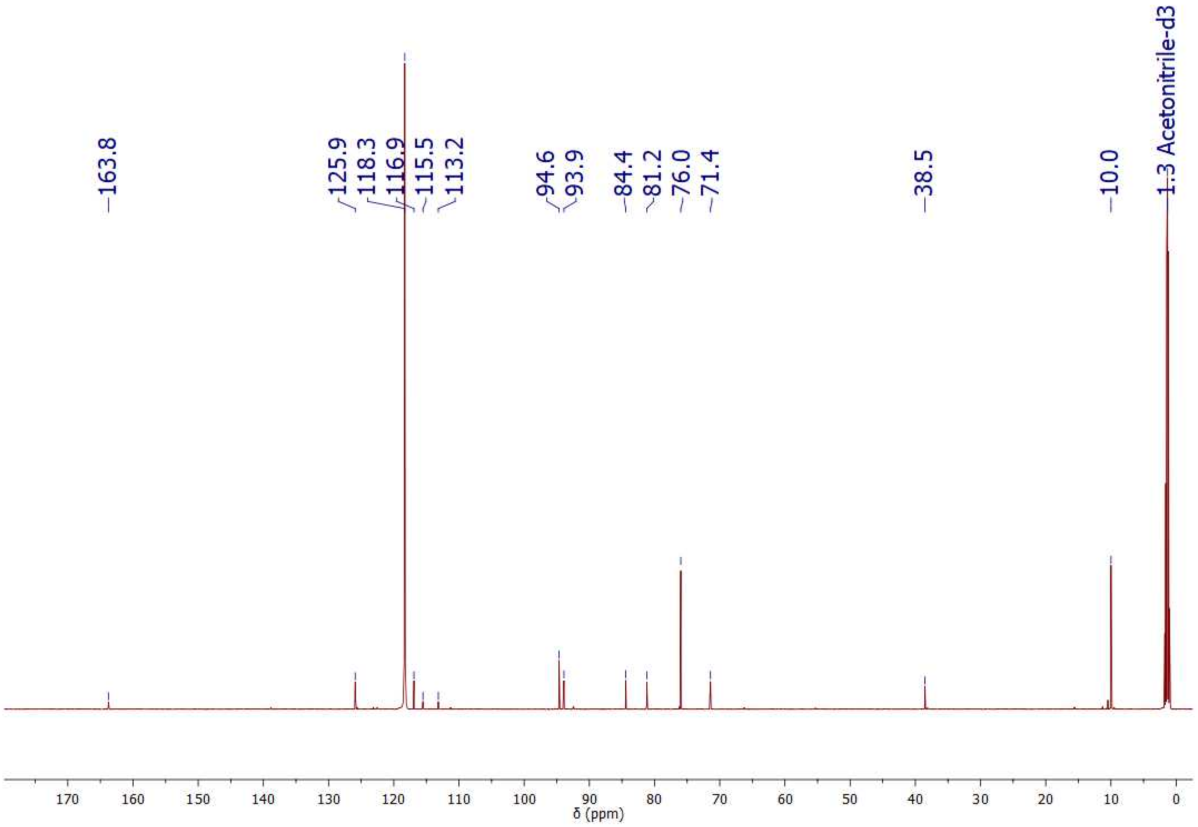

Figure S18: ^{31}P NMR spectrum of **4**.

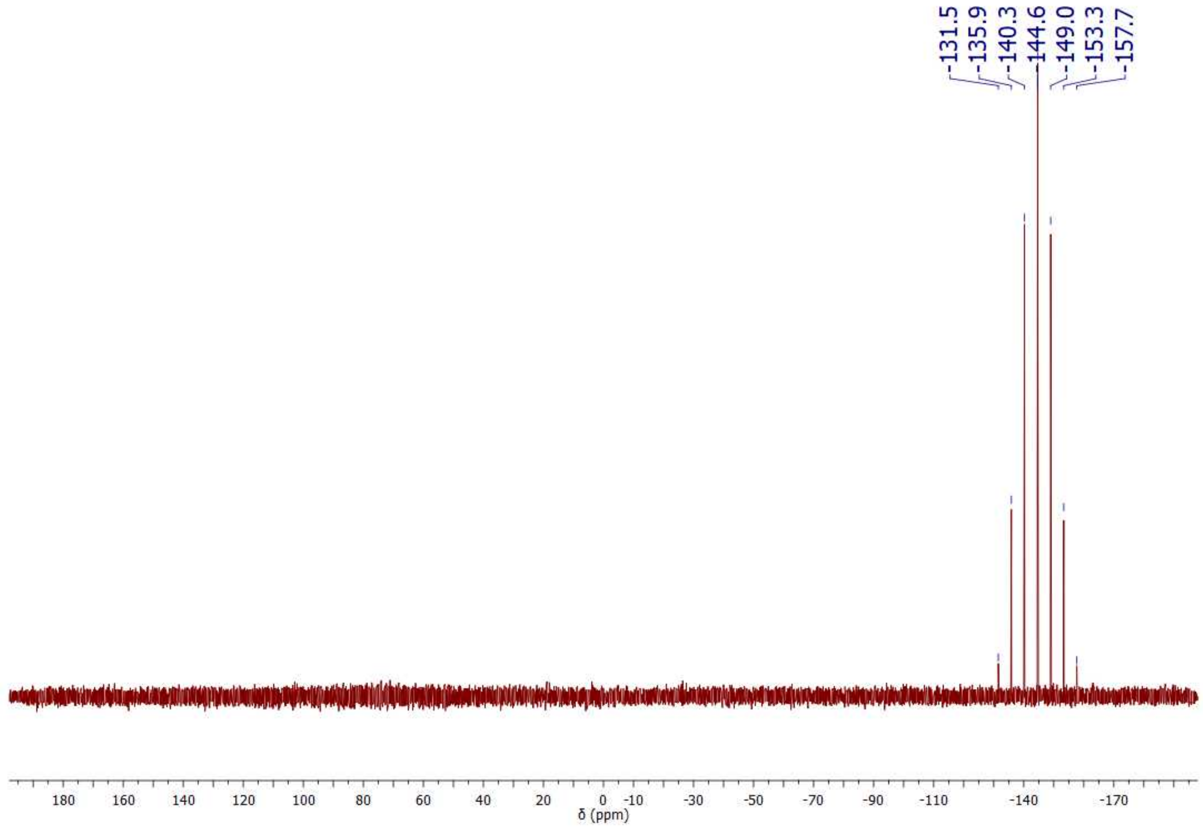

Figure S19: ESI-MS overview spectrum of **4**.

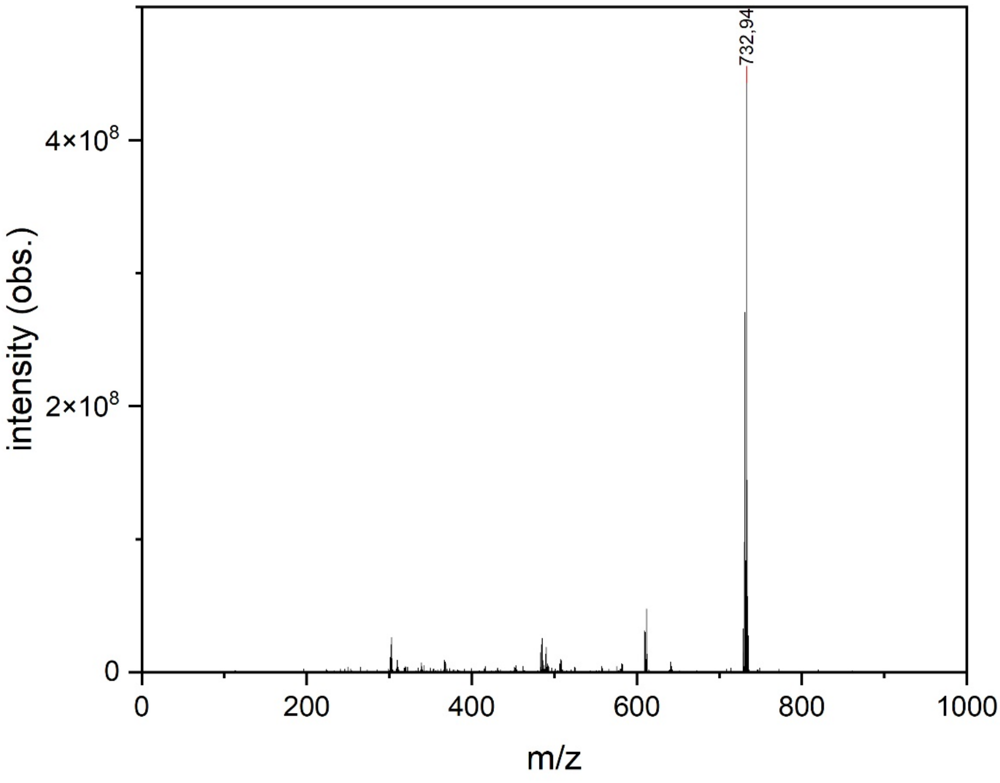

Figure S20: ESI-MS isolation of m/z = 733 in comparison with a simulation of $[C_{25}H_{29}FeIrN_2I]^+$.

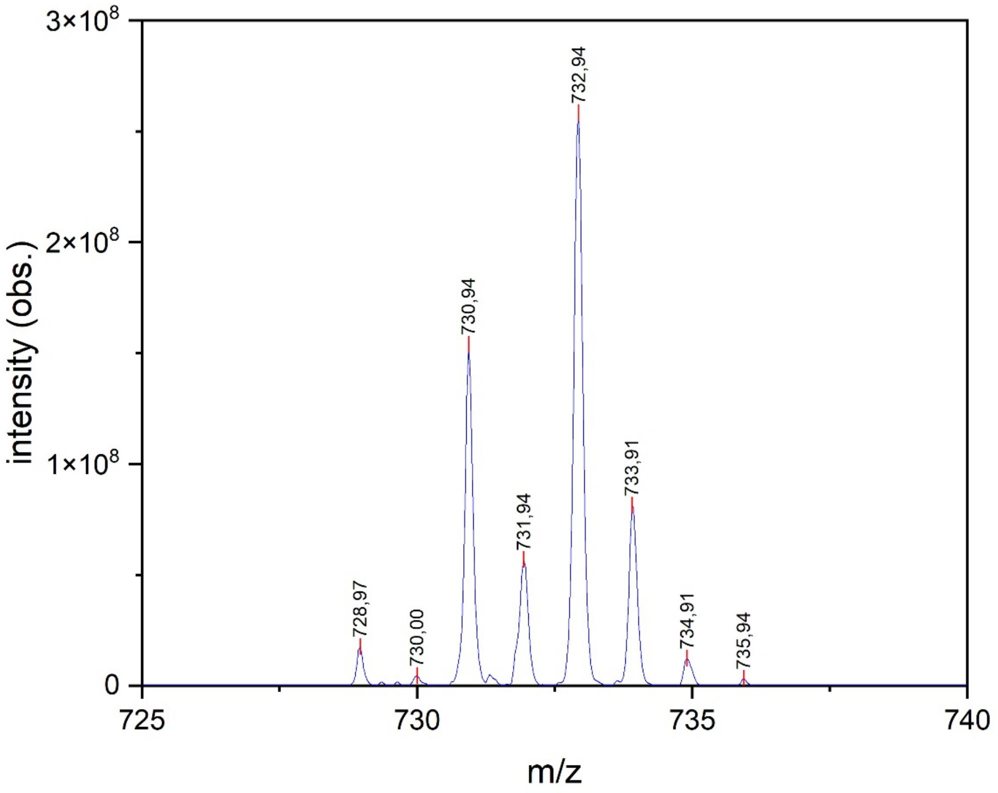

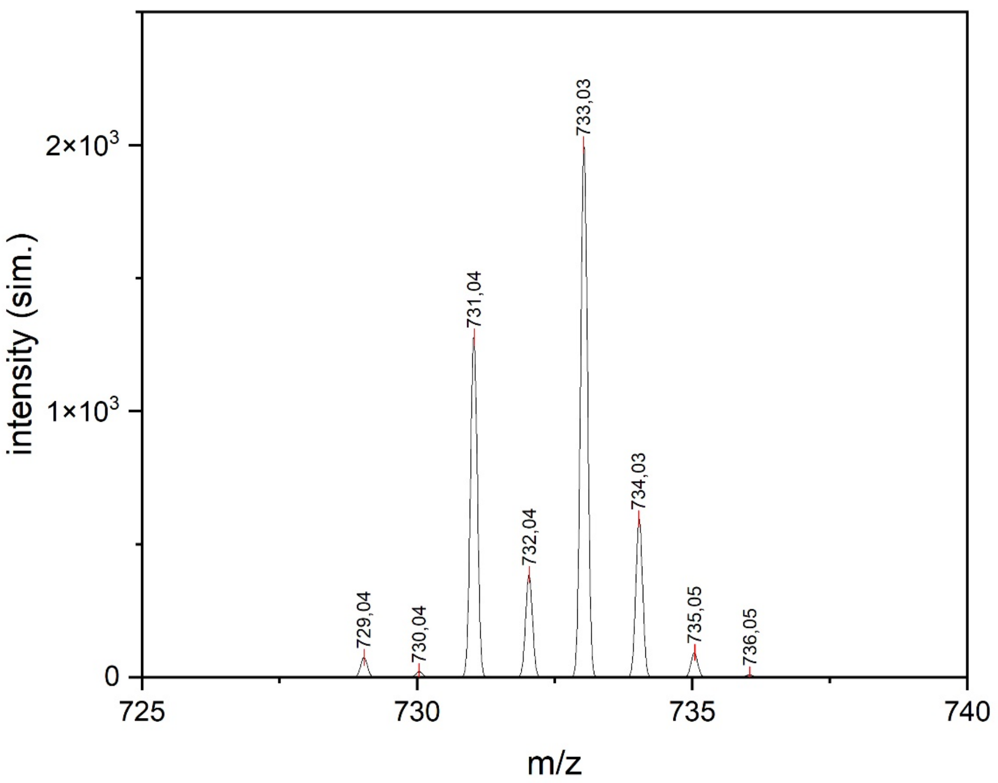

2. Catalytic Studies

2.1 Table 1, Entry #1

Figure S21: GC chromatogram of Acetophenone.

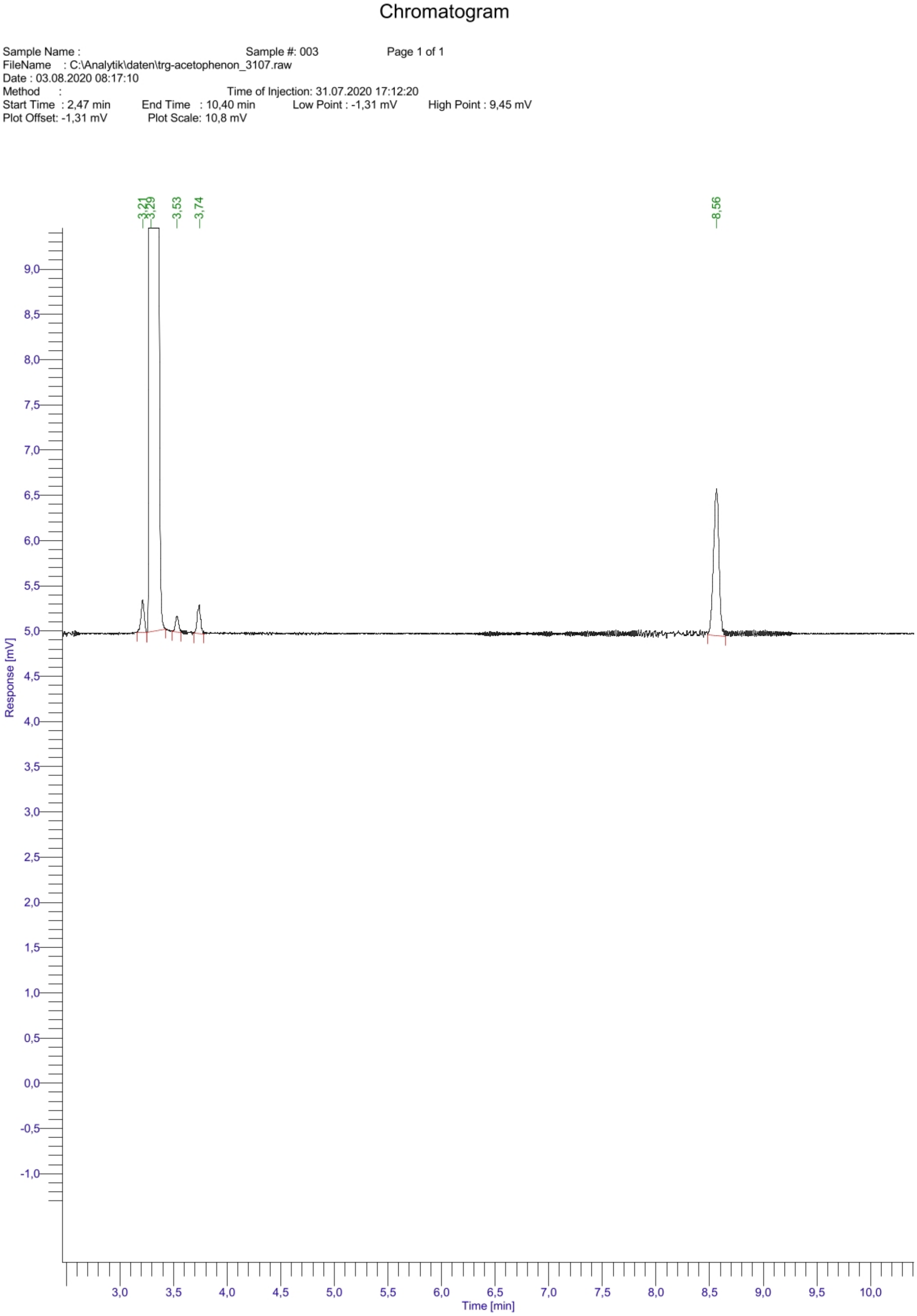

Software Version	: 6.3.2.0646		Date	: 03.08.2020 08:16:21
Operator	: Messaccount		Sample Name	:
Sample Number	: 003		Study	:
AutoSampler	: BUILT-IN		Rack/Vial	: 0/3
Instrument Name	: GC Clarus 580		Channel	: A
Instrument Serial #	: 580S12092501		A/D mV Range	: 1000
Delay Time	: 0,00 min		End Time	: 20,00 min
Sampling Rate	: 12,5000 pts/s			
Sample Volume	: 1,000000 ul			
Sample Amount	: 1,0000		Area Reject	: 0,000000
Data Acquisition Time	: 31.07.2020 17:12:20		Dilution Factor	: 1,00
			Cycle	: 3

Raw Data File : C:\Analytik\daten\trg-acetophenon_3107.raw
Result File : C:\Analytik\daten\trg-acetophenon_3107-20200803-081315.rst [Editing in Progress]
Inst Method : C:\Analytik\methoden\STANDARD_4K_aufheizrate from
C:\Analytik\daten\trg-acetophenon_3107.raw
Proc Method : C:\Analytik\methoden\STANDARD_4K_aufheizrate.mth from
C:\Analytik\daten\trg-acetophenon_3107-20200803-081315.rst [Editing in Progress]
Calib Method : C:\Analytik\methoden\STANDARD_4K_aufheizrate.mth from
C:\Analytik\daten\trg-acetophenon_3107-20200803-081315.rst [Editing in Progress]
Report Format File: C:\Analytik\methoden\STANDARD_4K_aufheizrate.rpt
Sequence File : C:\Analytik\sequenzen\trg231072020.seq

DEFAULT REPORT

Peak #	Time [min]	Area [µV·s]	Height [µV]	Area [%]	Norm. Area [%]	BL	Area/Height [s]
1	3,212	746,05	357,29	0,06	0,06	BB	2,0880
2	3,288	1335196,14	556812,86	99,43	99,43	BB	2,3979
3	3,532	364,19	178,34	0,03	0,03	BB	2,0421
4	3,739	712,19	316,50	0,05	0,05	BB	2,2502
5	8,565	5837,98	1618,34	0,43	0,43	BB	3,6074
		1342856,55	559283,34	100,00	100,00		

Missing Component Report

Component	Expected Retention (Calibration File)

All components were found

Figure S22: GC chromatogram of 1-Phenylethanol.

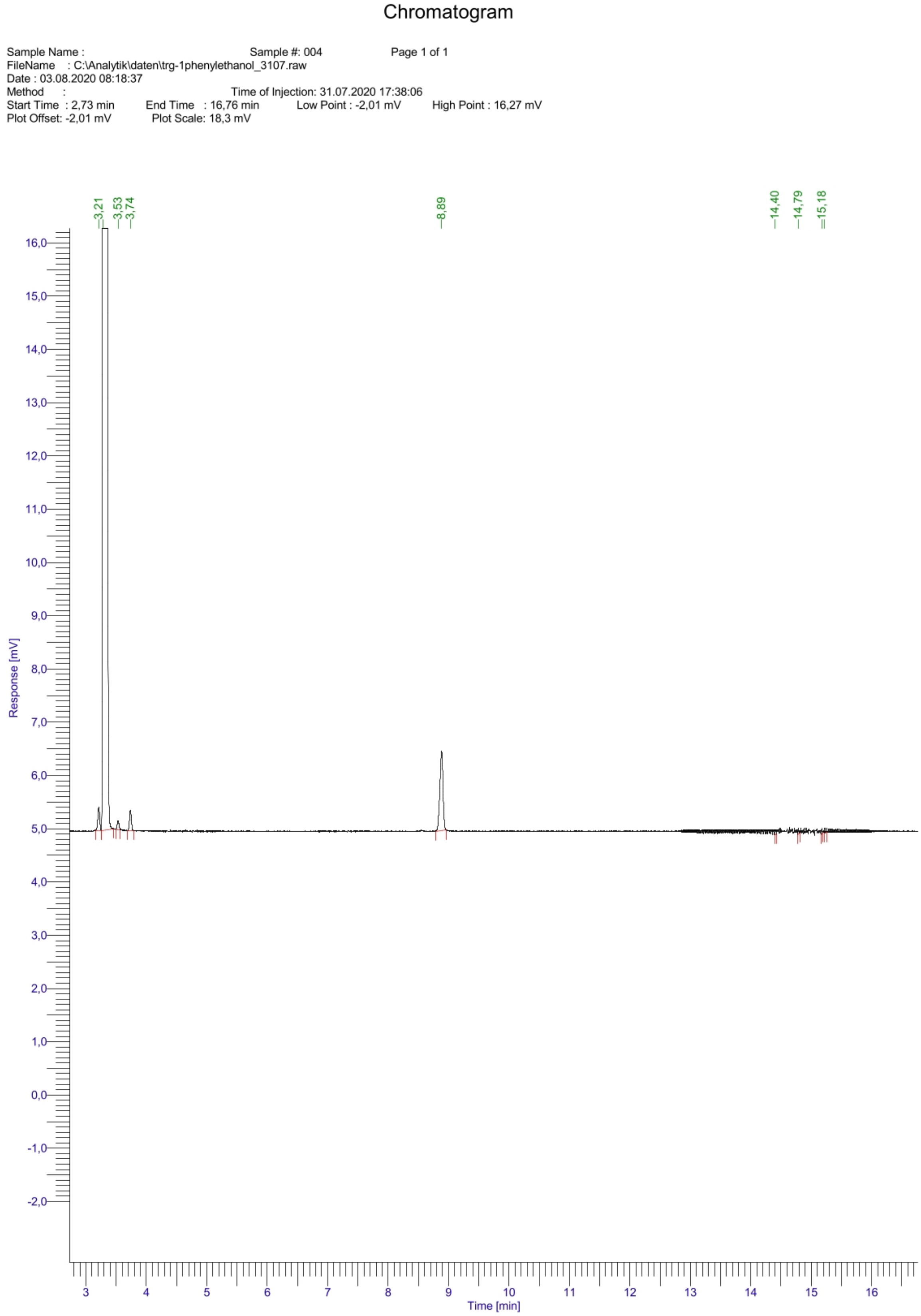

Software Version	: 6.3.2.0646		Date	:	03.08.2020 08:17:28
Operator	: Messaccount		Sample Name	:	
Sample Number	: 004		Study	:	
AutoSampler	: BUILT-IN		Rack/Vial	:	0/4
Instrument Name	: GC Clarus 580		Channel	:	A
Instrument Serial #	: 580S12092501		A/D mV Range	:	1000
Delay Time	: 0,00 min		End Time	:	20,00 min
Sampling Rate	: 12,5000 pts/s				
Sample Volume	: 1,000000 ul				
Sample Amount	: 1,0000		Area Reject	:	0,000000
Data Acquisition Time	: 31.07.2020 17:38:06		Dilution Factor	:	1,00
			Cycle	:	4

Raw Data File : C:\Analytik\daten\trg-1phenylethanol_3107.raw
Result File : C:\Analytik\daten\trg-1phenylethanol_3107-20200803-081317.rst [Editing in Progress]
Inst Method : C:\Analytik\methoden\STANDARD_4K_aufheizrate from
C:\Analytik\daten\trg-1phenylethanol_3107.raw
Proc Method : C:\Analytik\methoden\STANDARD_4K_aufheizrate.mth from
C:\Analytik\daten\trg-1phenylethanol_3107-20200803-081317.rst [Editing in Progress]
Calib Method : C:\Analytik\methoden\STANDARD_4K_aufheizrate.mth from
C:\Analytik\daten\trg-1phenylethanol_3107-20200803-081317.rst [Editing in Progress]
Report Format File: C:\Analytik\methoden\STANDARD_4K_aufheizrate.rpt
Sequence File : C:\Analytik\sequenzen\trg231072020.seq

DEFAULT REPORT

Peak #	Time [min]	Area [µV·s]	Height [µV]	Area [%]	Norm. Area [%]	BL	Area/Height [s]
1	3,213	918,25	434,03	0,07	0,07	BB	2,1156
2	3,288	1340611,14	557373,42	99,41	99,41	BB	2,4052
3	3,534	346,54	169,38	0,03	0,03	BB	2,0459
4	3,736	831,29	385,08	0,06	0,06	BB	2,1588
5	8,886	5439,70	1488,61	0,40	0,40	BB	3,6542
6	14,400	127,18	89,02	0,01	0,01	BB	1,4286
7	14,790	115,41	110,86	0,01	0,01	BB	1,0411
8	15,179	92,06	93,72	0,01	0,01	BB	0,9823
9	15,225	102,82	96,91	0,01	0,01	BB	1,0610
		1348584,40	560241,03	100,00	100,00		

Missing Component Report

Component	Expected Retention (Calibration File)

All components were found

Figure S23: GC chromatogram of catalysis #1 after 10 min.

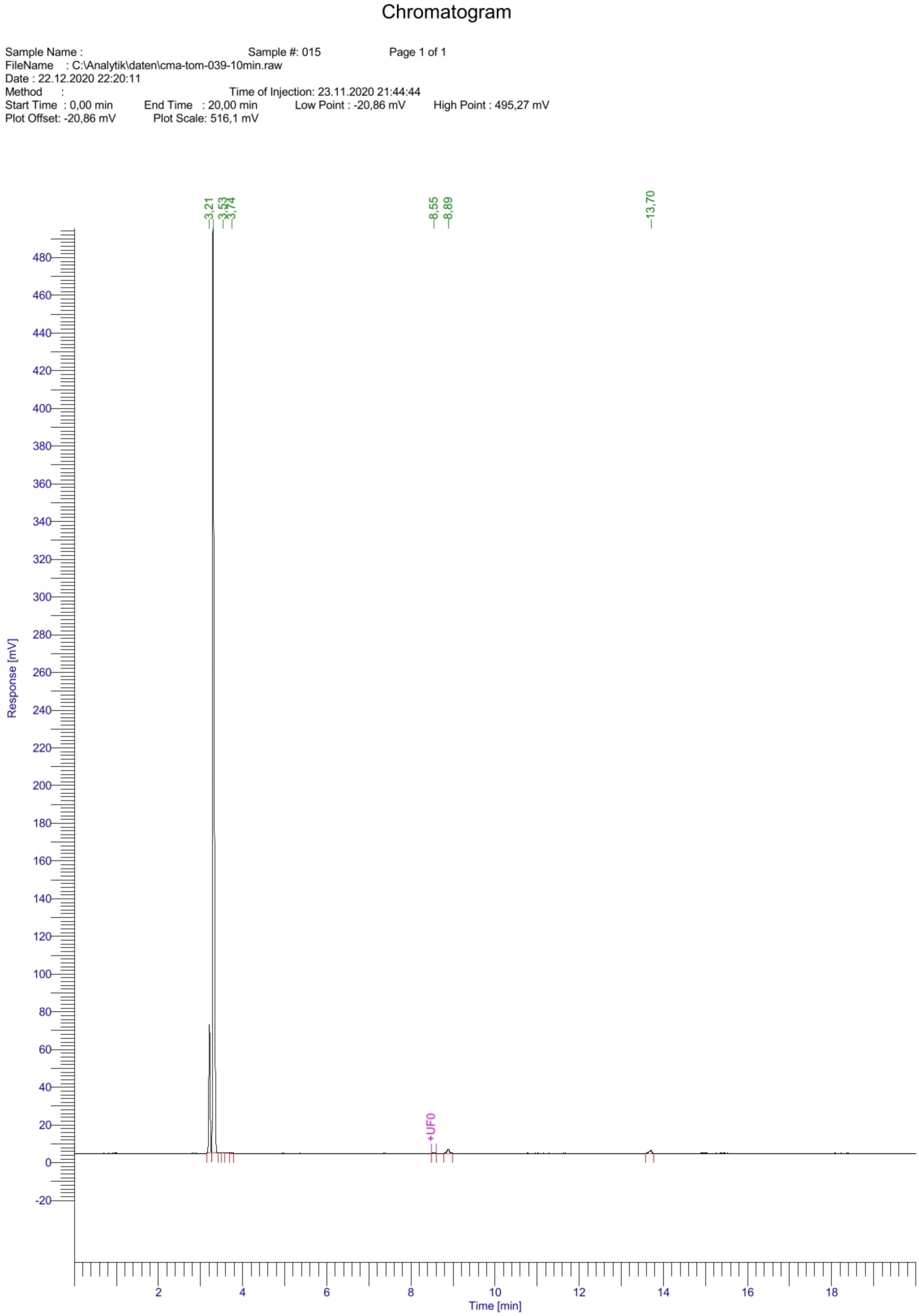

Software Version	: 6.3.2.0646		Date	: 15.12.2020 12:30:31
Operator	: Messaccount		Sample Name	:
Sample Number	: 015		Study	:
AutoSampler	: BUILT-IN		Rack/Vial	: 0/15
Instrument Name	: GC Clarus 580		Channel	: A
Instrument Serial #	: None		A/D mV Range	: 1000
Delay Time	: 0,00 min		End Time	: 20,00 min
Sampling Rate	: 12,5000 pts/s			
Sample Volume	: 1,000000 ul			
Sample Amount	: 1,0000		Area Reject	: 0,000000
Data Acquisition Time	: 23.11.2020 21:44:44		Dilution Factor	: 1,00
			Cycle	: 15

Raw Data File : C:\Analytik\daten\cma-tom-039-10min.raw
Result File : C:\Analytik\daten\cma-tom-039-10min-20201124-151552.rst [Editing in Progress]
Inst Method : C:\Analytik\methoden\STANDARD_4K_aufheizrate from
C:\Analytik\daten\cma-tom-039-10min.raw
Proc Method : C:\Analytik\methoden\STANDARD_4K_aufheizrate.mth from
C:\Analytik\daten\cma-tom-039-10min-20201124-151552.rst [Editing in Progress]
Calib Method : C:\Analytik\methoden\STANDARD_4K_aufheizrate.mth from
C:\Analytik\daten\cma-tom-039-10min-20201124-151552.rst [Editing in Progress]
Report Format File: C:\Analytik\methoden\STANDARD_4K_aufheizrate.rpt
Sequence File : C:\Analytik\sequenzen\cmann23112020.seq

DEFAULT REPORT

Peak #	Time [min]	Area [µV·s]	Height [µV]	Area [%]	Norm. Area [%]	BL	Area/Height [s]
1	3,209	143710,27	67987,37	10,61	10,61	BB	2,1138
2	3,292	1194303,64	489995,67	88,18	88,18	BB	2,4374
3	3,532	292,23	140,17	0,02	0,02	BB	2,0849
4	3,737	929,74	408,50	0,07	0,07	BB	2,2760
5	8,548	312,66	97,01	0,02	0,02	MM	3,2231
6	8,891	8221,02	2107,72	0,61	0,61	BB	3,9004
7	13,705	6608,65	1603,75	0,49	0,49	BB	4,1207
		1354378,20	562340,19	100,00	100,00		

Missing Component Report

Component	Expected Retention (Calibration File)

All components were found

2.2 Table 1, Entry #2

Figure S24: GC chromatogram of 2-Hexanone.

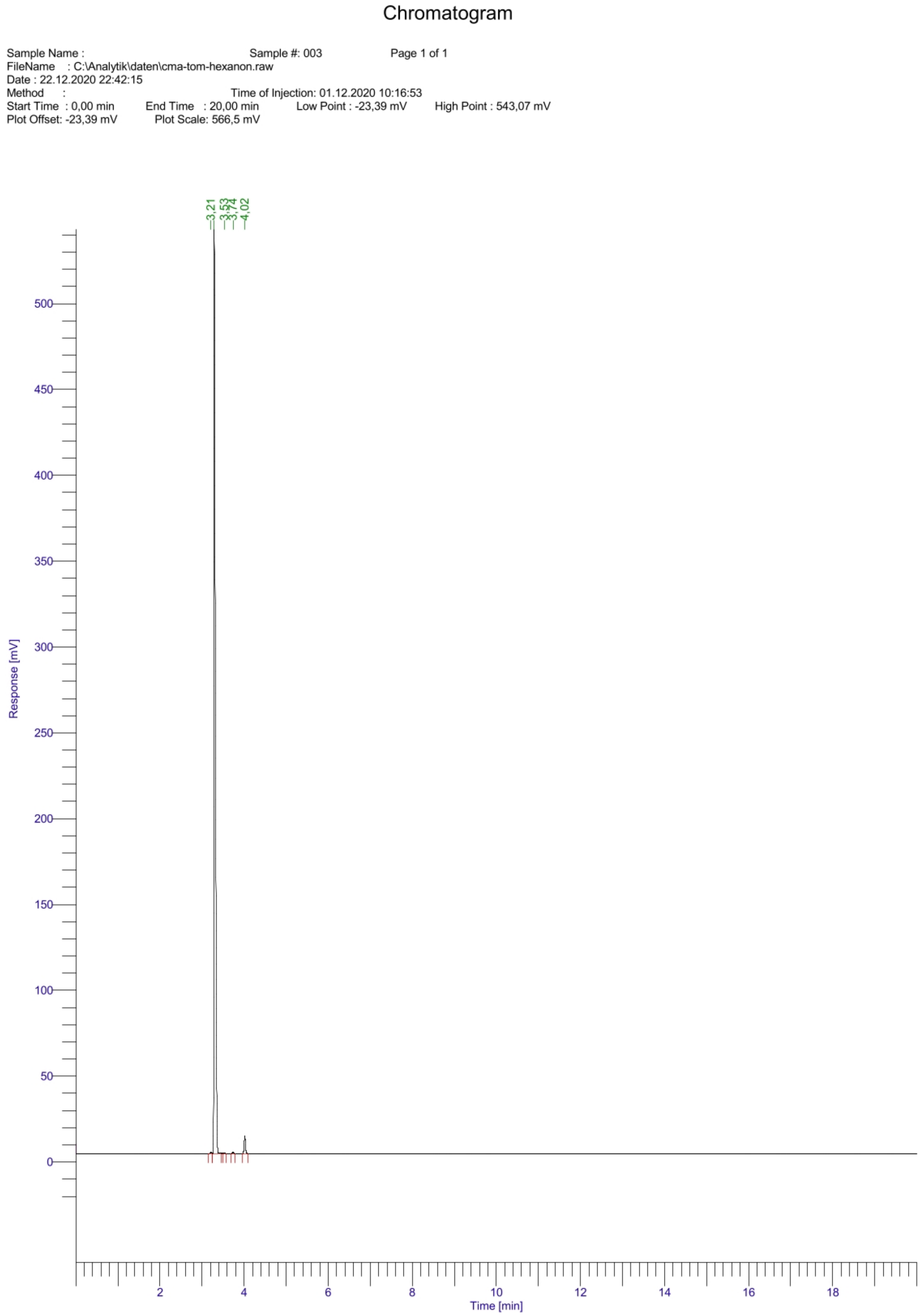

Software Version	: 6.3.2.0646		Date	: 01.12.2020 12:18:49
Operator	: Messaccount		Sample Name	:
Sample Number	: 003		Study	:
AutoSampler	: BUILT-IN		Rack/Vial	: 0/3
Instrument Name	: GC Clarus 580		Channel	: A
Instrument Serial #	: None		A/D mV Range	: 1000
Delay Time	: 0,00 min		End Time	: 20,00 min
Sampling Rate	: 12,5000 pts/s			
Sample Volume	: 1,000000 ul			
Sample Amount	: 1,0000		Area Reject	: 0,000000
Data Acquisition Time	: 01.12.2020 10:16:53		Dilution Factor	: 1,00
			Cycle	: 3

Raw Data File : C:\Analytik\daten\cma-tom-hexanon.raw
Result File : C:\Analytik\daten\cma-tom-hexanon-20201201-121404.rst [Editing in Progress]
Inst Method : C:\Analytik\methoden\STANDARD_4K_aufheizrate from C:\Analytik\daten\cma-tom-hexanon.raw
Proc Method : C:\Analytik\methoden\STANDARD_4K_aufheizrate.mth from C:\Analytik\daten\cma-tom-hexanon-20201201-121404.rst [Editing in Progress]
Calib Method : C:\Analytik\methoden\STANDARD_4K_aufheizrate.mth from C:\Analytik\daten\cma-tom-hexanon-20201201-121404.rst [Editing in Progress]
Report Format File: C:\Analytik\methoden\STANDARD_4K_aufheizrate.rpt
Sequence File : C:\Analytik\sequenzen\cma01122020.seq

DEFAULT REPORT

Peak #	Time [min]	Area [µV·s]	Height [µV]	Area [%]	Norm. Area [%]	BL	Area/Height [s]
1	3,212	1192,56	584,46	0,09	0,09	BV	2,0404
2	3,289	1282535,82	538235,00	98,07	98,07	VB	2,3829
3	3,533	365,87	167,53	0,03	0,03	BB	2,1840
4	3,737	1244,85	559,06	0,10	0,10	BB	2,2267
5	4,016	22405,04	10330,30	1,71	1,71	BB	2,1689
		1307744,13	549876,34	100,00	100,00		

Missing Component Report

Component	Expected Retention (Calibration File)

All components were found

Figure S25: GC chromatogram of 2-Hexanol.

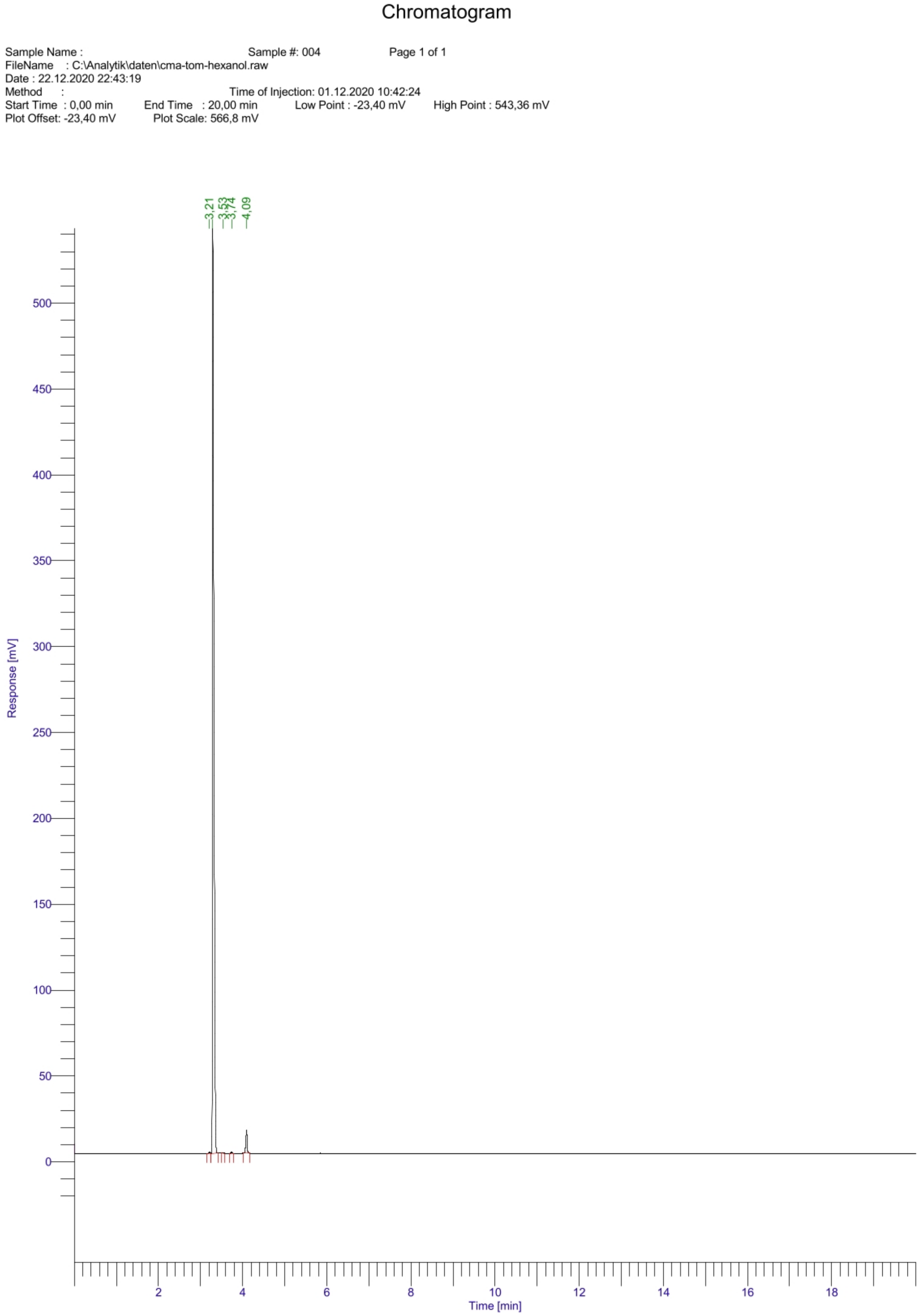

Software Version	: 6.3.2.0646		Date	: 01.12.2020 12:19:30
Operator	: Messaccount		Sample Name	:
Sample Number	: 004		Study	:
AutoSampler	: BUILT-IN		Rack/Vial	: 0/4
Instrument Name	: GC Clarus 580		Channel	: A
Instrument Serial #	: None		A/D mV Range	: 1000
Delay Time	: 0,00 min		End Time	: 20,00 min
Sampling Rate	: 12,5000 pts/s			
Sample Volume	: 1,000000 ul		Area Reject	: 0,000000
Sample Amount	: 1,0000		Dilution Factor	: 1,00
Data Acquisition Time	: 01.12.2020 10:42:24		Cycle	: 4

Raw Data File : C:\Analytik\daten\cma-tom-hexanol.raw
Result File : C:\Analytik\daten\cma-tom-hexanol-20201201-121406.rst [Editing in Progress]
Inst Method : C:\Analytik\methoden\STANDARD_4K_aufheizrate from
C:\Analytik\daten\cma-tom-hexanol.raw
Proc Method : C:\Analytik\methoden\STANDARD_4K_aufheizrate.mth from
C:\Analytik\daten\cma-tom-hexanol-20201201-121406.rst [Editing in Progress]
Calib Method : C:\Analytik\methoden\STANDARD_4K_aufheizrate.mth from
C:\Analytik\daten\cma-tom-hexanol-20201201-121406.rst [Editing in Progress]
Report Format File: C:\Analytik\methoden\STANDARD_4K_aufheizrate.rpt
Sequence File : C:\Analytik\sequenzen\cma01122020.seq

DEFAULT REPORT

Peak #	Time [min]	Area [µV·s]	Height [µV]	Area [%]	Norm. Area [%]	BL	Area/Height [s]
1	3,212	1295,71	633,64	0,10	0,10	BB	2,0449
2	3,289	1284853,48	538458,57	97,50	97,50	BB	2,3862
3	3,532	303,45	153,15	0,02	0,02	BB	1,9814
4	3,736	1385,57	635,90	0,11	0,11	BB	2,1789
5	4,093	29937,53	13430,39	2,27	2,27	BB	2,2291
		1317775,73	553311,64	100,00	100,00		

Missing Component Report

Component	Expected Retention (Calibration File)

All components were found

Figure S26: GC chromatogram of catalysis #2 after 15 min.

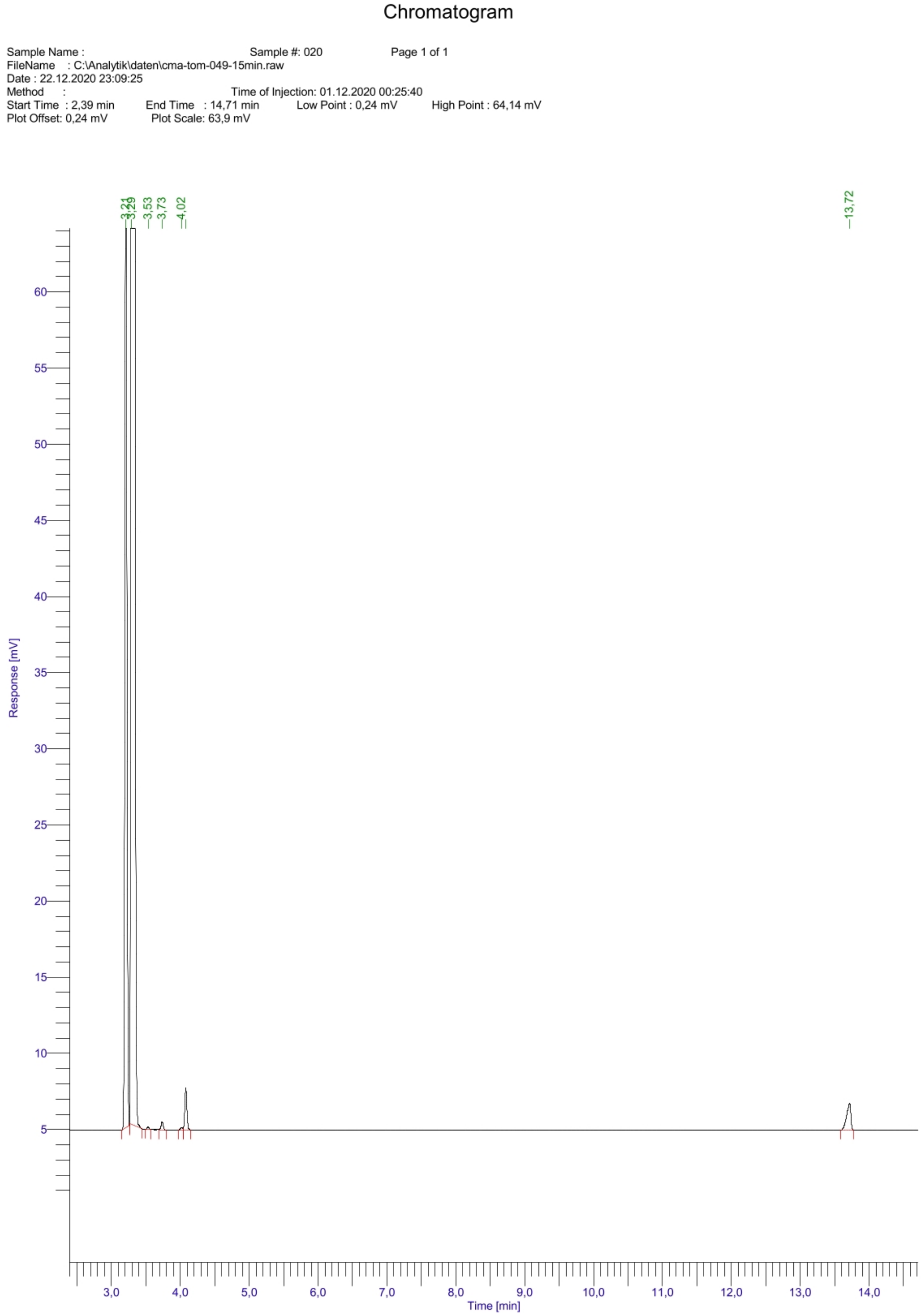

Chromatogram

Sample Name : Sample #: 020 Page 1 of 1
FileName : C:\Analytik\daten\cma-tom-049-15min.raw
Date : 22.12.2020 23:12:52
Method : Time of Injection: 01.12.2020 00:25:40
Start Time : 3,55 min End Time : 4,42 min Low Point : 2,89 mV High Point : 25,45 mV
Plot Offset: 2,89 mV Plot Scale: 22,6 mV

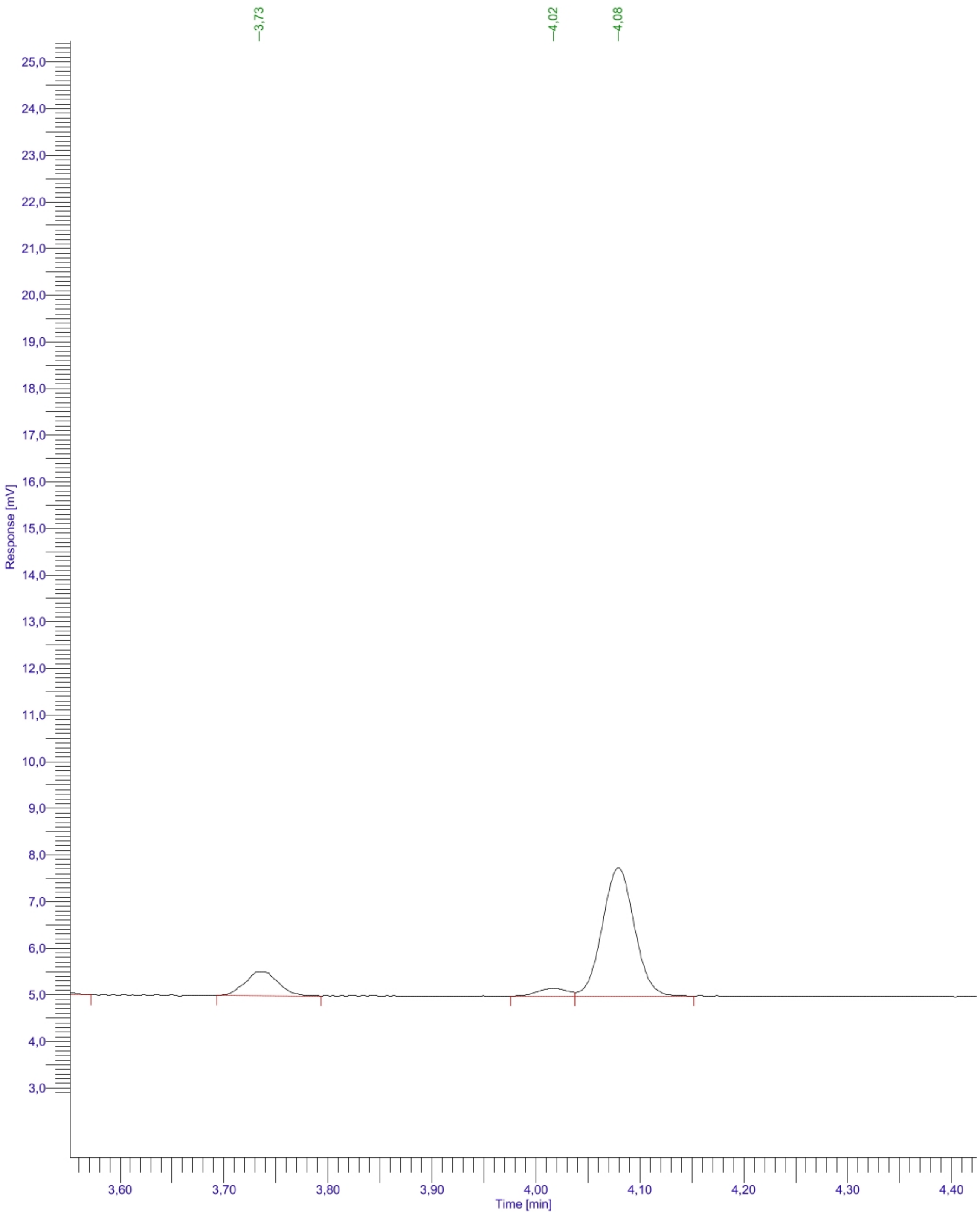

Software Version	: 6.3.2.0646		Date	: 01.12.2020 12:31:10
Operator	: Messaccount		Sample Name	:
Sample Number	: 020		Study	:
AutoSampler	: BUILT-IN		Rack/Vial	: 0/20
Instrument Name	: GC Clarus 580		Channel	: A
Instrument Serial #	: None		A/D mV Range	: 1000
Delay Time	: 0,00 min		End Time	: 20,00 min
Sampling Rate	: 12,5000 pts/s			
Sample Volume	: 1,000000 ul			
Sample Amount	: 1,0000		Area Reject	: 0,000000
Data Acquisition Time	: 01.12.2020 00:25:40		Dilution Factor	: 1,00
			Cycle	: 20

Raw Data File : C:\Analytik\daten\cma-tom-049-15min.raw
Result File : C:\Analytik\daten\cma-tom-049-15min-20201201-083423.rst [Editing in Progress]
Inst Method : C:\Analytik\methoden\STANDARD_4K_aufheizrate from
C:\Analytik\daten\cma-tom-049-15min.raw
Proc Method : C:\Analytik\methoden\STANDARD_4K_aufheizrate.mth from
C:\Analytik\daten\cma-tom-049-15min-20201201-083423.rst [Editing in Progress]
Calib Method : C:\Analytik\methoden\STANDARD_4K_aufheizrate.mth from
C:\Analytik\daten\cma-tom-049-15min-20201201-083423.rst [Editing in Progress]
Report Format File: C:\Analytik\methoden\STANDARD_4K_aufheizrate.rpt
Sequence File : C:\Analytik\sequenzen\nncma30112020.seq

DEFAULT REPORT

Peak #	Time [min]	Area [µV·s]	Height [µV]	Area [%]	Norm. Area [%]	BL	Area/Height [s]
1	3,209	137837,15	67376,85	10,57	10,57	BB	2,0458
2	3,292	1151694,49	494957,54	88,28	88,28	BB	2,3269
3	3,531	286,75	144,92	0,02	0,02	BB	1,9788
4	3,734	1149,25	514,78	0,09	0,09	BB	2,2325
5	4,017	342,32	168,01	0,03	0,03	BV	2,0374
6	4,079	5905,15	2759,08	0,45	0,45	VB	2,1403
7	13,716	7370,77	1757,49	0,56	0,56	BB	4,1939
		1304585,89	567678,66	100,00	100,00		

Missing Component Report

Component	Expected Retention (Calibration File)

All components were found

2.3 Table 1, Entry #3

Figure S27: ^{1}H NMR spectra of Benzophenone.

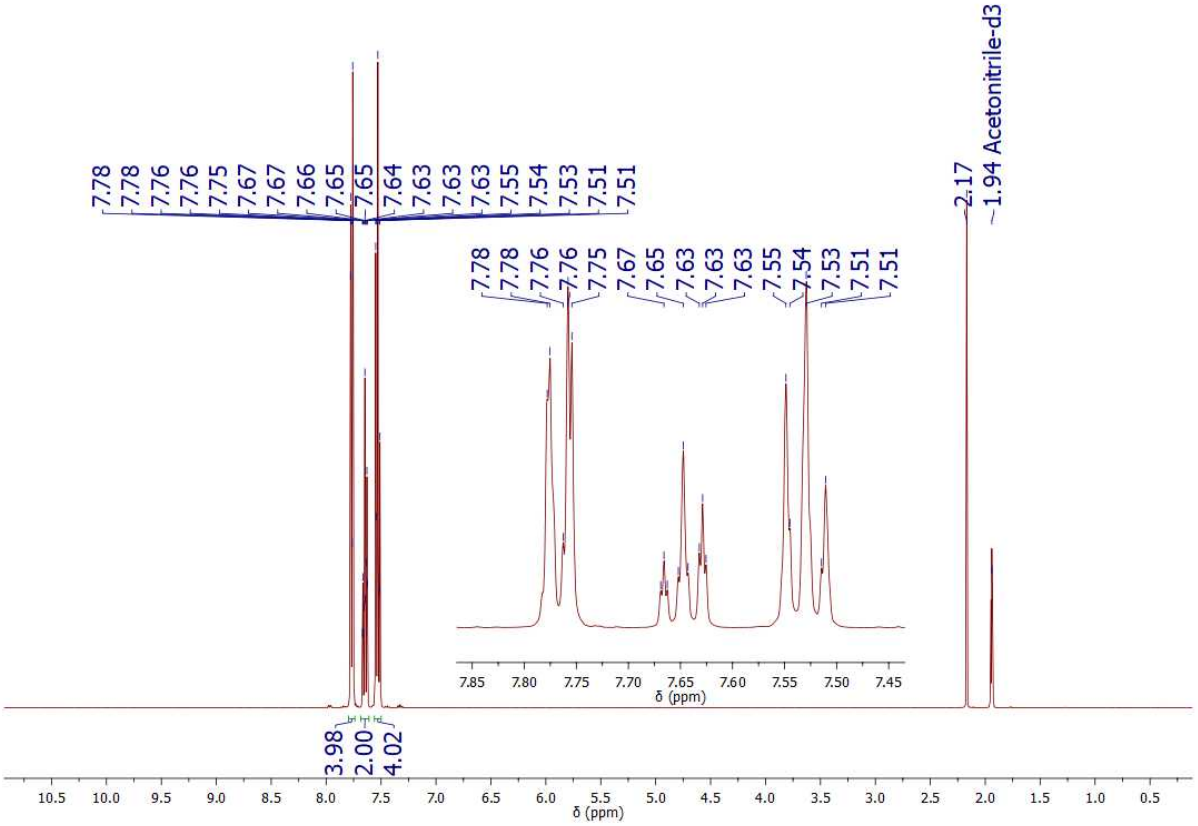

Figure S28: ^{1}H NMR spectra of Benzhydrol (Diphenylmethanol).

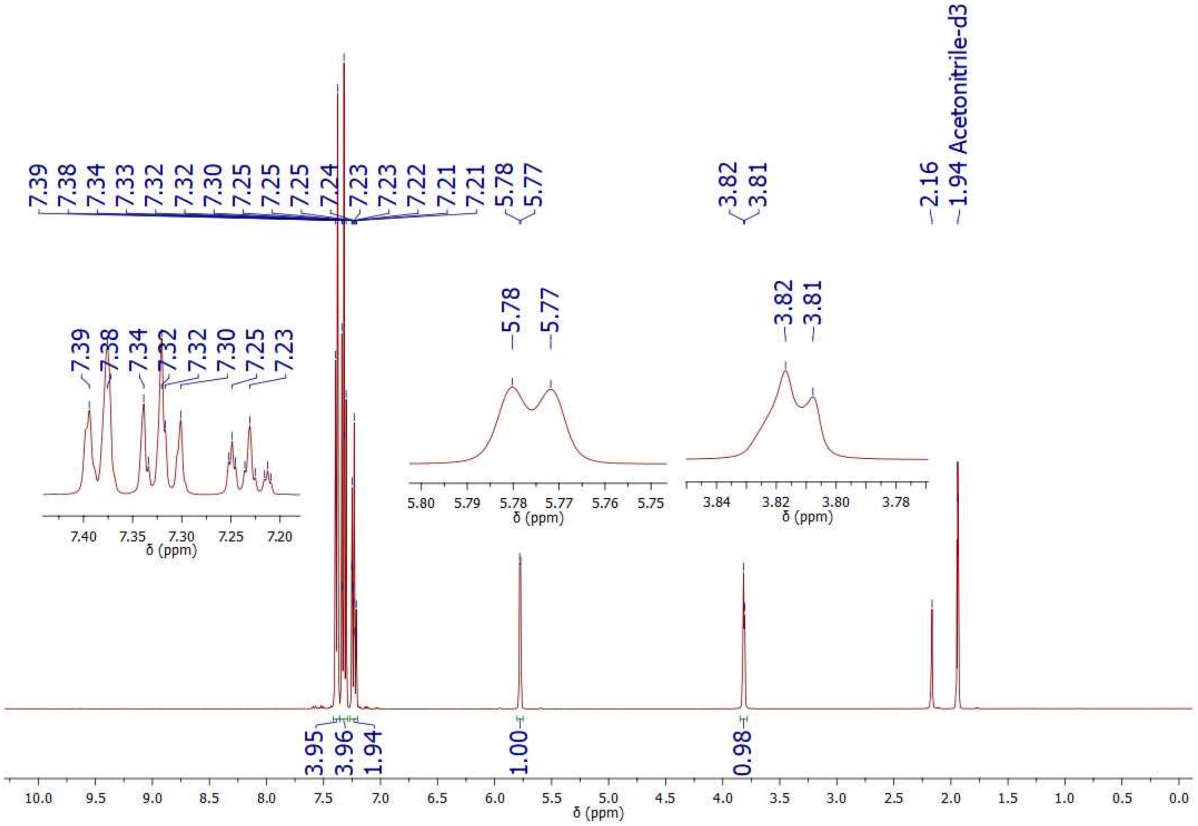

Figure S29: ^{1}H NMR spectra of catalysis #3 after 15 min.

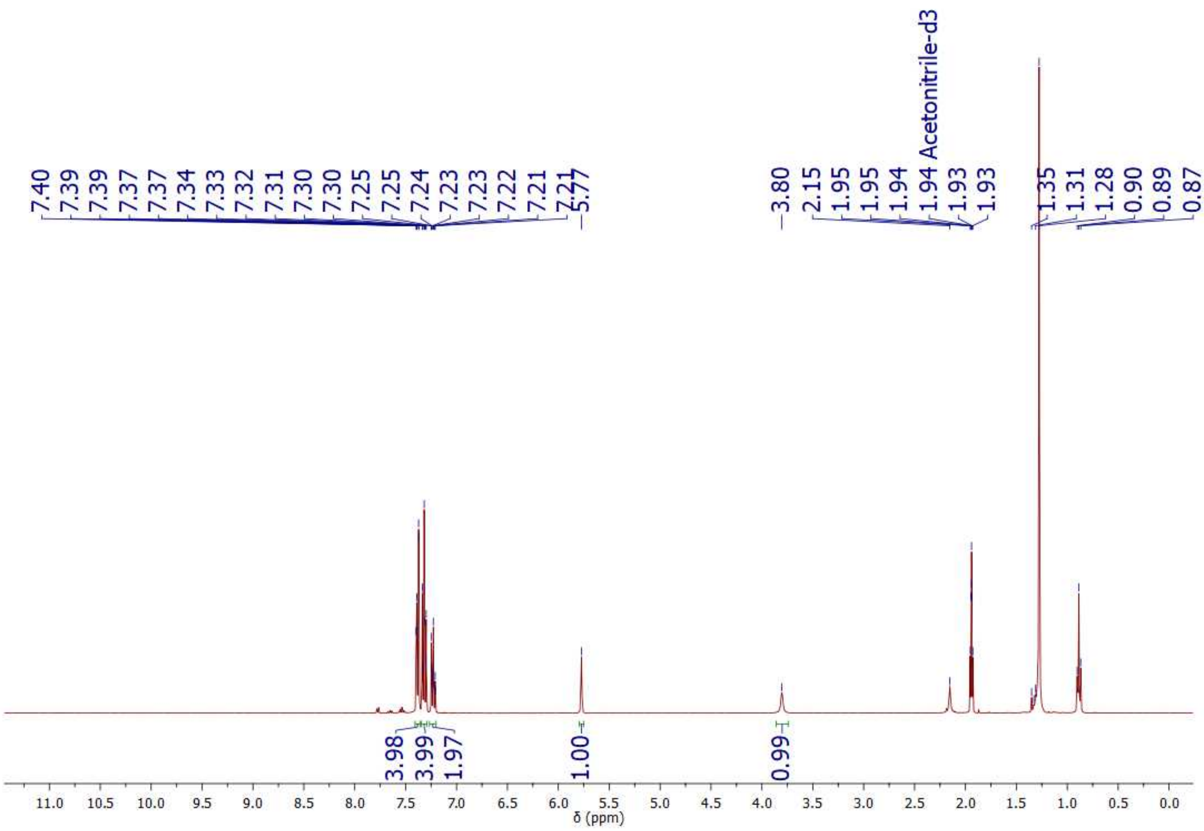

Figure S30: Determination of the yield of #3 via NMR spectroscopy after 15 min.

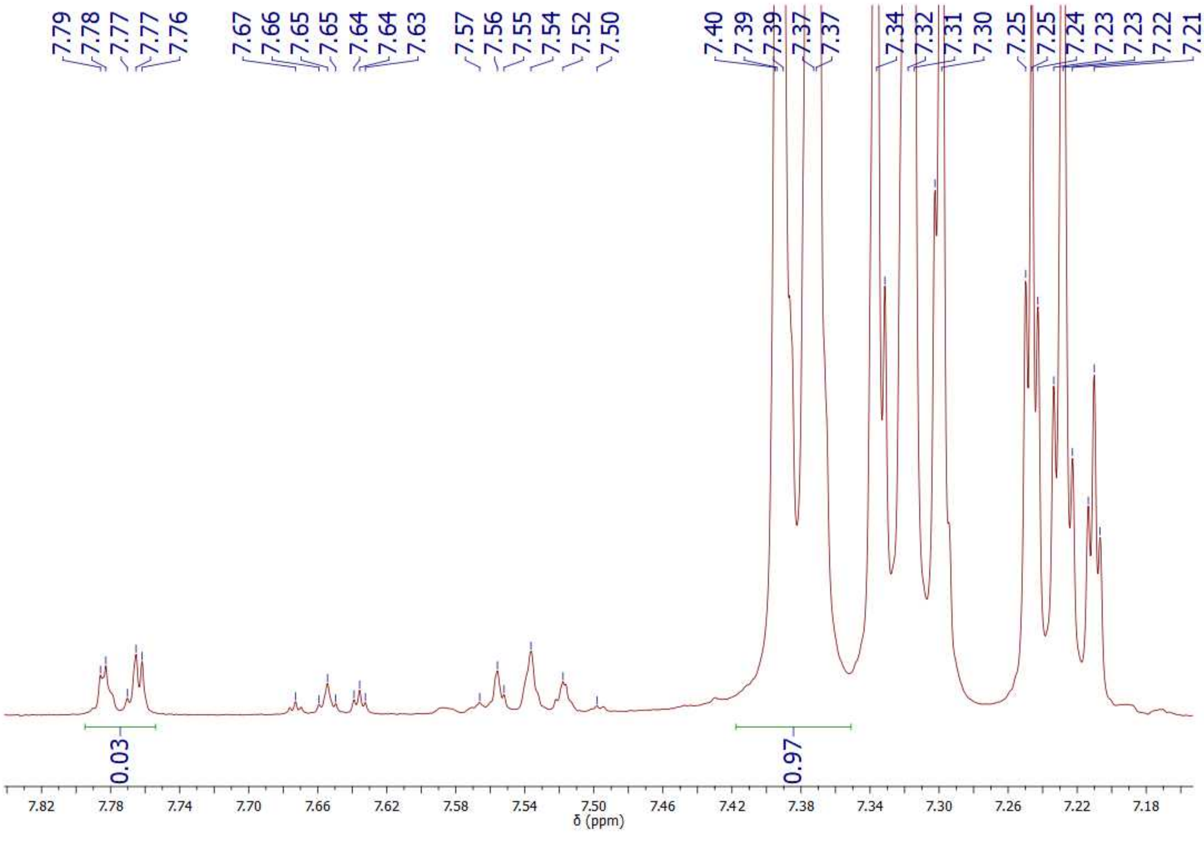

Figure S31: ^{1}H NMR spectra of catalysis #3 by time elapsed.

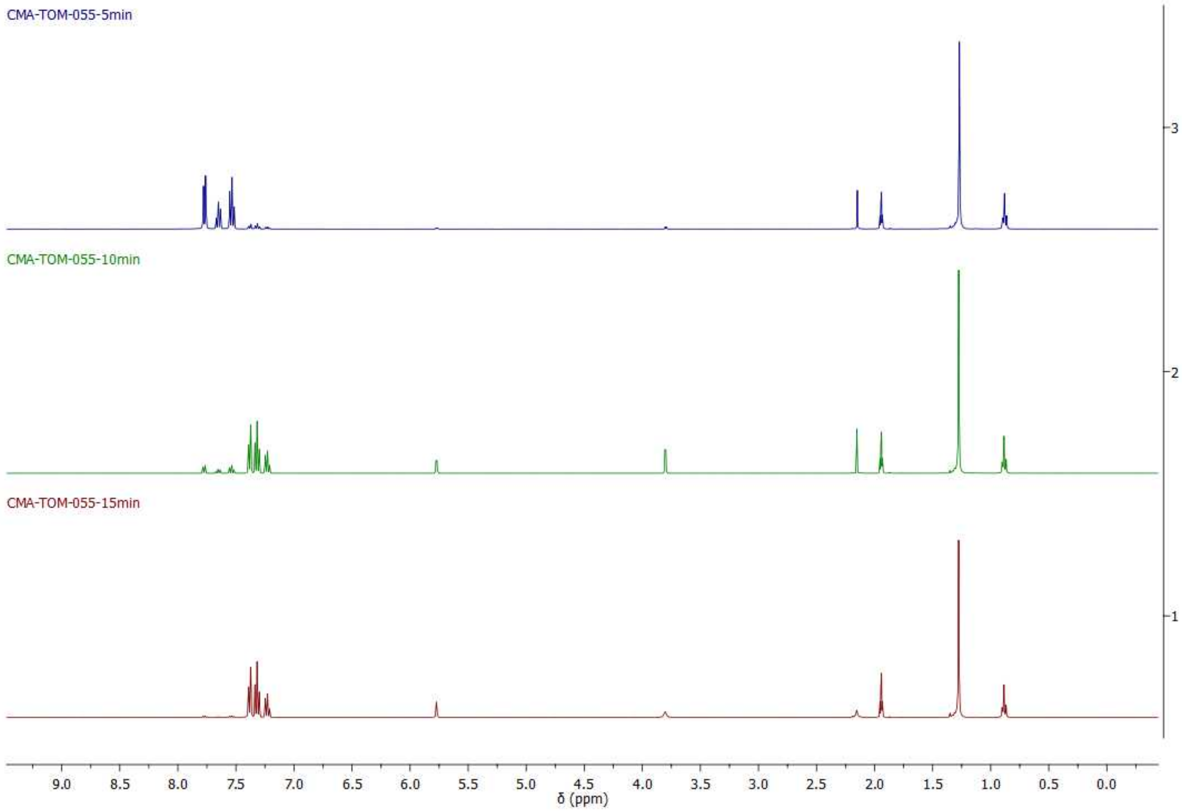

2.4 Table 1, Entry #4

Figure S32: ^{1}H NMR spectra of 2-Acetonaphthone.

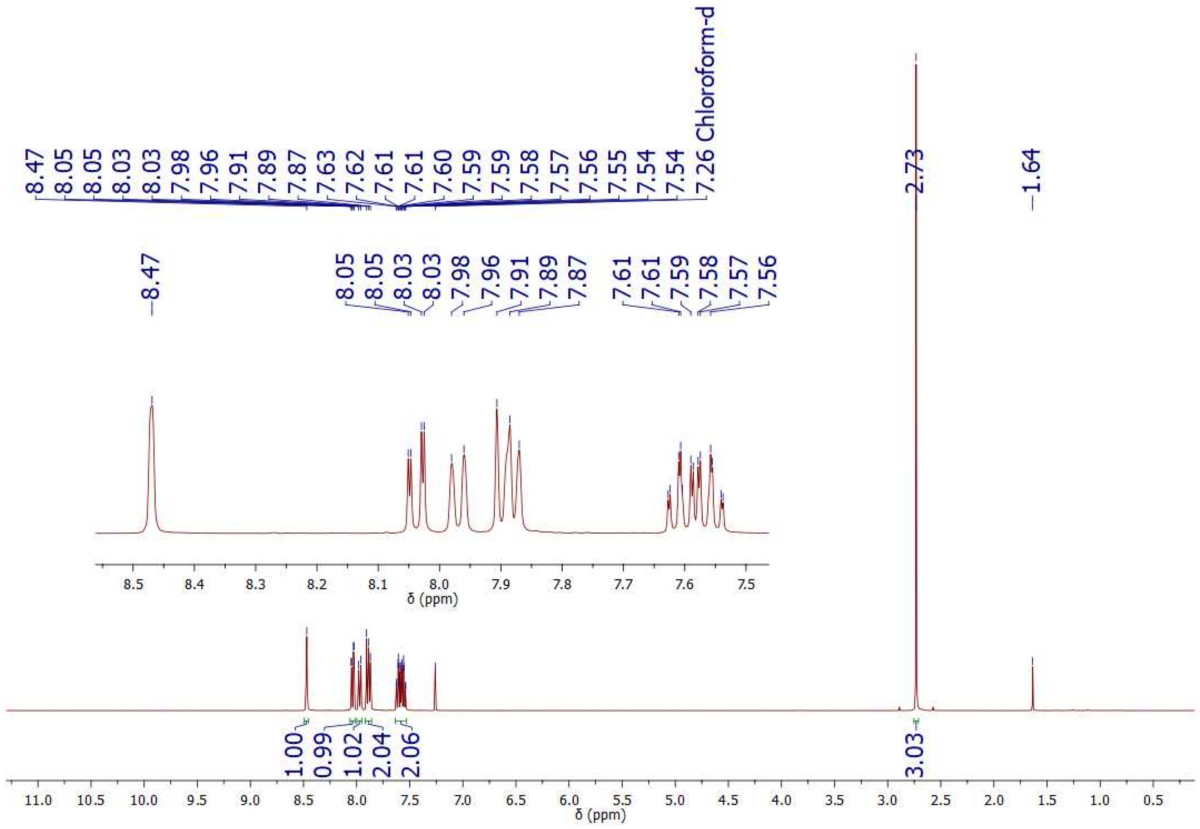

Figure S33: ^{1}H NMR spectra of 1-(2-Naphthyl)ethanol.

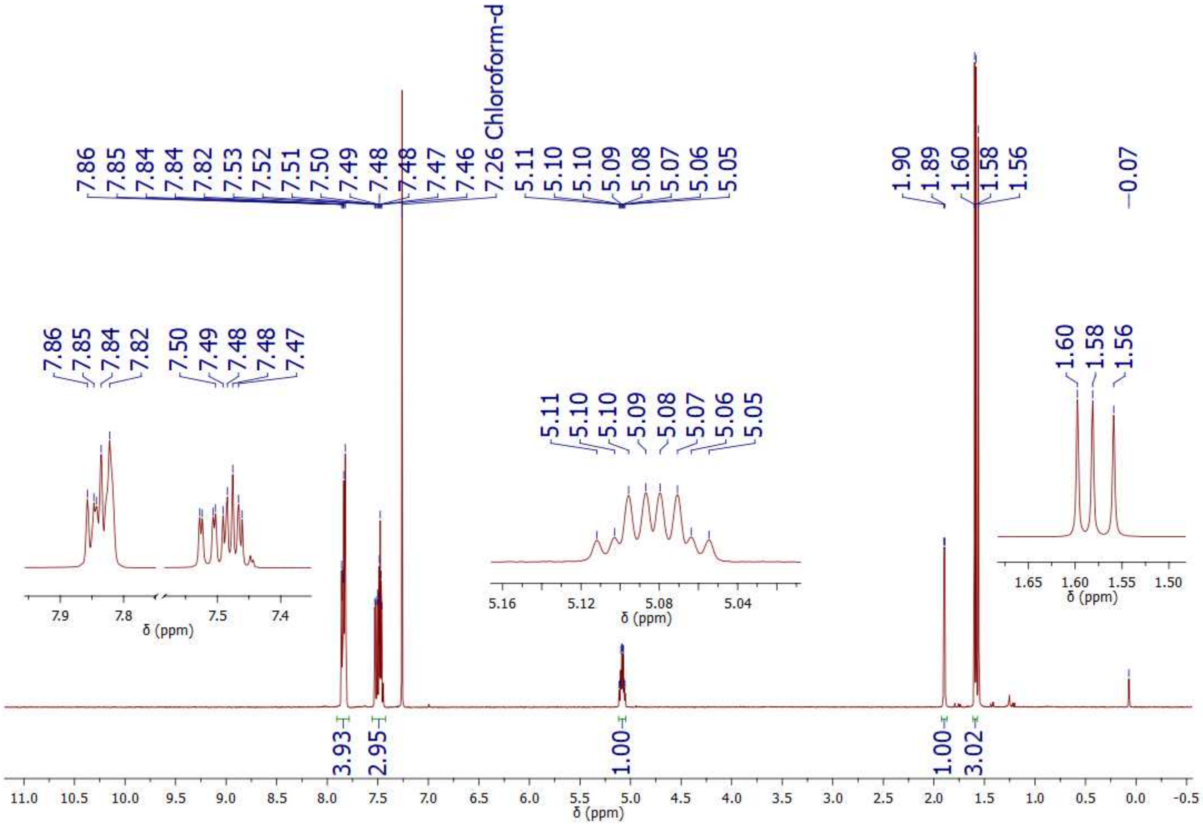

Figure S34: ^{1}H NMR spectra of catalysis #4 after 30 min.

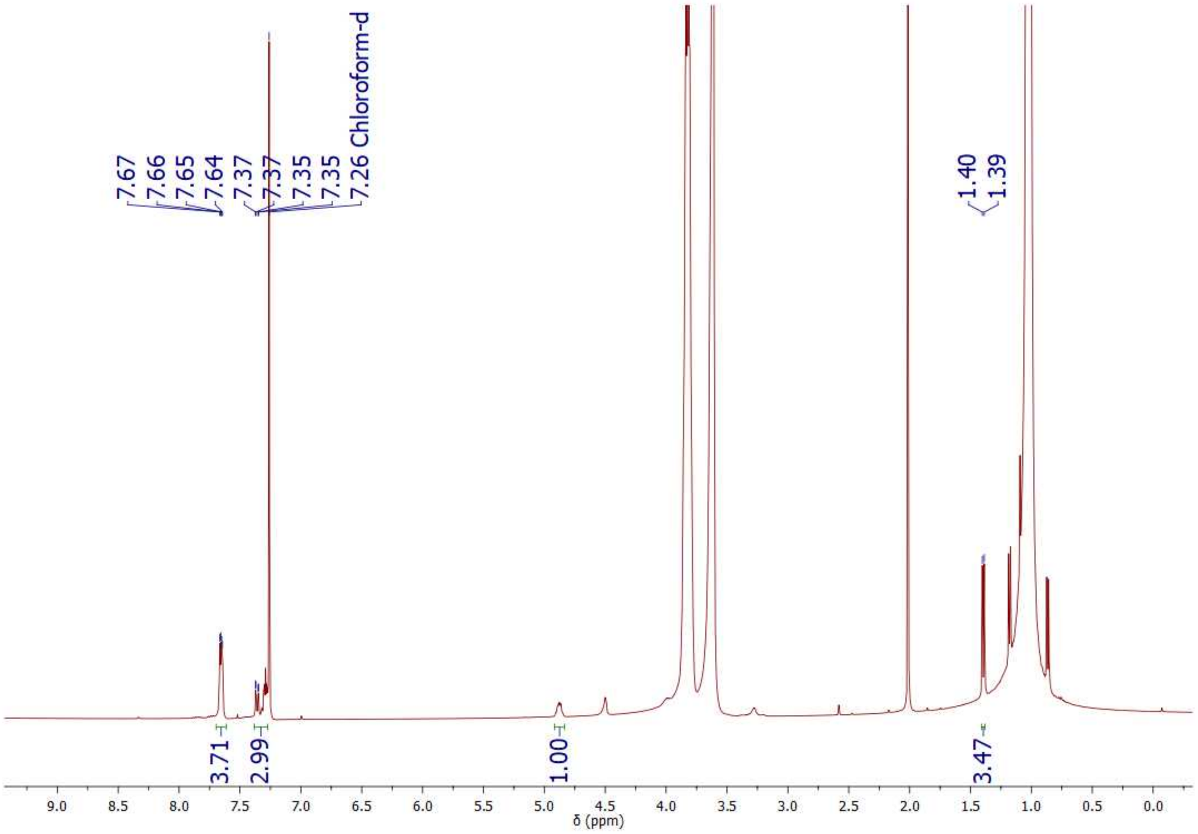

Figure S35: Determination of the yield of #4 via NMR spectroscopy after 30 min.

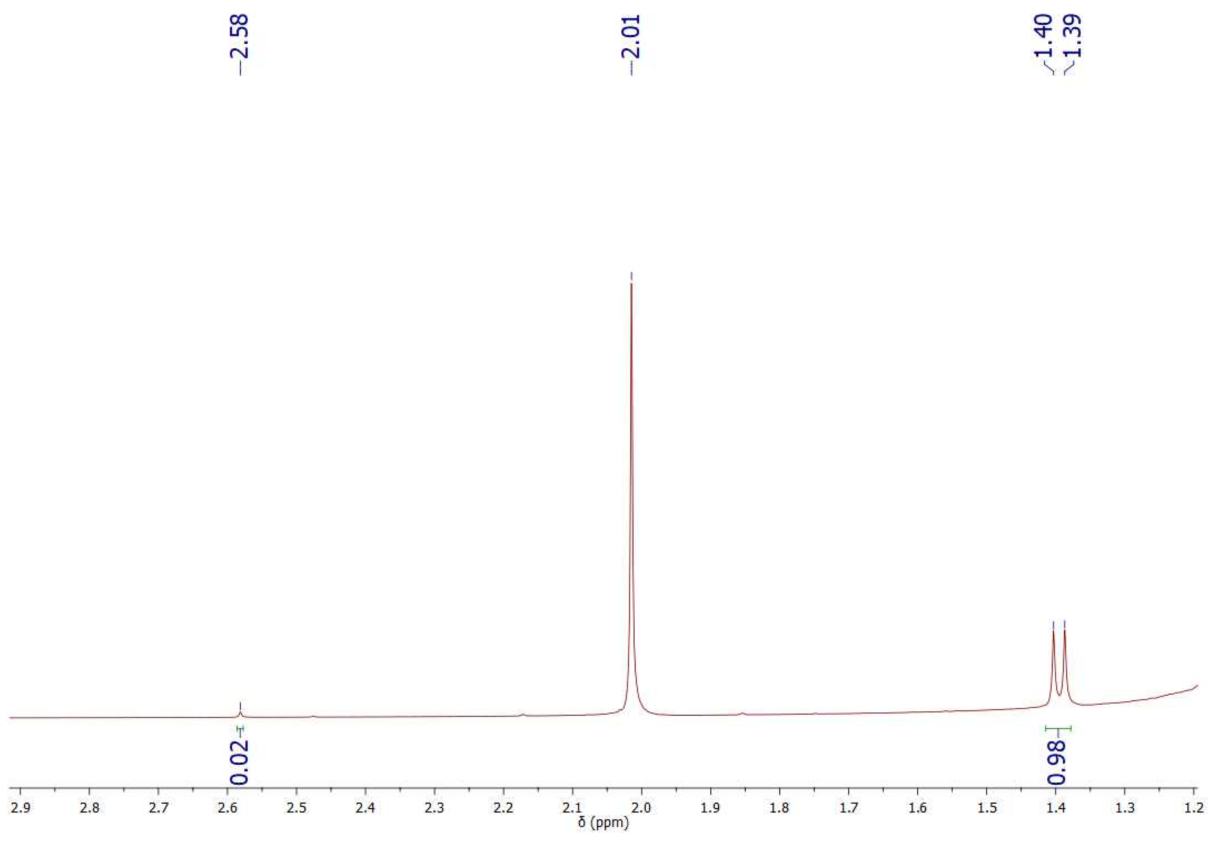

Figure S36: ^{1}H NMR spectra of catalysis #4 by time elapsed.

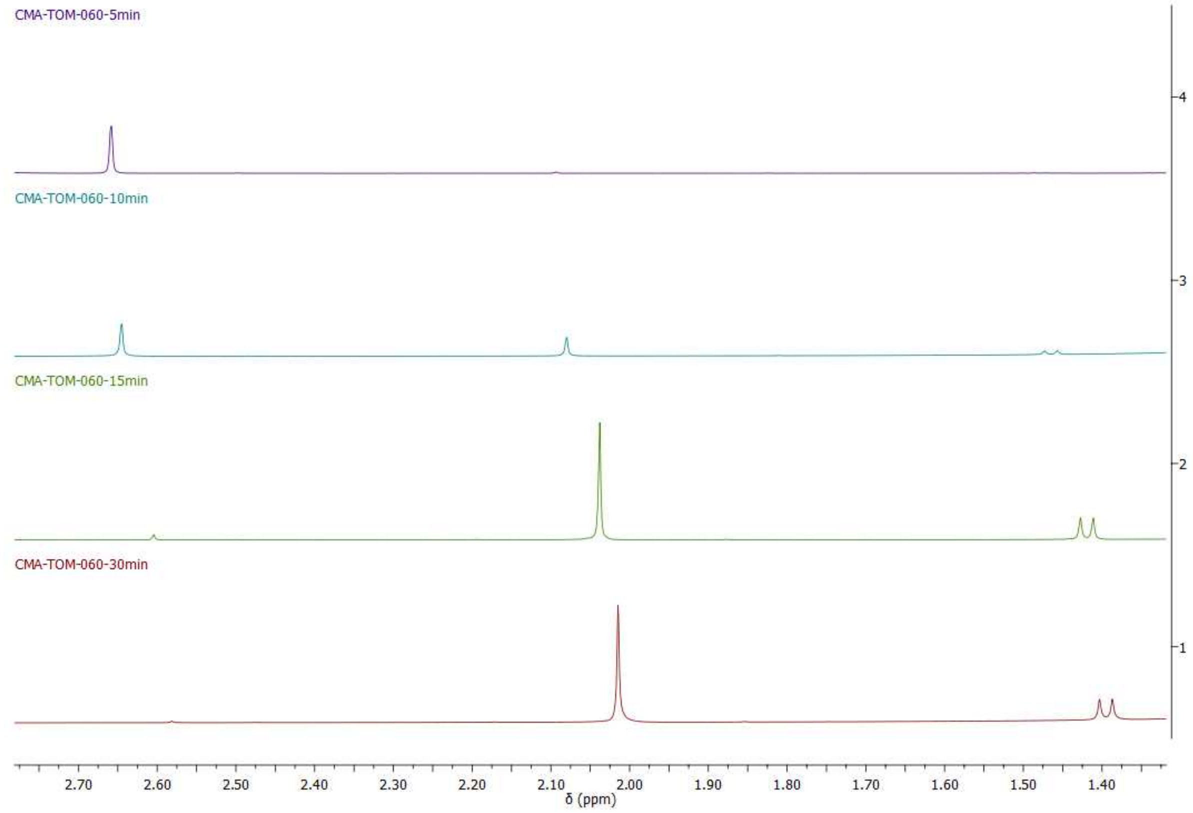

2.5 Table 1, Entry #5

Figure S37: GC chromatogram of 4-Methylacetophenone.

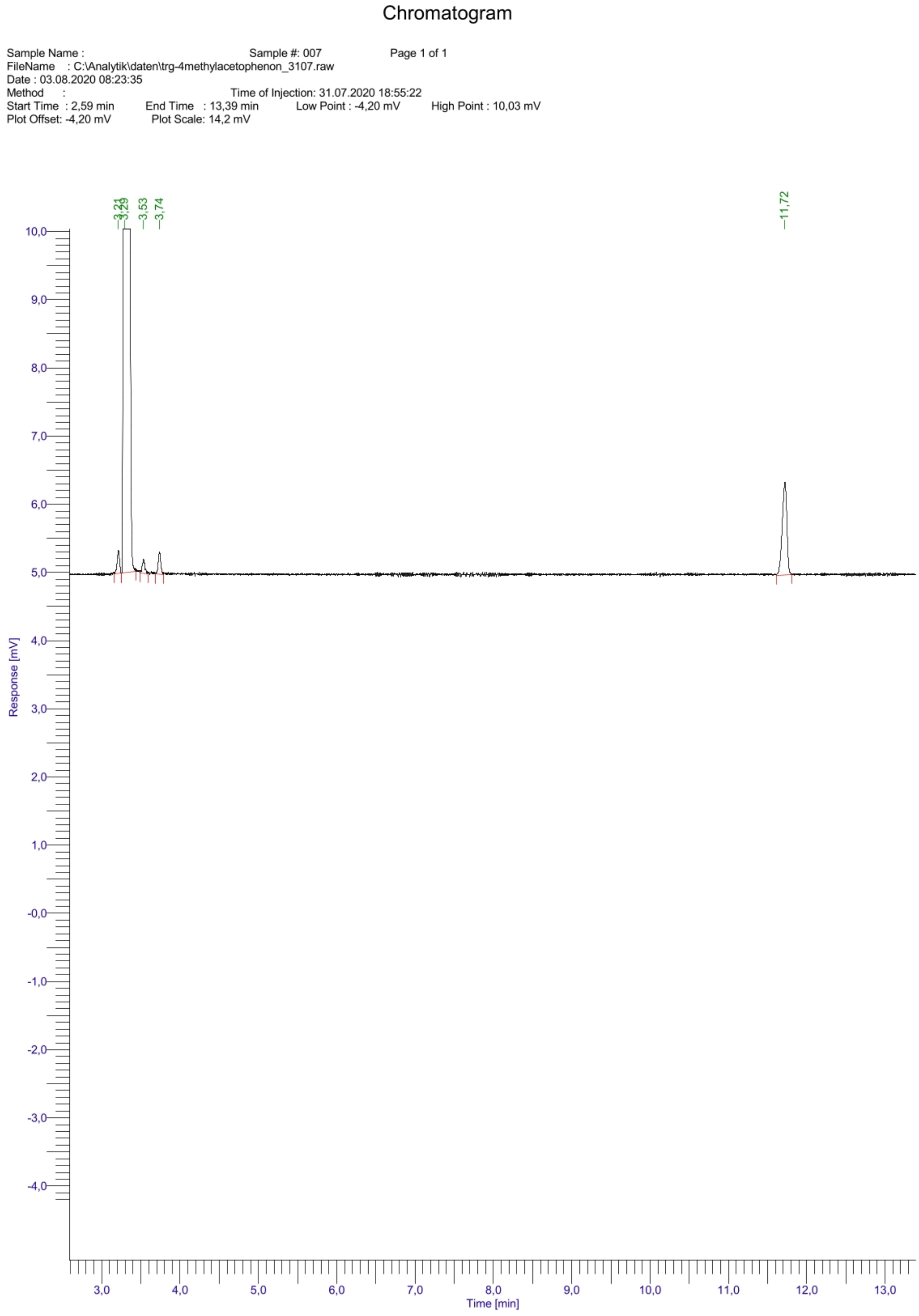

Software Version	: 6.3.2.0646		Date	: 03.08.2020 08:22:36
Operator	: Messaccount		Sample Name	:
Sample Number	: 007		Study	:
AutoSampler	: BUILT-IN		Rack/Vial	: 0/7
Instrument Name	: GC Clarus 580		Channel	: A
Instrument Serial #	: 580S12092501		A/D mV Range	: 1000
Delay Time	: 0,00 min		End Time	: 20,00 min
Sampling Rate	: 12,5000 pts/s			
Sample Volume	: 1,000000 ul			
Sample Amount	: 1,0000		Area Reject	: 0,000000
Data Acquisition Time	: 31.07.2020 18:55:22		Dilution Factor	: 1,00
			Cycle	: 7

Raw Data File : C:\Analytik\daten\trg-4methylacetophenon_3107.raw
Result File : C:\Analytik\daten\trg-4methylacetophenon_3107-20200803-081326.rst [Editing in Progress]
Inst Method : C:\Analytik\methoden\STANDARD_4K_aufheizrate from
C:\Analytik\daten\trg-4methylacetophenon_3107.raw
Proc Method : C:\Analytik\methoden\STANDARD_4K_aufheizrate.mth from
C:\Analytik\daten\trg-4methylacetophenon_3107-20200803-081326.rst [Editing in Progress]
Calib Method : C:\Analytik\methoden\STANDARD_4K_aufheizrate.mth from
C:\Analytik\daten\trg-4methylacetophenon_3107-20200803-081326.rst [Editing in Progress]
Report Format File: C:\Analytik\methoden\STANDARD_4K_aufheizrate.rpt
Sequence File : C:\Analytik\sequenzen\trg231072020.seq

DEFAULT REPORT

Peak #	Time [min]	Area [µV·s]	Height [µV]	Area [%]	Norm. Area [%]	BL	Area/Height [s]
1	3,212	732,72	338,40	0,05	0,05	BB	2,1653
2	3,288	1347159,65	558127,56	99,45	99,45	BB	2,4137
3	3,531	469,74	206,53	0,03	0,03	BB	2,2745
4	3,739	736,93	322,42	0,05	0,05	BB	2,2856
5	11,719	5573,95	1357,43	0,41	0,41	BB	4,1063
		1354672,98	560352,33	100,00	100,00		

Missing Component Report

Component	Expected Retention (Calibration File)

All components were found

Figure S38: GC chromatogram of 1-(4-Methylphenyl)ethanol.

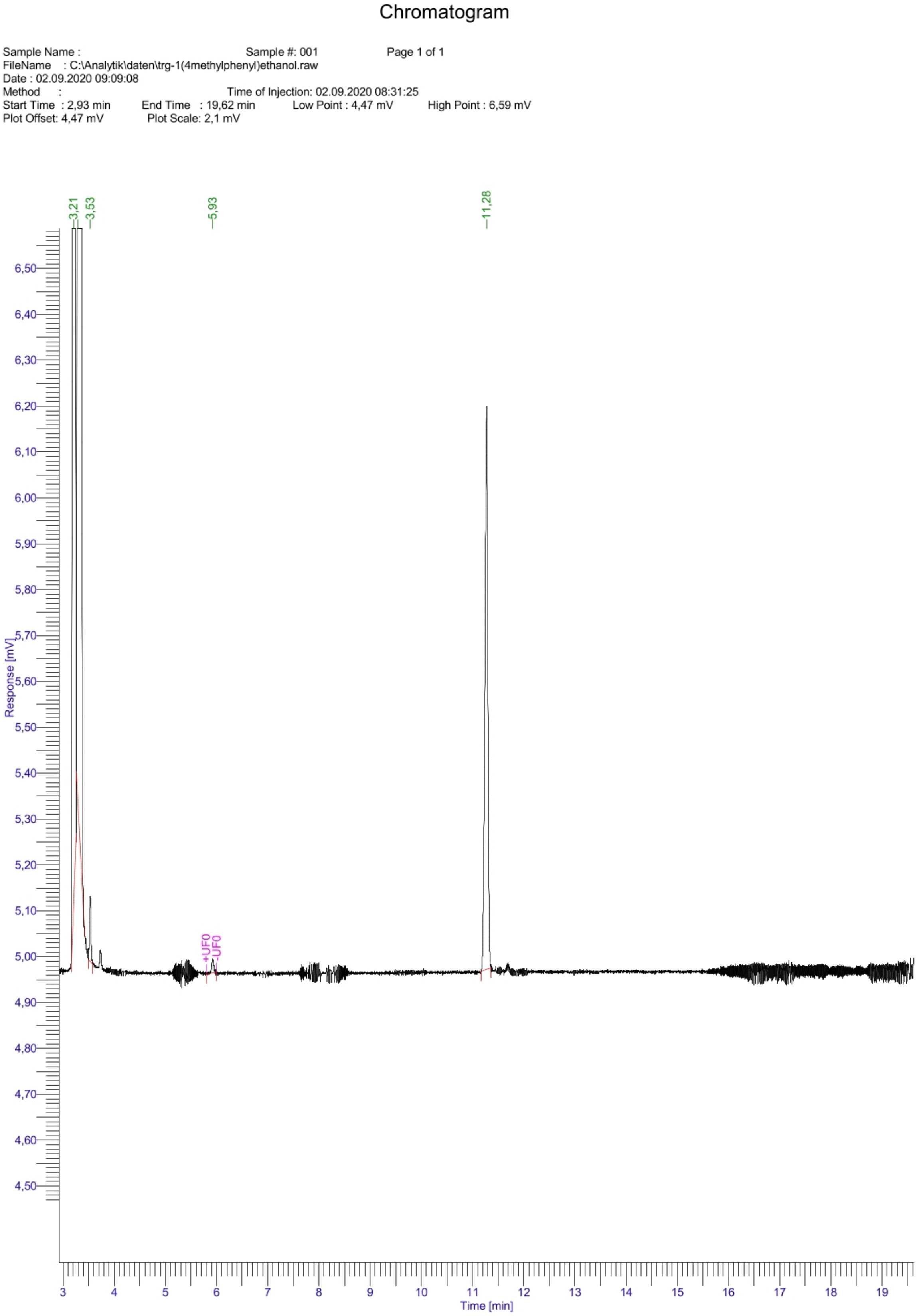

Software Version	: 6.3.2.0646		Date	: 02.09.2020 09:06:28
Operator	: Messaccount		Sample Name	:
Sample Number	: 001		Study	:
AutoSampler	: BUILT-IN		Rack/Vial	: 0/28
Instrument Name	: GC Clarus 580		Channel	: A
Instrument Serial #	: 580S12092501		A/D mV Range	: 1000
Delay Time	: 0,00 min		End Time	: 20,00 min
Sampling Rate	: 12,5000 pts/s			
Sample Volume	: 1,000000 ul			
Sample Amount	: 1,0000		Area Reject	: 0,000000
Data Acquisition Time	: 02.09.2020 08:31:25		Dilution Factor	: 1,00
			Cycle	: 1

Raw Data File : C:\Analytik\daten\trg-1(4methylphenyl)ethanol.raw
Result File : C:\Analytik\daten\trg-1(4methylphenyl)ethanol-20200902-090523.rst [Editing in Progress]
Inst Method : C:\Analytik\methoden\STANDARD_4K_aufheizrate from
C:\Analytik\daten\trg-1(4methylphenyl)ethanol.raw
Proc Method : C:\Analytik\methoden\STANDARD_4K_aufheizrate.mth from
C:\Analytik\daten\trg-1(4methylphenyl)ethanol-20200902-090523.rst [Editing in Progress]
Calib Method : C:\Analytik\methoden\STANDARD_4K_aufheizrate.mth from
C:\Analytik\daten\trg-1(4methylphenyl)ethanol-20200902-090523.rst [Editing in Progress]
Report Format File: C:\Analytik\methoden\STANDARD_4K_aufheizrate.rpt
Sequence File : C:\Analytik\sequenzen\trg02092020.seq

DEFAULT REPORT

Peak #	Time [min]	Area [µV·s]	Height [µV]	Area [%]	Norm. Area [%]	BL	Area/Height [s]
1	3,210	119682,98	58709,51	9,08	9,08	BB	2,0386
2	3,291	1193011,96	506841,46	90,52	90,52	BB	2,3538
3	3,534	311,08	142,99	0,02	0,02	BB	2,1754
4	5,927	82,68	29,74	0,01	0,01	MM	2,7799
5	11,279	4805,55	1227,45	0,36	0,36	BB	3,9151
		1317894,25	566951,15	100,00	100,00		

Missing Component Report

Component	Expected Retention (Calibration File)

All components were found

Figure S39: GC chromatogram of catalysis #5 after 30 min.

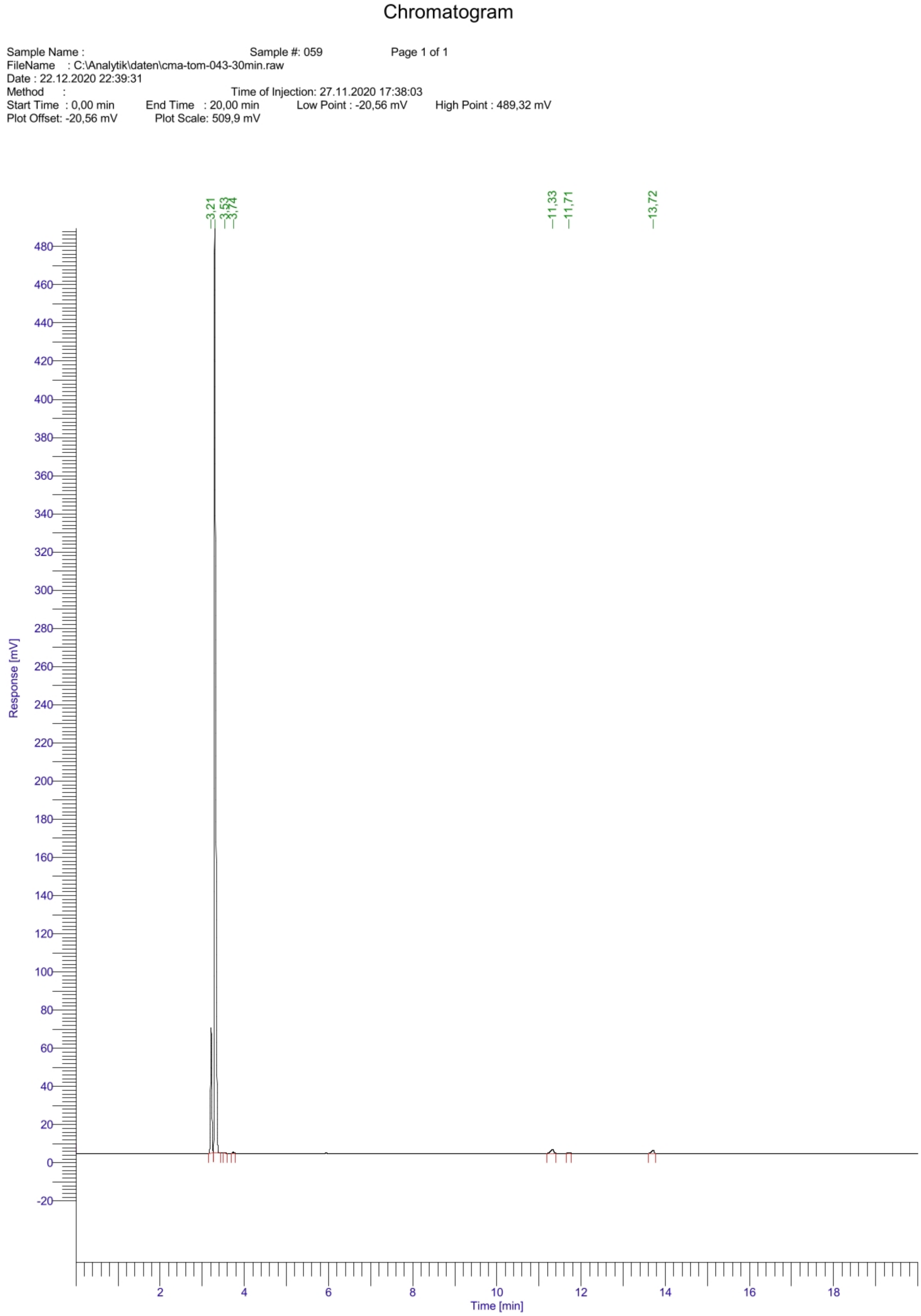

Software Version	: 6.3.2.0646	Date	: 30.11.2020 08:51:57
Operator	: Messaccount	Sample Name	:
Sample Number	: 059	Study	:
AutoSampler	: BUILT-IN	Rack/Vial	: 0/59
Instrument Name	: GC Clarus 580	Channel	: A
Instrument Serial #	: None	A/D mV Range	: 1000
Delay Time	: 0,00 min	End Time	: 20,00 min
Sampling Rate	: 12,5000 pts/s		
Sample Volume	: 1,000000 ul		
Sample Amount	: 1,0000	Area Reject	: 0,000000
Data Acquisition Time	: 27.11.2020 17:38:03	Dilution Factor	: 1,00
		Cycle	: 59

Raw Data File : C:\Analytik\daten\cma-tom-043-30min.raw
Result File : C:\Analytik\daten\cma-tom-043-30min-20201130-084826.rst [Editing in Progress]
Inst Method : C:\Analytik\methoden\STANDARD_4K_aufheizrate from
C:\Analytik\daten\cma-tom-043-30min.raw
Proc Method : C:\Analytik\methoden\STANDARD_4K_aufheizrate.mth from
C:\Analytik\daten\cma-tom-043-30min-20201130-084826.rst [Editing in Progress]
Calib Method : C:\Analytik\methoden\STANDARD_4K_aufheizrate.mth from
C:\Analytik\daten\cma-tom-043-30min-20201130-084826.rst [Editing in Progress]
Report Format File: C:\Analytik\methoden\STANDARD_4K_aufheizrate.rpt
Sequence File : C:\Analytik\sequenzen\nncmars26112020.seq

DEFAULT REPORT

Peak #	Time [min]	Area [µV·s]	Height [µV]	Area [%]	Norm. Area [%]	BL	Area/Height [s]
1	3,210	134786,18	65619,19	10,51	10,51	BB	2,0541
2	3,293	1129062,60	484099,35	88,02	88,02	BB	2,3323
3	3,532	278,90	140,17	0,02	0,02	BB	1,9897
4	3,737	1235,77	571,47	0,10	0,10	BB	2,1624
5	11,326	9695,96	2120,02	0,76	0,76	BB	4,5735
6	11,709	815,74	232,70	0,06	0,06	BB	3,5056
7	13,715	6858,00	1655,44	0,53	0,53	BB	4,1427
		1282733,14	554438,33	100,00	100,00		

Missing Component Report

Component	Expected Retention (Calibration File)

All components were found

2.6 Table 1, Entry #6

Figure S40: GC chromatogram of 4-Methoxyacetophenone.

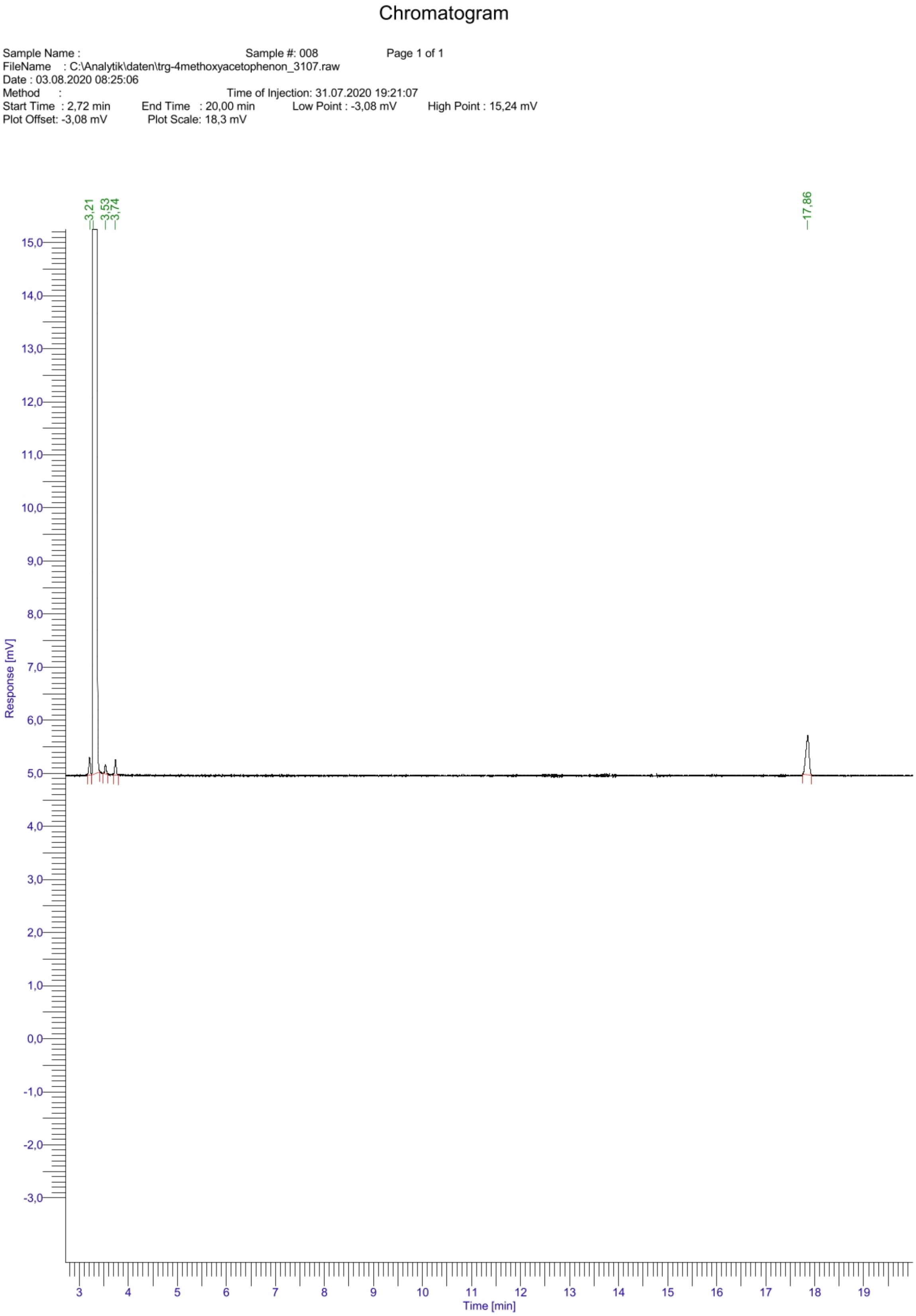

Software Version	: 6.3.2.0646		Date	: 03.08.2020 08:23:59
Operator	: Messaccount		Sample Name	:
Sample Number	: 008		Study	:
AutoSampler	: BUILT-IN		Rack/Vial	: 0/8
Instrument Name	: GC Clarus 580		Channel	: A
Instrument Serial #	: 580S12092501		A/D mV Range	: 1000
Delay Time	: 0,00 min		End Time	: 20,00 min
Sampling Rate	: 12,5000 pts/s			
Sample Volume	: 1,000000 ul			
Sample Amount	: 1,0000		Area Reject	: 0,000000
Data Acquisition Time	: 31.07.2020 19:21:07		Dilution Factor	: 1,00
			Cycle	: 8

Raw Data File : C:\Analytik\daten\trg-4methoxyacetophenon_3107.raw
Result File : C:\Analytik\daten\trg-4methoxyacetophenon_3107-20200803-081326.rst [Editing in Progress]
Inst Method : C:\Analytik\methoden\STANDARD_4K_aufheizrate from C:\Analytik\daten\trg-4methoxyacetophenon_3107.raw
Proc Method : C:\Analytik\methoden\STANDARD_4K_aufheizrate.mth from C:\Analytik\daten\trg-4methoxyacetophenon_3107-20200803-081326.rst [Editing in Progress]
Calib Method : C:\Analytik\methoden\STANDARD_4K_aufheizrate.mth from C:\Analytik\daten\trg-4methoxyacetophenon_3107-20200803-081326.rst [Editing in Progress]
Report Format File: C:\Analytik\methoden\STANDARD_4K_aufheizrate.rpt
Sequence File : C:\Analytik\sequenzen\trg231072020.seq

DEFAULT REPORT

Peak #	Time [min]	Area [µV·s]	Height [µV]	Area [%]	Norm. Area [%]	BL	Area/Height [s]
1	3,210	716,23	327,42	0,05	0,05	BB	2,1875
2	3,288	1334128,02	551397,25	99,61	99,61	BB	2,4195
3	3,534	350,88	171,74	0,03	0,03	BB	2,0431
4	3,737	672,39	300,09	0,05	0,05	BB	2,2406
5	17,857	3466,00	747,23	0,26	0,26	BB	4,6384
		1339333,51	552943,73	100,00	100,00		

Missing Component Report

Component	Expected Retention (Calibration File)

All components were found

Figure S41: GC chromatogram of 1-(4-Methoxyphenyl)ethanol.

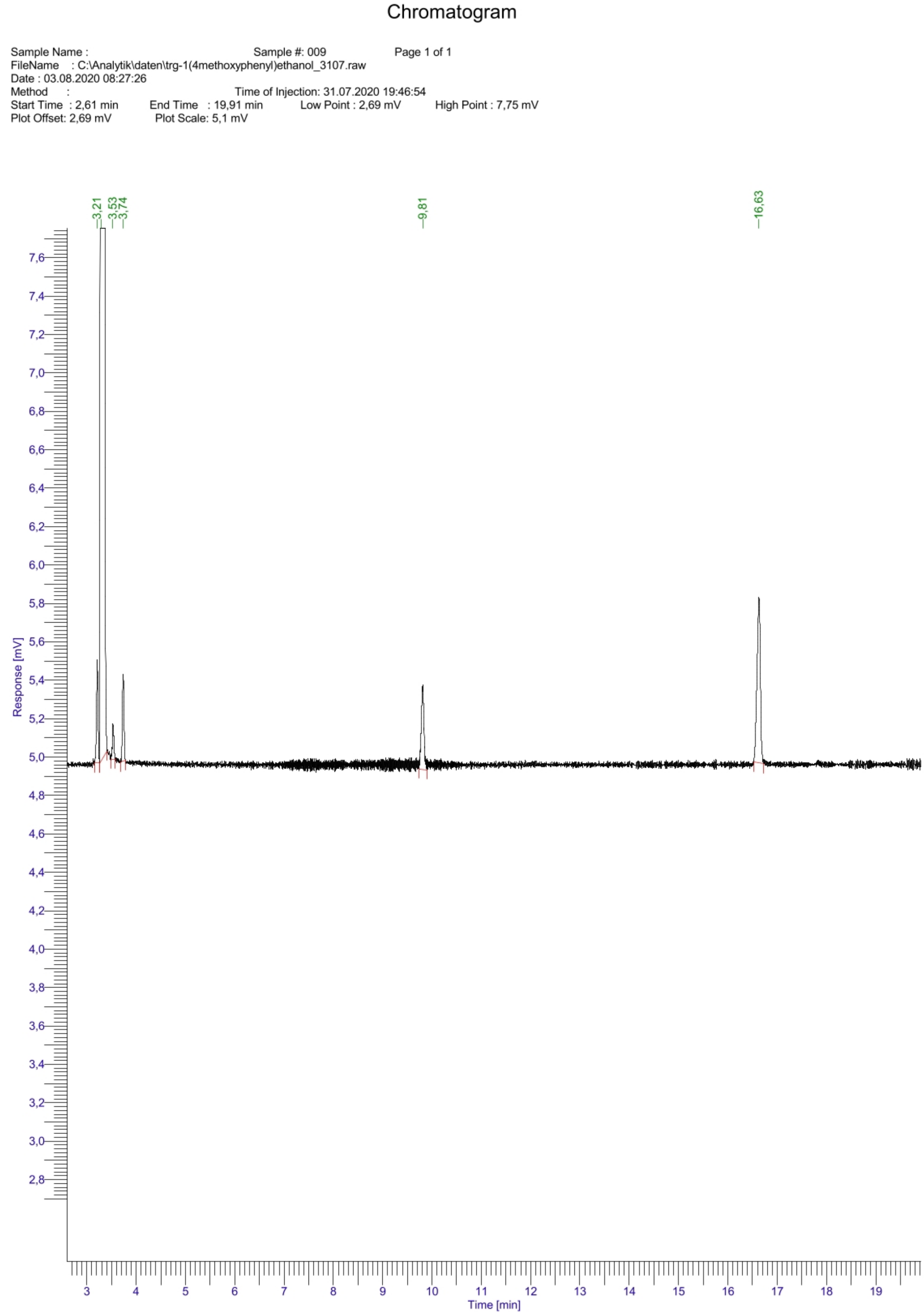

Software Version	: 6.3.2.0646		Date	: 03.08.2020 08:26:02
Operator	: Messaccount		Sample Name	:
Sample Number	: 009		Study	:
AutoSampler	: BUILT-IN		Rack/Vial	: 0/9
Instrument Name	: GC Clarus 580		Channel	: A
Instrument Serial #	: 580S12092501		A/D mV Range	: 1000
Delay Time	: 0,00 min		End Time	: 20,00 min
Sampling Rate	: 12,5000 pts/s			
Sample Volume	: 1,000000 ul			
Sample Amount	: 1,0000		Area Reject	: 0,000000
Data Acquisition Time	: 31.07.2020 19:46:54		Dilution Factor	: 1,00
			Cycle	: 9

Raw Data File : C:\Analytik\daten\trg-1(4methoxyphenyl)ethanol_3107.raw
Result File : C:\Analytik\daten\trg-1(4methoxyphenyl)ethanol_3107-20200803-081327.rst [Editing in Progress]
Inst Method : C:\Analytik\methoden\STANDARD_4K_aufheizrate from C:\Analytik\daten\trg-1(4methoxyphenyl)ethanol_3107.raw
Proc Method : C:\Analytik\methoden\STANDARD_4K_aufheizrate.mth from C:\Analytik\daten\trg-1(4methoxyphenyl)ethanol_3107-20200803-081327.rst [Editing in Progress]
Calib Method : C:\Analytik\methoden\STANDARD_4K_aufheizrate.mth from C:\Analytik\daten\trg-1(4methoxyphenyl)ethanol_3107-20200803-081327.rst [Editing in Progress]
Report Format File: C:\Analytik\methoden\STANDARD_4K_aufheizrate.rpt
Sequence File : C:\Analytik\sequenzen\trg231072020.seq

DEFAULT REPORT

Peak #	Time [min]	Area [µV·s]	Height [µV]	Area [%]	Norm. Area [%]	BL	Area/Height [s]
1	3,211	1137,48	537,16	0,08	0,08	BB	2,1176
2	3,288	1349506,60	555254,62	99,39	99,39	BB	2,4304
3	3,529	384,87	184,29	0,03	0,03	BB	2,0883
4	3,736	986,18	452,42	0,07	0,07	BB	2,1798
5	9,814	1657,66	441,29	0,12	0,12	BB	3,7564
6	16,629	4100,99	861,23	0,30	0,30	BB	4,7618
		1357773,77	557731,02	100,00	100,00		

Missing Component Report

Component	Expected Retention (Calibration File)

All components were found

Figure S42: GC chromatogram of catalysis #6 after 30 min.

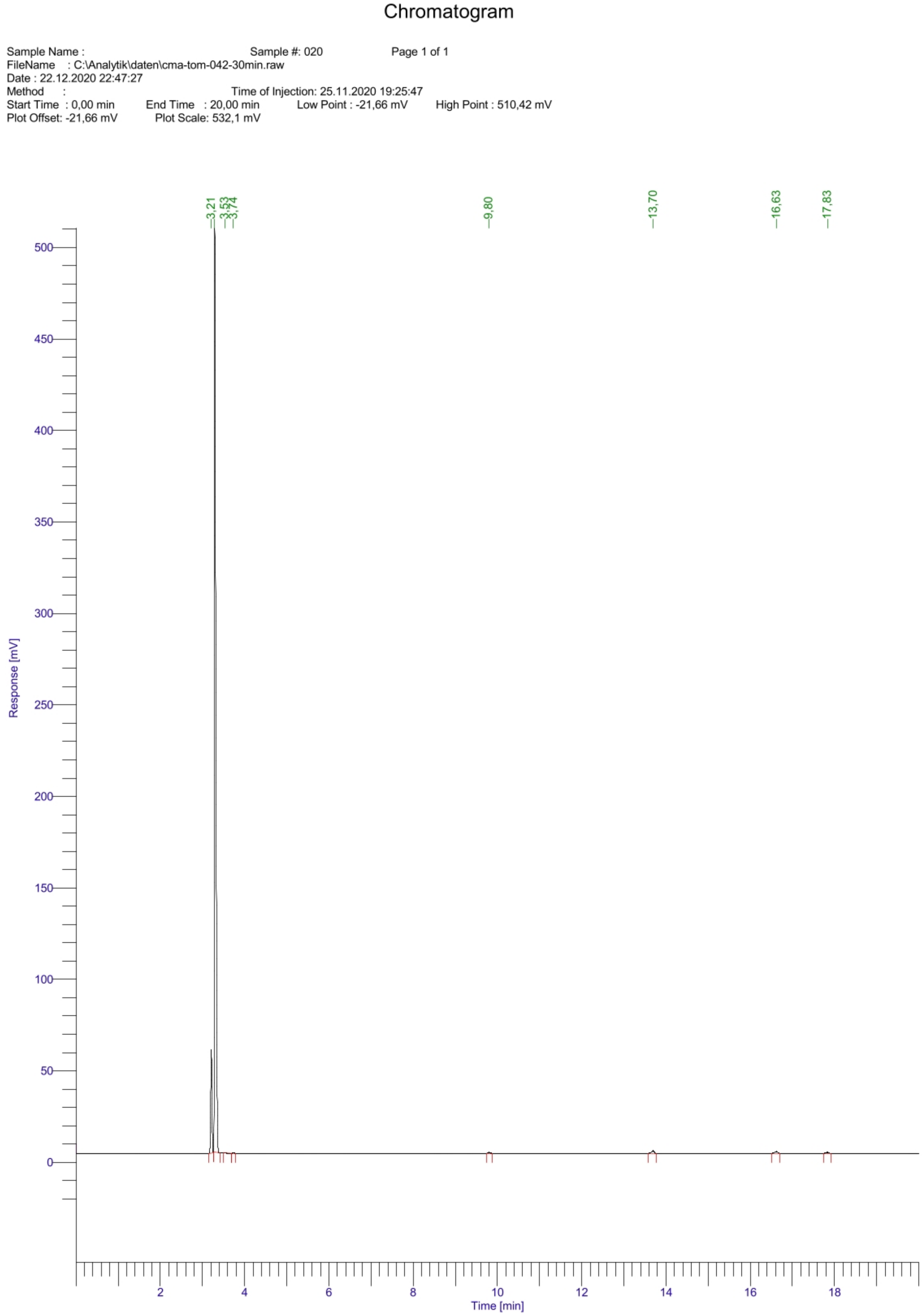

Software Version	: 6.3.2.0646		Date	: 26.11.2020 12:25:01
Operator	: Messaccount		Sample Name	:
Sample Number	: 020		Study	:
AutoSampler	: BUILT-IN		Rack/Vial	: 0/20
Instrument Name	: GC Clarus 580		Channel	: A
Instrument Serial #	: None		A/D mV Range	: 1000
Delay Time	: 0,00 min		End Time	: 20,00 min
Sampling Rate	: 12,5000 pts/s			
Sample Volume	: 1,000000 ul			
Sample Amount	: 1,0000		Area Reject	: 0,000000
Data Acquisition Time	: 25.11.2020 19:25:47		Dilution Factor	: 1,00
			Cycle	: 20

Raw Data File : C:\Analytik\daten\cma-tom-042-30min.raw
Result File : C:\Analytik\daten\cma-tom-042-30min-20201126-104620.rst [Editing in Progress]
Inst Method : C:\Analytik\methoden\STANDARD_4K_aufheizrate from
C:\Analytik\daten\cma-tom-042-30min.raw
Proc Method : C:\Analytik\methoden\STANDARD_4K_aufheizrate.mth from
C:\Analytik\daten\cma-tom-042-30min-20201126-104620.rst [Editing in Progress]
Calib Method : C:\Analytik\methoden\STANDARD_4K_aufheizrate.mth from
C:\Analytik\daten\cma-tom-042-30min-20201126-104620.rst [Editing in Progress]
Report Format File: C:\Analytik\methoden\STANDARD_4K_aufheizrate.rpt
Sequence File : C:\Analytik\sequenzen\rscmann25112020.seq

DEFAULT REPORT

Peak #	Time [min]	Area [µV·s]	Height [µV]	Area [%]	Norm. Area [%]	BL	Area/Height [s]
1	3,208	114572,48	56528,06	8,80	8,80	BB	2,0268
2	3,290	1172441,98	505283,79	90,04	90,04	BB	2,3204
3	3,527	312,45	153,11	0,02	0,02	BV	2,0407
4	3,736	906,84	407,17	0,07	0,07	VB	2,2272
5	9,797	1717,38	484,34	0,13	0,13	BB	3,5458
6	13,697	5639,08	1407,00	0,43	0,43	BB	4,0079
7	16,626	4448,49	923,08	0,34	0,34	BB	4,8192
8	17,833	2046,09	465,46	0,16	0,16	BB	4,3958
		1302084,78	565652,00	100,00	100,00		

Missing Component Report

Component	Expected Retention (Calibration File)

All components were found

2.7 Table 1, Entry #7

Figure S43: GC chromatogram of 4-Chloracetophenone.

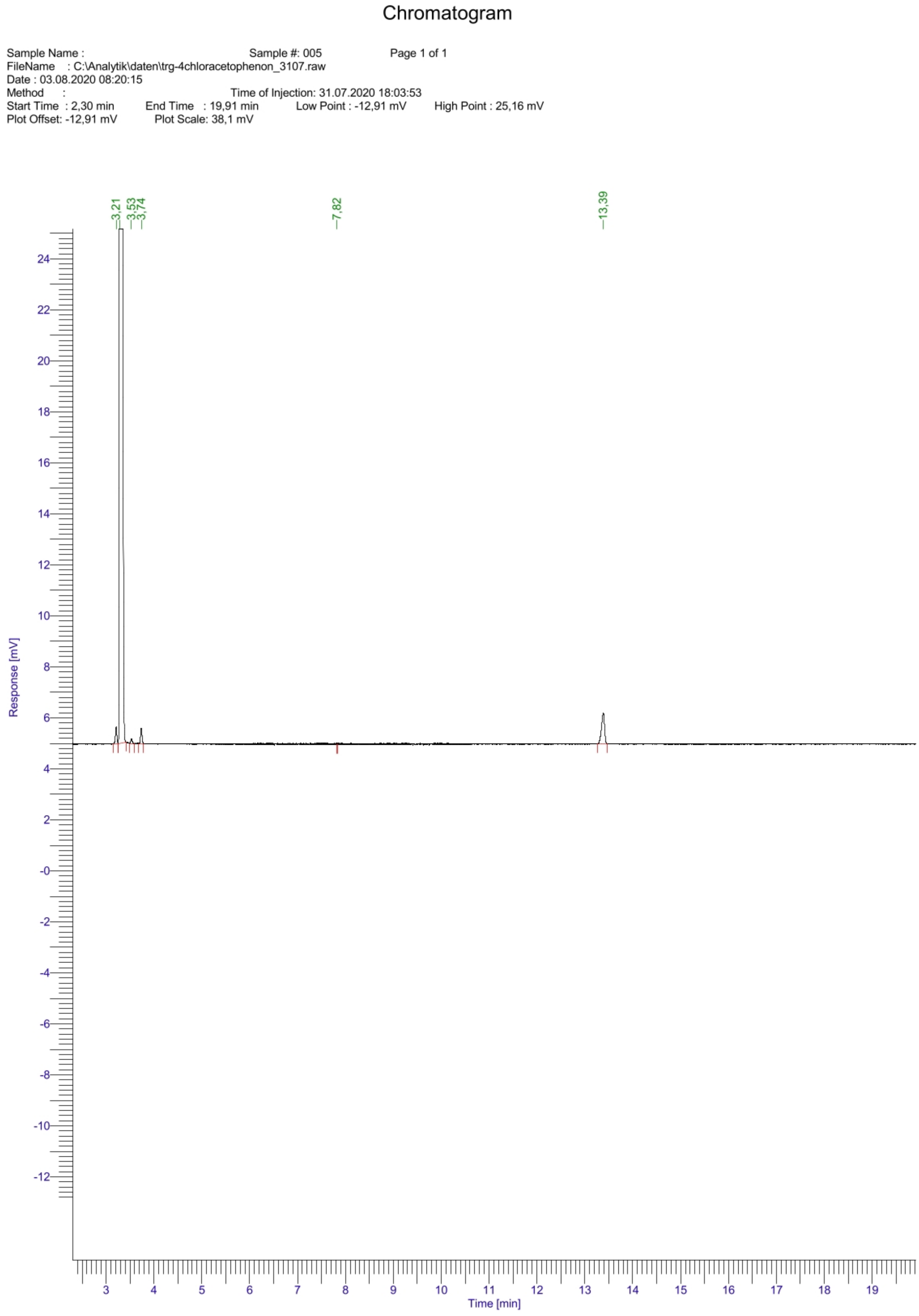

Software Version	:	6.3.2.0646	Date	:	03.08.2020 08:19:14
Operator	:	Messaccount	Sample Name	:	
Sample Number	:	005	Study	:	
AutoSampler	:	BUILT-IN	Rack/Vial	:	0/5
Instrument Name	:	GC Clarus 580	Channel	:	A
Instrument Serial #	:	580S12092501	A/D mV Range	:	1000
Delay Time	:	0,00 min	End Time	:	20,00 min
Sampling Rate	:	12,5000 pts/s			
Sample Volume	:	1,000000 ul			
Sample Amount	:	1,0000	Area Reject	:	0,000000
Data Acquisition Time	:	31.07.2020 18:03:53	Dilution Factor	:	1,00
			Cycle	:	5

Raw Data File : C:\Analytik\daten\trg-4chloracetophenon_3107.raw
Result File : C:\Analytik\daten\trg-4chloracetophenon_3107-20200803-081323.rst [Editing in Progress]
Inst Method : C:\Analytik\methoden\STANDARD_4K_aufheizrate from
C:\Analytik\daten\trg-4chloracetophenon_3107.raw
Proc Method : C:\Analytik\methoden\STANDARD_4K_aufheizrate.mth from
C:\Analytik\daten\trg-4chloracetophenon_3107-20200803-081323.rst [Editing in Progress]
Calib Method : C:\Analytik\methoden\STANDARD_4K_aufheizrate.mth from
C:\Analytik\daten\trg-4chloracetophenon_3107-20200803-081323.rst [Editing in Progress]
Report Format File: C:\Analytik\methoden\STANDARD_4K_aufheizrate.rpt
Sequence File : C:\Analytik\sequenzen\trg231072020.seq

DEFAULT REPORT

Peak #	Time [min]	Area [µV·s]	Height [µV]	Area [%]	Norm. Area [%]	BL	Area/Height [s]
1	3,212	1377,38	655,82	0,10	0,10	BB	2,1002
2	3,288	1342106,89	557881,50	99,37	99,37	BB	2,4057
3	3,530	460,58	190,84	0,03	0,03	BB	2,4135
4	3,736	1307,28	600,64	0,10	0,10	BB	2,1765
5	7,825	28,00	63,70	0,00	0,00	BB	0,4396
6	13,386	5360,38	1209,40	0,40	0,40	BB	4,4323
		1350640,51	560601,89	100,00	100,00		

Missing Component Report

Component	Expected Retention (Calibration File)

All components were found

Figure S44: GC chromatogram of 1-(4-Chlorphenyl)ethanol.

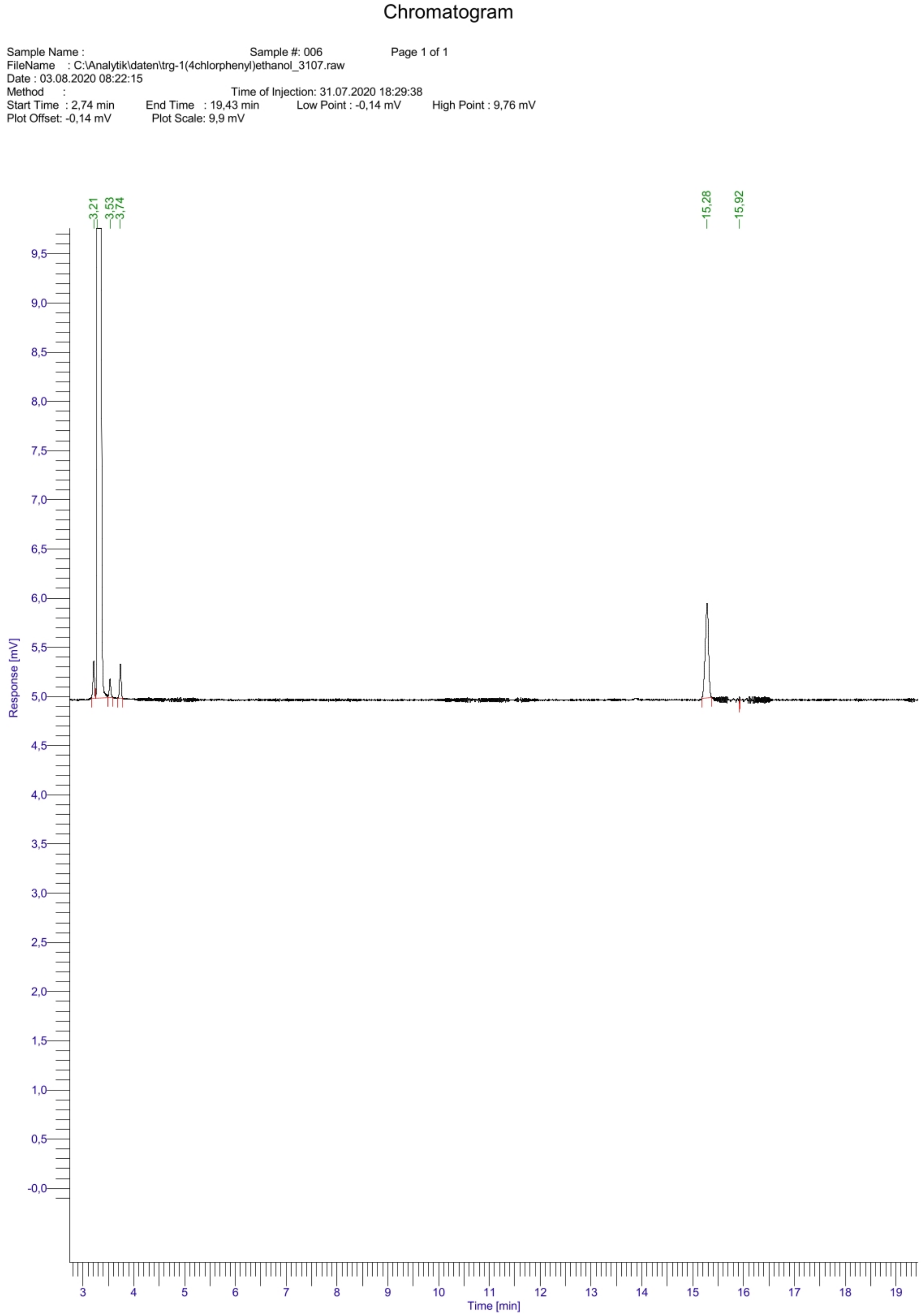

Software Version	: 6.3.2.0646		Date	: 03.08.2020 08:21:15
Operator	: Messaccount		Sample Name	:
Sample Number	: 006		Study	:
AutoSampler	: BUILT-IN		Rack/Vial	: 0/6
Instrument Name	: GC Clarus 580		Channel	: A
Instrument Serial #	: 580S12092501		A/D mV Range	: 1000
Delay Time	: 0,00 min		End Time	: 20,00 min
Sampling Rate	: 12,5000 pts/s			
Sample Volume	: 1,000000 ul			
Sample Amount	: 1,0000		Area Reject	: 0,000000
Data Acquisition Time	: 31.07.2020 18:29:38		Dilution Factor	: 1,00
			Cycle	: 6

Raw Data File : C:\Analytik\daten\trg-1(4chlorphenyl)ethanol_3107.raw
Result File : C:\Analytik\daten\trg-1(4chlorphenyl)ethanol_3107-20200803-081325.rst [Editing in Progress]
Inst Method : C:\Analytik\methoden\STANDARD_4K_aufheizrate from C:\Analytik\daten\trg-1(4chlorphenyl)ethanol_3107.raw
Proc Method : C:\Analytik\methoden\STANDARD_4K_aufheizrate.mth from C:\Analytik\daten\trg-1(4chlorphenyl)ethanol_3107-20200803-081325.rst [Editing in Progress]
Calib Method : C:\Analytik\methoden\STANDARD_4K_aufheizrate.mth from C:\Analytik\daten\trg-1(4chlorphenyl)ethanol_3107-20200803-081325.rst [Editing in Progress]
Report Format File: C:\Analytik\methoden\STANDARD_4K_aufheizrate.rpt
Sequence File : C:\Analytik\sequenzen\trg231072020.seq

DEFAULT REPORT

Peak #	Time [min]	Area [µV·s]	Height [µV]	Area [%]	Norm. Area [%]	BL	Area/Height [s]
1	2,357	41,15	52,09	0,00	0,00	BB	0,7899
2	2,430	120,59	66,10	0,01	0,01	BB	1,8243
3	3,213	804,54	380,94	0,06	0,06	BV	2,1120
4	3,288	1304055,98	549429,26	99,51	99,51	VV	2,3735
5	3,531	400,98	185,60	0,03	0,03	VB	2,1604
6	3,735	748,20	350,76	0,06	0,06	BB	2,1331
7	15,282	4330,54	962,46	0,33	0,33	BB	4,4995
8	15,920	37,56	53,15	0,00	0,00	BB	0,7066
		1310539,54	551480,37	100,00	100,00		

Missing Component Report

Component	Expected Retention (Calibration File)

All components were found

Figure S45: GC chromatogram of catalysis #7 after 60 min.

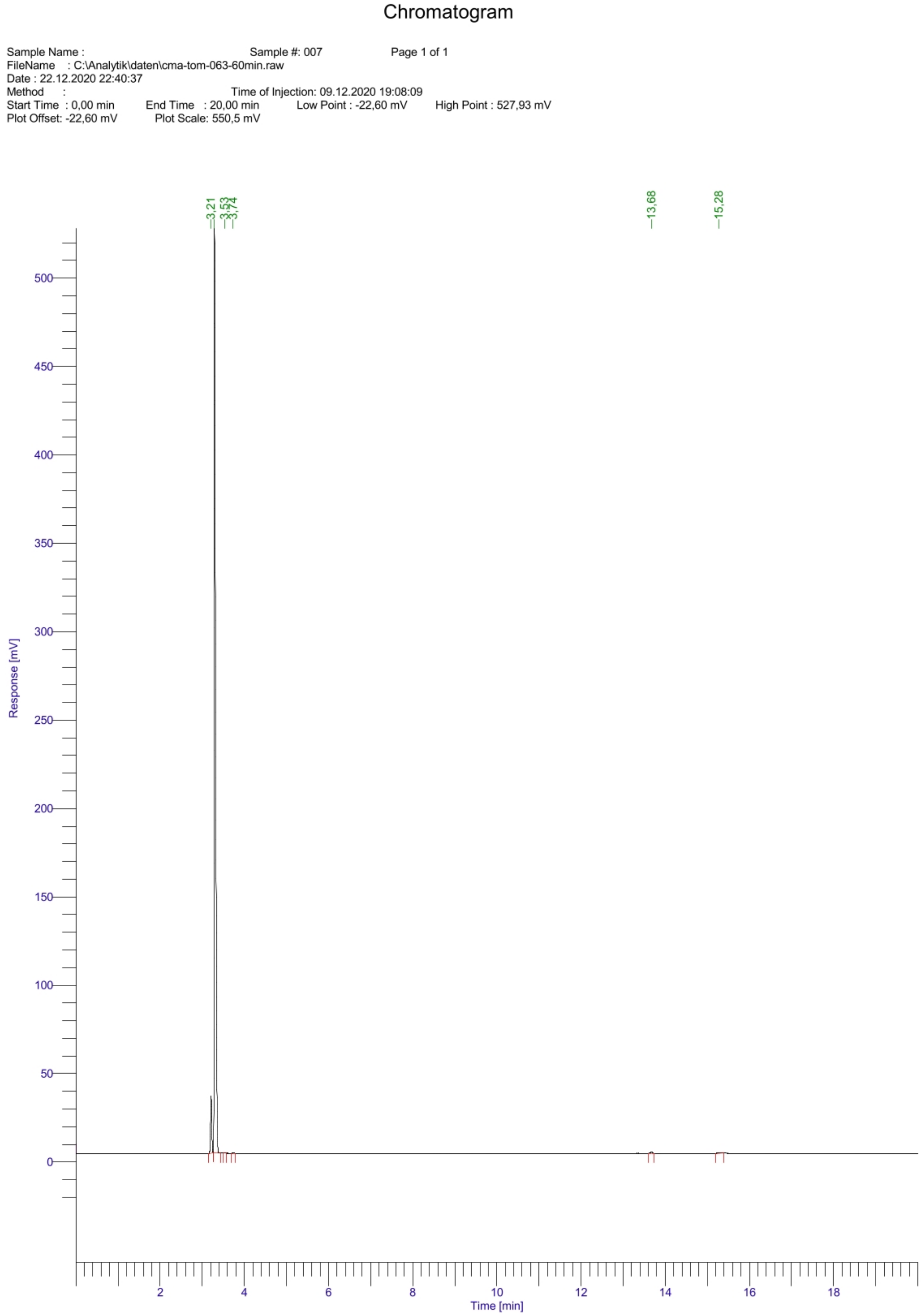

Software Version	: 6.3.2.0646		Date	: 10.12.2020 08:31:57
Operator	: Messaccount		Sample Name	:
Sample Number	: 007		Study	:
AutoSampler	: BUILT-IN		Rack/Vial	: 0/7
Instrument Name	: GC Clarus 580		Channel	: A
Instrument Serial #	: None		A/D mV Range	: 1000
Delay Time	: 0,00 min		End Time	: 20,00 min
Sampling Rate	: 12,5000 pts/s			
Sample Volume	: 1,000000 ul		Area Reject	: 0,000000
Sample Amount	: 1,0000		Dilution Factor	: 1,00
Data Acquisition Time	: 09.12.2020 19:08:09		Cycle	: 7

Raw Data File : C:\Analytik\daten\cma-tom-063-60min.raw
Result File : C:\Analytik\daten\cma-tom-063-60min-20201210-082754.rst [Editing in Progress]
Inst Method : C:\Analytik\methoden\STANDARD_4K_aufheizrate from
C:\Analytik\daten\cma-tom-063-60min.raw
Proc Method : C:\Analytik\methoden\STANDARD_4K_aufheizrate.mth from
C:\Analytik\daten\cma-tom-063-60min-20201210-082754.rst [Editing in Progress]
Calib Method : C:\Analytik\methoden\STANDARD_4K_aufheizrate.mth from
C:\Analytik\daten\cma-tom-063-60min-20201210-082754.rst [Editing in Progress]
Report Format File: C:\Analytik\methoden\STANDARD_4K_aufheizrate.rpt
Sequence File : C:\Analytik\sequenzen\cmann09122020.seq

DEFAULT REPORT

Peak #	Time [min]	Area [µV·s]	Height [µV]	Area [%]	Norm. Area [%]	BL	Area/Height [s]
1	3,210	64819,37	32106,97	4,98	4,98	BB	2,0189
2	3,290	1229574,77	523503,07	94,56	94,56	BB	2,3487
3	3,532	295,72	154,12	0,02	0,02	BB	1,9188
4	3,736	727,63	336,25	0,06	0,06	BB	2,1640
5	13,678	3109,39	899,13	0,24	0,24	BB	3,4582
6	15,276	1782,37	405,07	0,14	0,14	BB	4,4002
		1300309,24	557404,60	100,00	100,00		

Missing Component Report

Component	Expected Retention (Calibration File)

All components were found

2.8 Table 1, Entry #8

Figure S46: GC chromatogram of Cyclohexanone.

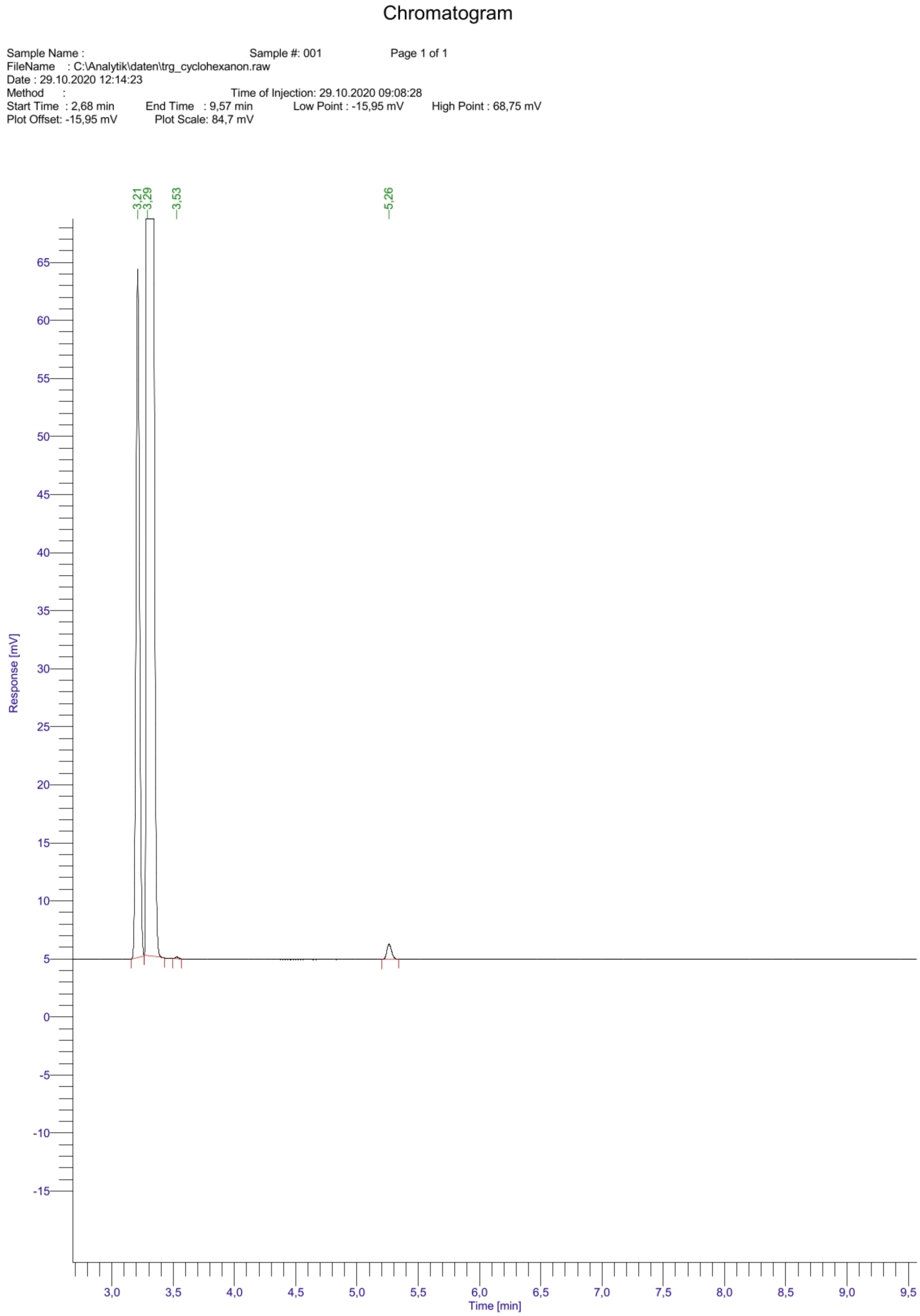

Software Version	: 6.3.2.0646	Date	: 29.10.2020 12:13:15
Operator	: Messaccount	Sample Name	:
Sample Number	: 001	Study	:
AutoSampler	: BUILT-IN	Rack/Vial	: 0/20
Instrument Name	: GC Clarus 580	Channel	: A
Instrument Serial #	: None	A/D mV Range	: 1000
Delay Time	: 0,00 min	End Time	: 20,00 min
Sampling Rate	: 12,5000 pts/s		
Sample Volume	: 1,000000 ul		
Sample Amount	: 1,0000	Area Reject	: 0,000000
Data Acquisition Time	: 29.10.2020 09:08:28	Dilution Factor	: 1,00
		Cycle	: 1

Raw Data File : C:\Analytik\daten\trg_cyclohexanon.raw
Result File : C:\Analytik\daten\trg_cyclohexanon-20201029-121208.rst [Editing in Progress]
Inst Method : C:\Analytik\methoden\STANDARD_4K_aufheizrate from
C:\Analytik\daten\trg_cyclohexanon.raw
Proc Method : C:\Analytik\methoden\STANDARD_4K_aufheizrate.mth from
C:\Analytik\daten\trg_cyclohexanon-20201029-121208.rst [Editing in Progress]
Calib Method : C:\Analytik\methoden\STANDARD_4K_aufheizrate.mth from
C:\Analytik\daten\trg_cyclohexanon-20201029-121208.rst [Editing in Progress]
Report Format File: C:\Analytik\methoden\STANDARD_4K_aufheizrate.rpt
Sequence File : C:\Analytik\sequenzen\trg29102020.seq

DEFAULT REPORT

Peak #	Time [min]	Area [µV·s]	Height [µV]	Area [%]	Norm. Area [%]	BL	Area/Height [s]
1	3,211	119765,00	59246,96	9,40	9,40	BB	2,0215
2	3,292	1150787,99	495884,55	90,30	90,30	BB	2,3207
3	3,530	266,30	138,40	0,02	0,02	BB	1,9241
4	5,262	3610,87	1314,87	0,28	0,28	BB	2,7462
		1274430,17	556584,77	100,00	100,00		

Missing Component Report

Component	Expected Retention (Calibration File)

All components were found

Figure 47: GC chromatogram of Cyclohexanol.

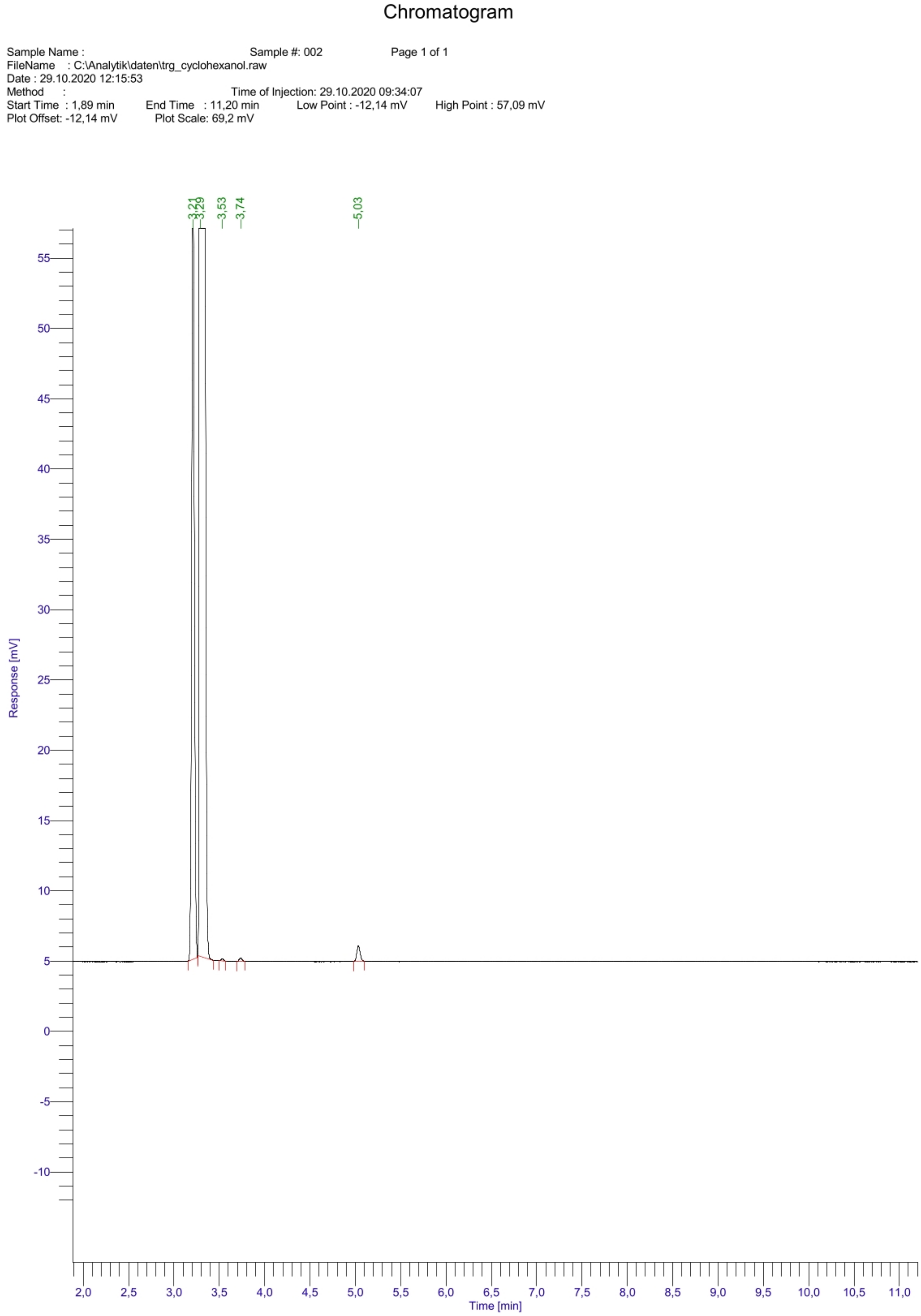

Software Version	: 6.3.2.0646	Date	: 29.10.2020 12:15:05
Operator	: Messaccount	Sample Name	:
Sample Number	: 002	Study	:
AutoSampler	: BUILT-IN	Rack/Vial	: 0/21
Instrument Name	: GC Clarus 580	Channel	: A
Instrument Serial #	: None	A/D mV Range	: 1000
Delay Time	: 0,00 min	End Time	: 20,00 min
Sampling Rate	: 12,5000 pts/s		
Sample Volume	: 1,000000 ul	Area Reject	: 0,000000
Sample Amount	: 1,0000	Dilution Factor	: 1,00
Data Acquisition Time	: 29.10.2020 09:34:07	Cycle	: 2

Raw Data File : C:\Analytik\daten\trg_cyclohexanol.raw
Result File : C:\Analytik\daten\trg_cyclohexanol-20201029-121208.rst [Editing in Progress]
Inst Method : C:\Analytik\methoden\STANDARD_4K_aufheizrate from
C:\Analytik\daten\trg_cyclohexanol.raw
Proc Method : C:\Analytik\methoden\STANDARD_4K_aufheizrate.mth from
C:\Analytik\daten\trg_cyclohexanol-20201029-121208.rst [Editing in Progress]
Calib Method : C:\Analytik\methoden\STANDARD_4K_aufheizrate.mth from
C:\Analytik\daten\trg_cyclohexanol-20201029-121208.rst [Editing in Progress]
Report Format File: C:\Analytik\methoden\STANDARD_4K_aufheizrate.rpt
Sequence File : C:\Analytik\sequenzen\trg29102020.seq

DEFAULT REPORT

Peak #	Time [min]	Area [µV·s]	Height [µV]	Area [%]	Norm. Area [%]	BL	Area/Height [s]
1	3,209	115799,32	57011,11	8,93	8,93	BB	2,0312
2	3,291	1177559,03	506116,23	90,79	90,79	BB	2,3267
3	3,531	287,98	141,16	0,02	0,02	BB	2,0401
4	3,736	545,02	250,47	0,04	0,04	BB	2,1760
5	5,034	2843,25	1101,59	0,22	0,22	BB	2,5810
		1297034,60	564620,56	100,00	100,00		

Missing Component Report

Component	Expected Retention (Calibration File)

All components were found

Figure S48: GC chromatogram of catalysis #8 after 90 min.

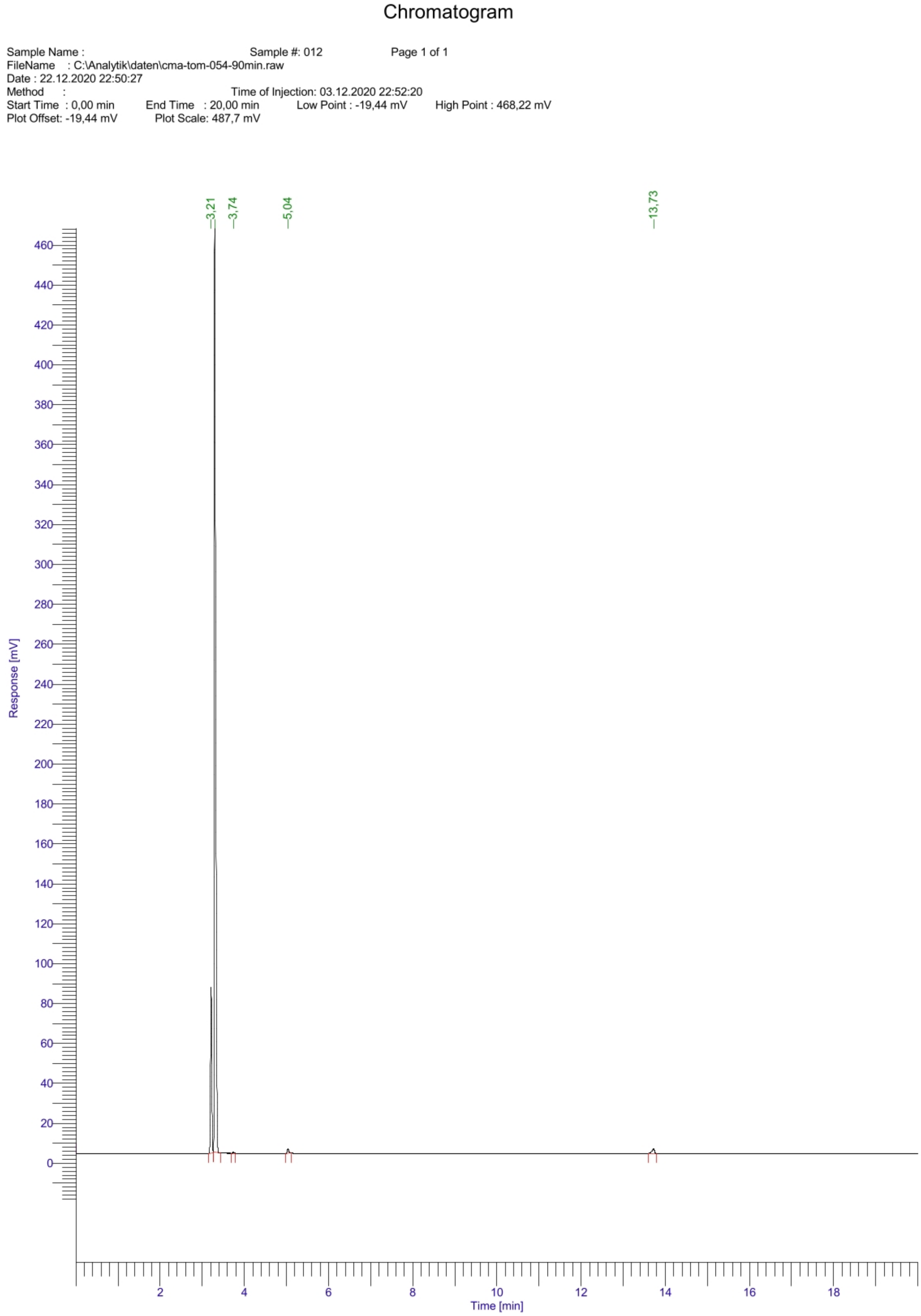

Software Version	: 6.3.2.0646		Date	:	04.12.2020 13:08:07
Operator	: Messaccount		Sample Name	:	
Sample Number	: 012		Study	:	
AutoSampler	: BUILT-IN		Rack/Vial	:	0/12
Instrument Name	: GC Clarus 580		Channel	:	A
Instrument Serial #	: None		A/D mV Range	:	1000
Delay Time	: 0,00 min		End Time	:	20,00 min
Sampling Rate	: 12,5000 pts/s				
Sample Volume	: 1,000000 ul				
Sample Amount	: 1,0000		Area Reject	:	0,000000
Data Acquisition Time	: 03.12.2020 22:52:20		Dilution Factor	:	1,00
			Cycle	:	12

Raw Data File : C:\Analytik\daten\cma-tom-054-90min.raw
Result File : C:\Analytik\daten\cma-tom-054-90min-20201204-125816.rst [Editing in Progress]
Inst Method : C:\Analytik\methoden\STANDARD_4K_aufheizrate from C:\Analytik\daten\cma-tom-054-90min.raw
Proc Method : C:\Analytik\methoden\STANDARD_4K_aufheizrate.mth from C:\Analytik\daten\cma-tom-054-90min-20201204-125816.rst [Editing in Progress]
Calib Method : C:\Analytik\methoden\STANDARD_4K_aufheizrate.mth from C:\Analytik\daten\cma-tom-054-90min-20201204-125816.rst [Editing in Progress]
Report Format File: C:\Analytik\methoden\STANDARD_4K_aufheizrate.rpt
Sequence File : C:\Analytik\sequenzen\cmann03122020.seq

DEFAULT REPORT

Peak #	Time [min]	Area [µV·s]	Height [µV]	Area [%]	Norm. Area [%]	BL	Area/Height [s]
1	3,209	169872,45	82762,08	13,57	13,57	BB	2,0525
2	3,293	1066343,25	462867,35	85,18	85,18	BB	2,3038
3	3,736	998,64	464,83	0,08	0,08	BB	2,1484
4	5,037	5513,32	2117,01	0,44	0,44	BB	2,6043
5	13,726	9215,90	2069,52	0,74	0,74	BB	4,4531
		1251943,55	550280,79	100,00	100,00		

Missing Component Report

Component	Expected Retention (Calibration File)

All components were found

3. Crystallographic Data

Figure S49: Molecular structure of (η^5-Cyclopentadienyl)(η^6-1-phenyl-4-methyl-1*H*-imidazol)iron(II) Hexafluorophosphate (**2a**) in the solid state.

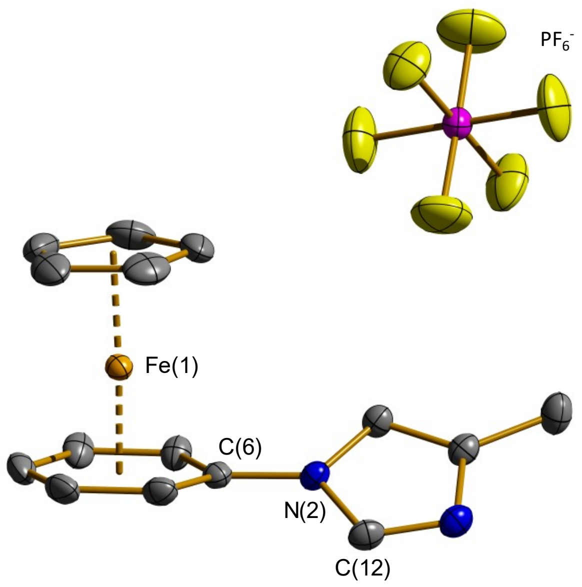

Figure S50: Molecular structure of (η^5-Cyclopentadienyl)(η^6-1-phenyl-3-methylimidazolium)iron(II) Bis(hexafluorophosphate) (**3****) in the solid state.

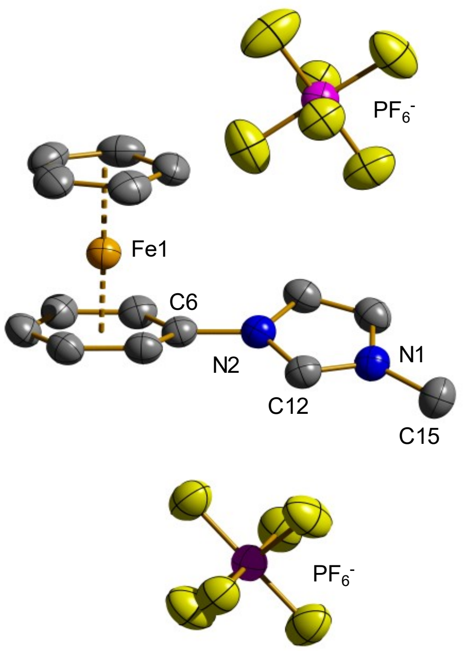

Table S1: Crystallographic data, data collection and refinement.

	**2**	**2a**	**3**	**3a**	**4**
empirical formula	$C_{14}H_{13}F_6FeN_2P$	$C_{15}H_{15}F_6FeN_2P$	$C_{15}H_{16}FeI_2N_2$	$C_{15}H_{16}F_{12}FeN_2P_2$	$C_{25}H_{29}F_6FeIIrN_2P$
CDCC number	2092872	2092873	2092874	2092875	2092876
formula weight	410.08	424.11	533.95	570.08	877.42
crystal size [mm]	0.560·0.510·0.360	0.192·0.190·0.053	0.414·0.354·0.183	0.415·0.360·0.287	0.166·0.130·0.047
T [K]	150(2)	150(2)	150(2)	150(2)	100(2)
λ [Å]	0.71073	0.71073	1.54184	1.54184	0.71073
crystal system	Monoclinic	Monoclinic	Orthorhombic	Triclinic	Triclinic
space group	$P2_1/n$	$P2_1/c$	Pbca	P-1	P-1
a [Å]	11.0382(3)	10.5042(3)	10.2038(2)	8.7451(3)	11.861(3)
b [Å]	10.5517/3)	10.3854(2)	12.7228(2)	10.5359(5)	13.452(3)
c [Å]	14.2240(4)	15.1744(7)	26.7855(5)	14.2904(6)	13.704(3)
α [°]	90	90	90	105.390(4)	79.644(8)
β [°]	112.735(3)	109.220(3)	90	100.610(3)	64.768(7)
γ [°]	90	90	90	108.585(4)	88.037(8)
V [$Å^3$]	1527.97(8)	1563.11(7)	3477.32(11)	1150.08(9)	1943.5(8)
Z	4	4	8	2	2
$\rho_{calcd.}$ [g cm^3]	1.783	1.802	2.040	1.823	1.499
μ [mm^{-1}]	1.156	1.134	34.764	1.823	4.670
F(000)	824	856	2016	632	836
Θ-range [°]	2.780-32.398	2.840-32.415	5.450-62.696	3.356-62.745	1.541-33.728
refl. coll.	10634	16927	24114	9733	203114
indep. refl.	4997	5202	2782	3650	15541
	[R(int) = 0.0267]	[R(int) = 0.0314]	[R(int) = 0.0764]	[R(int) = 0.0267]	[R(int) = 0.0714]
data/restr./param.	4997 / 192 / 254	5202 / 0 / 227	2782 / 0 / 182	3650 / 0 / 326	15541 / 0 / 340
final *R* indices [$I>2\sigma(I)$] [a]	0.0405, 0.0918	0.0430, 0.0912	0.0430, 0.1108	0.0438, 0.1219	0.0258, 0.0585
R indices (all data)	0.0517, 0,0975	0.0578, 0.0971	0.0462, 0.1140	0.0442, 0.1224	0.0337, 0.0613
GooF [b]	1.048	1.048	1.050	1.031	1.037
$\Delta\rho_{max/min}$ (e·$Å^{-3}$)	0.532/-0.555	0.620/-0.433	1.741/-1.225	0.633/-0.457	1.553/-1.798

[a] $R1 = \Sigma||F_o| - |F_c||/\Sigma|F_o|$, $\omega R2 = [\Sigma\omega(F_o^2 - F_c^2)2/\Sigma\omega F_o^2]^{1/2}$. [b] $GooF = [\Sigma\omega(F_o^2 - F_c^2)2/(n-p)]^{1/2}$.

4. Computational Study

Table S2: Selected DFT-calculated bond parameters for Ir-complexes. Distances in Å.

	A	B	C	D	E	F	G	H
**Ir-O1**	2.075	2.134	6.900	1.938	2.128	2.237	1.938	2.159/2.155
**Ir-H**	3.306	1.870	1.599	2.978	1.705	1.595	3.051	3.203
**Ir-Cp**	2.249-2.348	2.173-3.422	2.285-2.329	2.186-2.257	2.195-2.282	2.155-2.356	2.198-2.263	2.197-2.291
**Ir-C_{NHC}**	2.009	1.939	2.006	2.080	2.079	2.056	2.085	2.069
**Ir-C_{Ph}**	2.042	2.025	2.035	3.521	3.690	3.729	3.559	3.652
**Fe-$Cp_{centroid}$**	1.702	1.700	1.698	1.710	1.711	1.710	1.710	1.710
**Fe-$C6_{centroid}$**	1.642	1.616	1.623	1.606	1.608	1.610	1.612	1.613

Figure S51: Optimized structures along the β-hydrogen transfer paths **D'-E'-F'**. For this path, a proton has been transferred to the imidazolylidene ring.

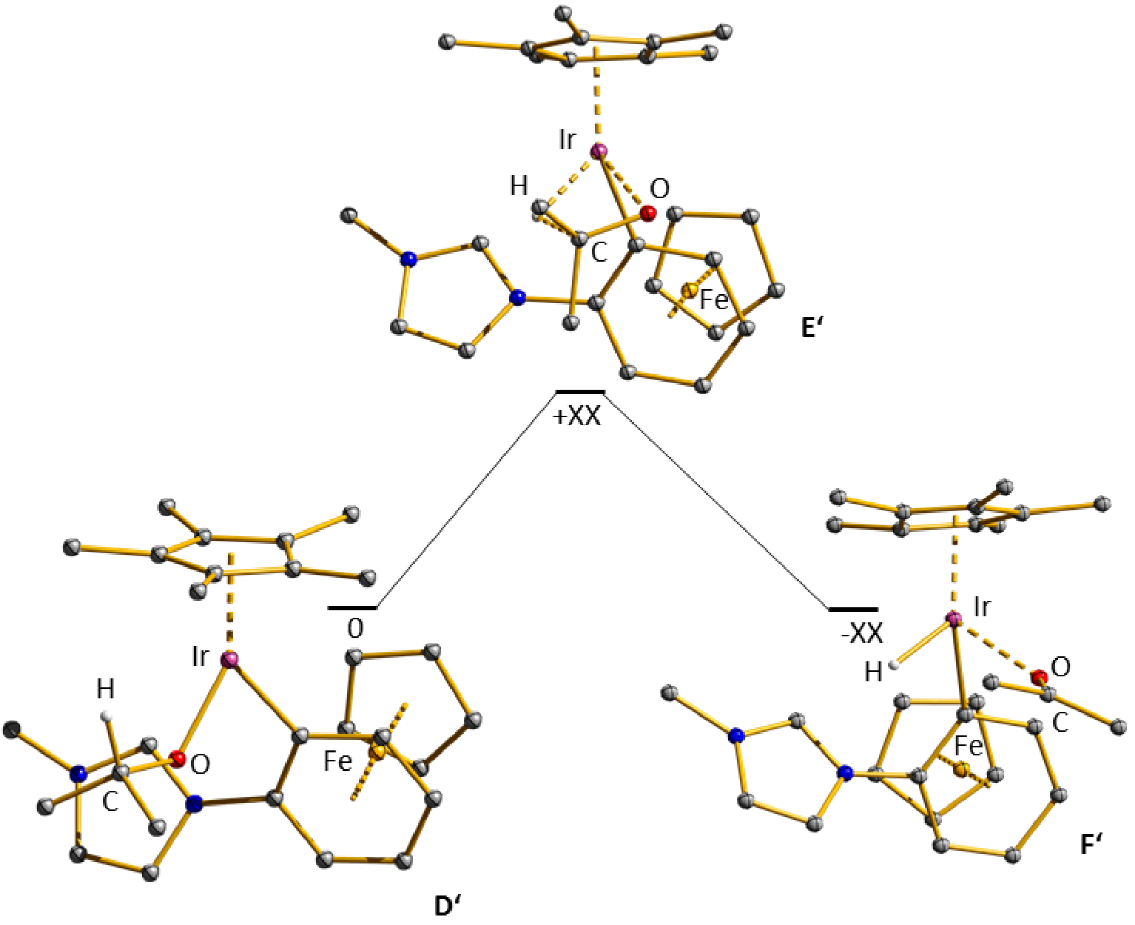

5. Deuteration Experiment

Figure S52: 2H NMR spectrum of the deuteration experiment with compound

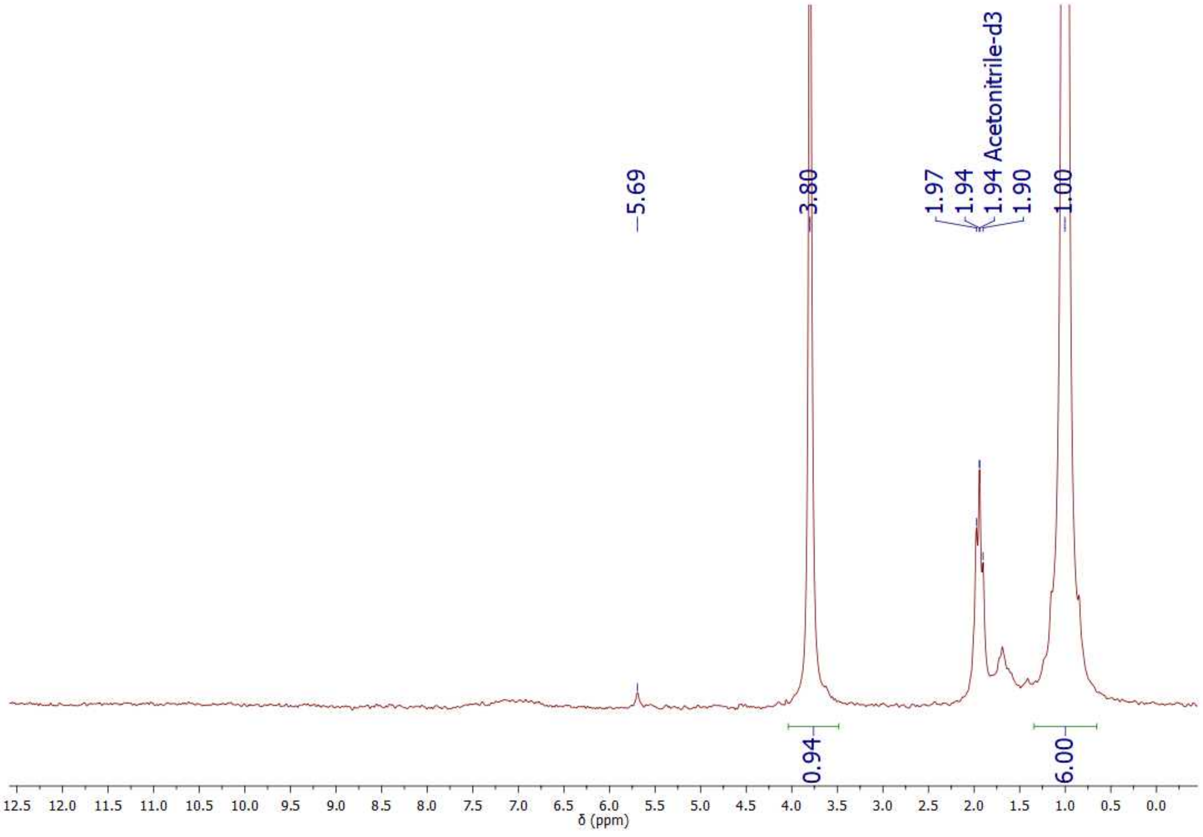

.
